# Selective Oxidative Cleavage of Benzyl C–N Bond under Metal-Free Electrochemical Conditions

**DOI:** 10.3390/molecules29122851

**Published:** 2024-06-15

**Authors:** Jiawei Huang, Xiaoman Li, Ping Liu, Yu Wei, Shuai Liu, Xiaowei Ma

**Affiliations:** 1 State Key Laboratory Incubation Base for Green Processing of Chemical Engineering, School of Chemistry and Chemical Engineering, Shihezi University, Shihezi 832003, China; huangjiawei@stu.shzu.edu.cn (J.H.); lixiaoman@stu.shzu.edu.cn (X.L.); liuping@shzu.edu.cn (P.L.); yuweichem@shzu.edu.cn (Y.W.); 2 Bingtuan Energy Development Institute, Shihezi University, Shihezi 832003, China

**Keywords:** electrochemical oxidation, C–N bond cleavage, amine, aldehyde, ketone

## Abstract

With the growing significance of green chemistry in organic synthesis, electrochemical oxidation has seen rapid development. Compounds undergo oxidation–reduction reactions through electron transfer at the electrode surface. This article proposes the use of electrochemical methods to achieve cleavage of the benzyl C–N bond. This method selectively oxidatively cleaves the C–N bond without the need for metal catalysts or external oxidants. Additionally, primary, secondary, and tertiary amines exhibit good adaptability under these conditions, utilizing water as the sole source of oxygen.

## 1. Introduction

**Scheme 1:**
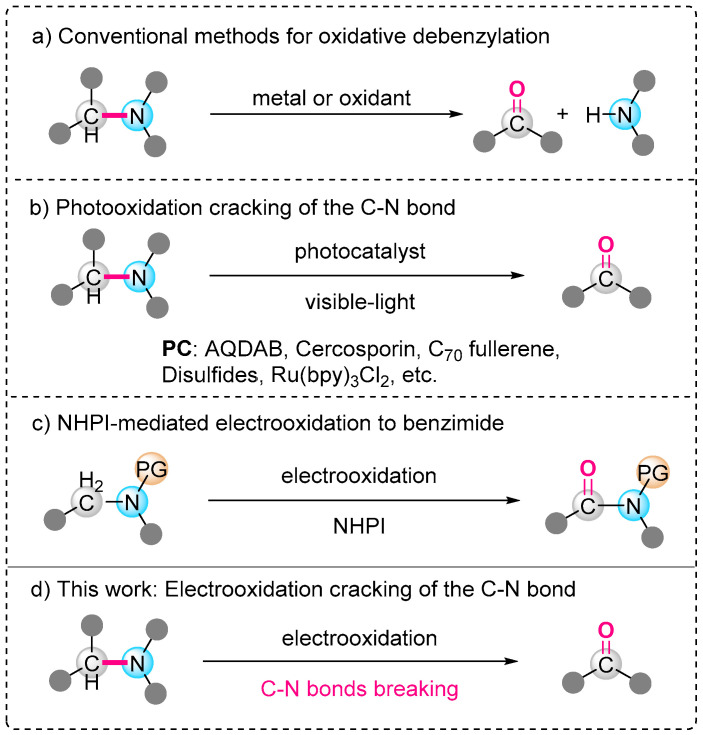
Research on the oxidation of amine compounds.

C(sp^3^)–H bond oxidation reactions are important for the preparation of high-value-added compounds, such as ketones, esters, amides, and aldehydes, from simple and readily available alkanes. They are an important class of transformations in organic synthesis [1,2,3,4,5,6,7]. Among them, aldehyde compounds are a class of highly valuable compounds in organic chemistry not only present in various natural products but also widely used in the synthesis of drugs and fine chemicals [8,9,10,11,12]. On the other hand, amines are a common class of compounds found in nature [13,14,15]. In recent years, oxidation-mediated C–N bond breaking of tertiary amines has been found to be able to generate secondary amines as well as aldehydes or ketones, which provides more options for amine conversion. In traditional methods, the oxidative cleavage of C–N bonds is mainly realized by transition metal catalysis or oxidants (Scheme 1a) [16,17,18,19,20,21,22,23,24,25,26]. With the increasing emphasis on green synthesis and transformation in organic chemistry, visible photocatalysis, which has the advantages of being green and clean, safe and environmentally friendly, and easy to control, has made breakthroughs in the field of synthetic chemistry. In recent years, a variety of visible light-catalyzed methods for oxidative C–N cleavage carbonylation have emerged (Scheme 1b). This method can be catalyzed by AQDAB [27], Cercosporin [28], C_70_ fullerene [29], disulfides [30], Ru(bpy)_3_Cl_2_ [31], etc. [32,33] under visible light irradiation. However, some drawbacks, such as more complicated reaction conditions and the drawbacks of amplification of photocatalysis, still limit their large-scale application. Therefore, the search for new “green” oxidation processes [1,2,4,30,34,35,36] to achieve the oxidation cleavage of C–N bonds has become a hot topic in the development of new methods.

Organic electrochemical synthesis is a versatile and sustainable synthetic tool that utilizes renewable electrons as reaction reagents, enabling the direct oxidation–reduction reactions of reactants at the electrode surface, successfully replacing traditional oxidation/reduction catalysts to achieve challenging transformations under milder reaction conditions. In the past few decades, electrochemical oxidation has rapidly developed, and organic electrosynthesis provides a novel and robust strategy for compound oxidation. It can substitute for some toxic reagents, reduce waste generation in reactions, lower environmental impact, and further drive the development of the field of “green synthesis” [37,38,39,40,41,42,43]. On this basis, a series of valuable amine compound electrochemical transformations have been developed [37,44], offering advantages over traditional metal catalysis or emerging visible light catalysis by eliminating the need for metal catalysts and oxidants. Partial research has also been conducted on the oxidation of amine compounds in organic electrochemistry. The Gu research group reported the electrochemical oxidation of benzamides mediated by NHPI to achieve α-oxidation (Scheme 1c) [45]. To further expand the application of amine compounds in organic electrochemistry and due to our continued interest in organic electrochemistry [46,47,48,49], we are interested in exploring whether the selective oxidation cleavage of C–N bonds can be achieved using organic electrochemistry. This article reports the validation of this hypothesis (Scheme 1d).

## 2. Results and Discussion

Initially, a model substrate of 4-tert-butylbenzylamine **1a** was selected to prove the feasibility of this hypothesis and optimize the reaction conditions. The electro-chemical reaction apparatus comprised an undivided cell with platinum (Pt) as the anode and cathode. After extensive screening, the desired products **2a** could be obtained with an isolated yield of 79% under the following conditions: constant current (I = 2 mA), electrolyte Et_4_NBF_4_ (0.15 mmol), additive TsOH·H_2_O (0.15 mmol), and solvent consisting of MeCN and H_2_O (2.0 mL:0.2 mL). The reaction was carried out at room temperature for 24 h (Entry 1, Table 1). Without an electric current, the reaction did not occur (Entry 2). The yield significantly decreased when TsOH·H_2_O was not used (Entry 3). When other acids such as TFA, PA, and AcOH were used instead of TsOH·H_2_O as additives, the reaction yield decreased slightly (Entries 4–6). Using a graphite electrode instead of a platinum electrode, the yield of **2a** decreased to 44% (Entry 7). Regulating the current to 4 mA slightly decreased the yield (Entry 8).

**Scheme 2:**
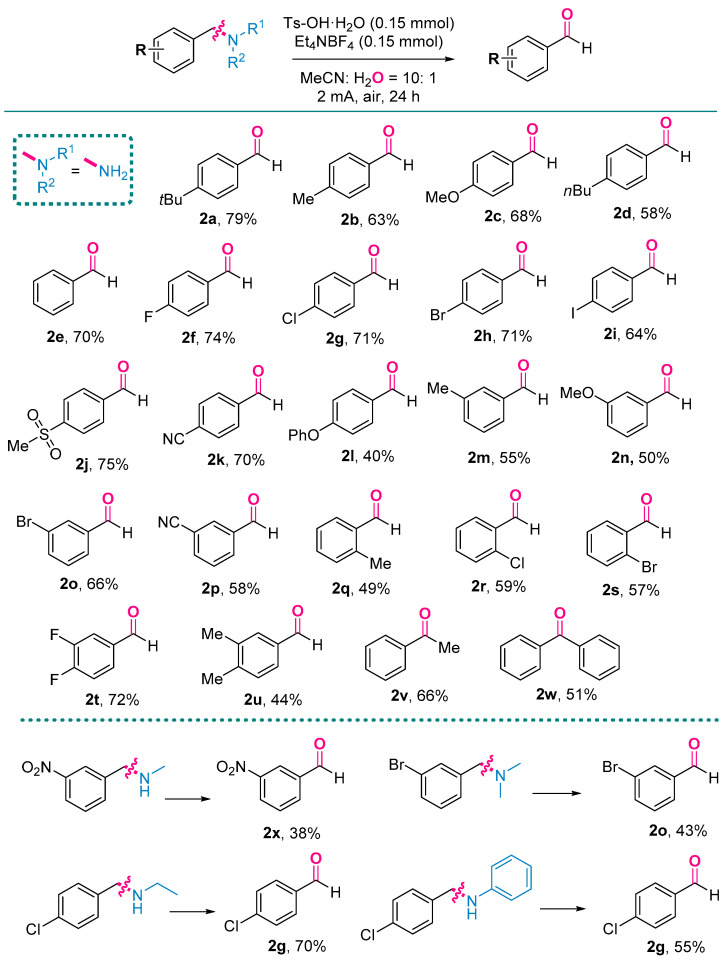
Substrate scope of benzylamine derivatives. **1** (0.3 mmol, 1.0 equiv.), TsOH·H_2_O (0.15 mmol, 0.5 equiv.), Et_4_NBF_4_ (0.15 mmol, 0.5 equiv.), and MeCN/H_2_O = (2.0 mL/0.2 mL); constant current = 2.0 mA in air for 24 h. Isolated yields.

With the optimal conditions in hand, the substrate scope of this protocol was investigated, and the results are illustrated in Scheme 2. Benzylamines bearing electron-donating groups (such as tert-butyl, methyl, and methoxy) and electron-withdrawing groups (such as halides, cyano, and sulfonyl) at the para position of the benzene ring are converted to the corresponding aldehyde compounds with moderate to good yields (**2a**–**2l**). When using ortho-bromo or meta-bromo benzylamine (**2o**, 66%; **2s**, 57%), compared to para-substituted benzylamine (**2h**, 71%), the yields significantly decreased. This may be due to the presence of steric hindrance in the reaction, which can prevent certain reactants with large sterically hindered groups from effectively approaching the reaction center or increase the interaction energy between reactant molecules, thereby reducing the reaction rates and leading to decreased yields. Disubstituted benzylamines such as 3,4-difluorobenzylamine (**2t**) and 3,4-dimethylbenzylamine (**1u**) also tolerate this transformation to afford the corresponding disubstituted aldehyde compounds. We also explored the conversion of α-branched benzylamines as substrates (**1v**, **1w**), when the α-substituent was an alkyl group, we obtained acetophenone (**2v**); when the α-substituent was an aryl group, the reaction also proceeded smoothly, providing the corresponding diaryl ketone (**2w**). Finally, when *N*-substituted and *N*,*N*-disubstituted benzylamines were treated, the cleavage of C–N bonds was not influenced by such substitution, resulting in the corresponding aldehydes (**2x**, **2o**, **2g**).

**Scheme 3:**
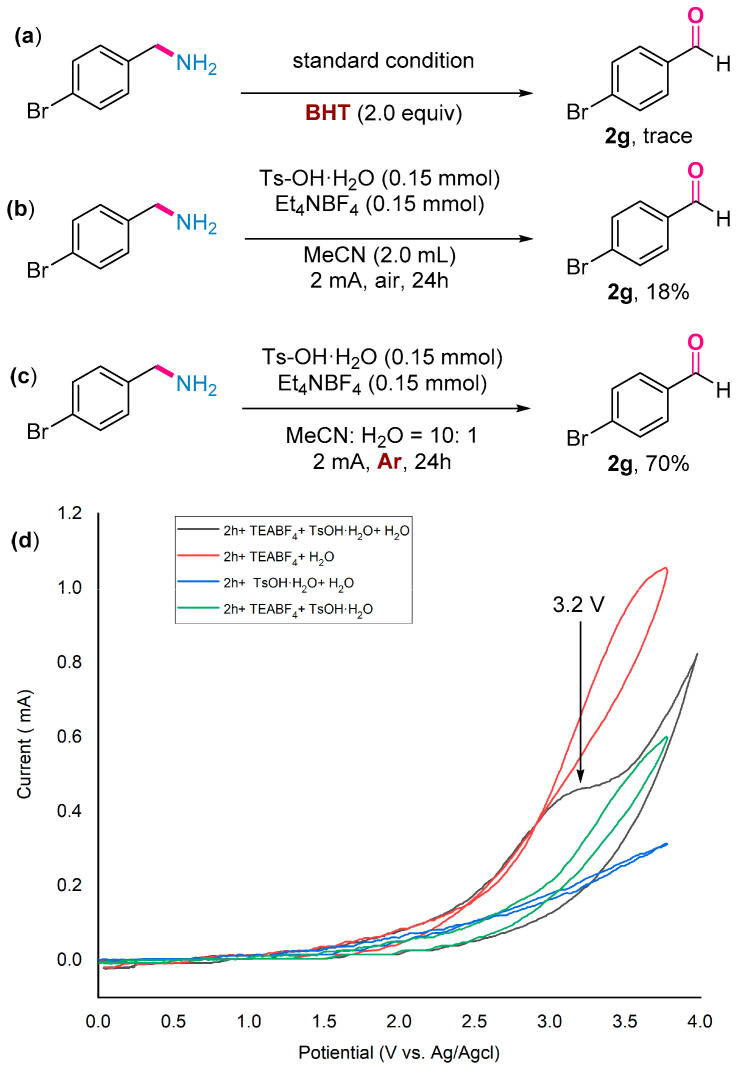
Mechanistic studies. (**a**) Radical trapping experiment. (**b**,**c**) The determination of oxygen source. (**d**) Cyclic voltammetry for benzylamine **2h**.

In order to further explore the reaction mechanism, the following control experiments (Scheme 3) were performed. First, butylhydroxytoluene (BHT), a widely used free radical inhibitor, was added under standard conditions, and although we were not able to detect any radical intermediates, the yield of the **2a** was reduced to 5% (Scheme 3a), which implied the possible involvement of radical species in the reaction. To further investigate the source of oxygen, we attempted the reaction using only MeCN as the solvent, and the reaction yield was significantly affected (Scheme 3b). Subsequently, we carried out the reaction under an argon atmosphere, and the reaction yield was hardly affected (Scheme 3c). Meanwhile, cyclic voltammetry experiments showed that the addition of TsOH·H_2_O and H_2_O was able to cause a significant emergent oxidation peak of benzylamine at 3.2 V. (Scheme 3). Based on the above experimental results, it is believed that the oxygen source in this reaction is derived from water.

**Scheme 4:**
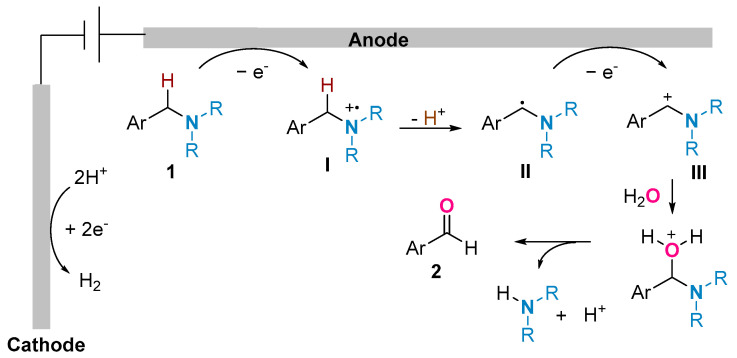
A proposed mechanism for the C–N bond cleavage reaction.

Based on the literature reports and experimental results [19,27,31,45,50,51], we propose a possible reaction mechanism (Scheme 4). First, benzylamine undergoes a single-electron oxidation process at the anode to form the nitrogen radical cationic intermediate **I**. Intermediate **I** undergoes deprotonation and radical 1,2-migration to give the α-amino-alkyl radical intermediate **II**. Subsequently, intermediate **II** was oxidized at the anode to form the benzyl-carbocation intermediate **III**, which was then captured by H_2_O. The final dissociation of the amine group facilitated by TsOH·H_2_O yielded the corresponding carbonyl target compound.

## 3. Materials and Methods

### 3.1. Materials and Instruments

Unless otherwise noted, all reactions were carried out under an O_2_ atmosphere. Analytical thin-layer chromatography (TLC) was performed on glass plates coated with 0.25 mm 230–400 mesh silica gel containing a fluorescent indicator. Visualization was accomplished by exposure to a UV lamp. All the products in this article are compatible with standard silica gel chromatography. Column chromatography was performed on silica gel (200–300 mesh) using standard methods. NMR spectra were measured on a Bruker Ascend 400 spectrometer (Billerica, MA, USA) and chemical shifts (δ) are reported in parts per million (ppm). ^1^H NMR spectra were recorded at 400 MHz in NMR solvents and referenced internally to corresponding solvent resonance, and ^13^C NMR spectra were recorded at 101 MHz and referenced to corresponding solvent resonance. Coupling constants are reported in Hz with multiplicities denoted as s (singlet), d (doublet), t (triplet), q (quartet), m (multiplet), and br (broad). Commercial reagents and solvent were purchased from Adamas (Guildford, UK), J&K (Beijing, China), Energy (Shanghai, China), Sigma-Aldrich (St. Louis, MO, USA), Alfa Aesar (Haverhill, MA, USA), Acros Organics (Waltham, MA, USA), TCI (Tokyo, Japan) and used as received unless otherwise stated.

### 3.2. The General Procedure for Electrochemical Oxidation Cracking of C–N Bond

Benzylamine (0.3 mmol), Ts-OH·H_2_O (0.15 mmol), Et_4_NBF_4_ (0.15 mmol), MeCN (2.0 mL), and H_2_O (0.2 mL) were added to a dried 15 mL reaction vial. Then, two electrodes with platinum plates were immerged into the solution, as the anode and cathode. The electrodes were connected to a constant current power supply, and the current was set as 2 mA. The reaction mixture was stirred at room temperature for 24 h under air conditions. The reaction mixture was then diluted with ethyl acetate. The combined organic phase was concentrated in vacuum on a rotary evaporator. The resulting residue was purified by silica gel flash chromatography, eluting with petroleum ether/ethyl acetate (10:1) to afford the desired products.

### 3.3. Characterization Data of Products

*4-(Tert-butyl)benzaldehyde* (**2a**) [27]. Following the general procedure with 4-(tert-butyl)benzaldehyde (48.9 mg, 0.3 mmol), **2a** was obtained as a white solid (38.4 mg, 79%). This target product was purified by column chromatography on silica gel (PE:EA = 10:1). ^1^H NMR (400 MHz, CDCl_3_) *δ* 9.98 (s, 1H), 7.81 (d, *J* = 8.4 Hz, 2H), 7.55 (d, *J* = 8.4 Hz, 2H), 1.35 (s, 9H). ^13^C NMR (101 MHz, CDCl_3_) *δ* 192.1, 158.4, 134.1, 129.7, 126.0, 35.3, 31.1.

*4-Methylbenzaldehyde* (**2b**) [27]. Following the general procedure with p-tolylmethanamine (36.3 mg, 0.3 mmol), **2b** was obtained as a colorless oil (22.7 mg, 63%). This target product was purified by column chromatography on silica gel (PE:EA = 10:1). ^1^H NMR (400 MHz, CDCl_3_) *δ* 9.96 (s, 1H), 7.77 (d, *J* = 8.0 Hz, 2H), 7.33 (d, *J* = 8.0 Hz, 2H), 2.44 (s, 3H). ^13^C NMR (101 MHz, CDCl_3_) *δ* 192.0, 145.5, 134.2, 129.8, 129.7, 21.9.

*4-Methoxybenzaldehyde* (**2c**) [27]. Following the general procedure with (4-methoxyphenyl)methanamine (41.1 mg, 0.3 mmol), **2c** was obtained as a colorless oil (27.7 mg, 68%). This target product was purified by column chromatography on silica gel (PE:EA = 10:1). ^1^H NMR (400 MHz, CDCl_3_) δ 9.87 (s, 1H), 7.82 (d, *J* = 8.8 Hz, 2H), 6.99 (d, *J* = 8.8 Hz, 2H), 3.87 (s, 3H). ^13^C NMR (101 MHz, CDCl_3_) δ 190.8, 164.6, 131.9, 129.9, 114.3, 55.5.

*4-Nutylbenzaldehyde* (**2d**) [27]. Following the general procedure with (4-butylphenyl)methanamine (48.9 mg, 0.3 mmol), **2d** was obtained as a colorless oil (28.2 mg, 58%). This target product was purified by column chromatography on silica gel (PE:EA = 10:1). ^1^H NMR (400 MHz, CDCl_3_) *δ* 9.97 (s, 1H), 7.79 (d, *J* = 7.2 Hz, 2H), 7.33 (d, *J* = 7.6 Hz, 2H), 2.69 (t, *J* = 7.6 Hz, 2H), 1.67–1.58 (m, 2H), 1.44–1.31 (m, 2H), 0.93 (t, *J* = 7.2 Hz, 3H). ^13^C NMR (101 MHz, CDCl_3_) *δ* 192.0, 150.4, 134.4, 129.8, 129.0, 35.9, 33.2, 22.3, 13.8.

*Benzaldehyde* (**2e**) [52]. Following the general procedure with phenylmethanamine (32.1 mg, 0.3 mmol), **2e** was obtained as a colorless oil (22.3 mg, 70%). This target product was purified by column chromatography on silica gel (PE:EA = 10:1). ^1^H NMR (400 MHz, CDCl_3_) *δ* 10.02 (s, 1H), 7.93–7.83 (m, 2H), 7.65–7.59 (m, 1H), 7.57–7.44 (m, 2H). ^13^C NMR (101 MHz, CDCl_3_) *δ* 192.4, 136.4, 134.5, 129.8, 129.0.

*4-Fluorobenzaldehyde* (**2f**) [52]. Following the general procedure with (4-fluorophenyl)methanamine (37.5 mg, 0.3 mmol), **2f** was obtained as a colorless oil (27.5 mg, 74%). This target product was purified by column chromatography on silica gel (PE:EA = 10:1). ^1^H NMR (400 MHz, CDCl_3_) *δ* 9.99 (s, 1H), 7.96–7.88 (m, 2H), 7.29–7.20 (m, 2H). ^13^C NMR (101 MHz, CDCl_3_) *δ* 190.5, 166.5 (d, *J* = 256.7 Hz), 132.8 (d, *J* = 9.4 Hz), 132.2 (d, *J* = 9.7 Hz), 116.4 (d, *J* = 22.3 Hz). ^19^F NMR (376 MHz, CDCl_3_) *δ* –102.39.

*4-Chlorobenzaldehyde* (**2g**) [27]. Following the general procedure with (4-chlorophenyl)methanamine (42.3 mg, 0.3 mmol), **2g** was obtained as a white solid (29.8 mg, 71%). Following the general procedure with *N*-(4-chlorobenzyl)ethanamine (50.7 mg, 0.3 mmol), **2g** was obtained as a white solid (29.4 mg, 70%). Following the general procedure with *N*-(4-chlorobenzyl)aniline (65.1 mg, 0.3 mmol), **2g** was obtained as a white solid (23.1 mg, 55%). This target product was purified by column chromatography on silica gel (PE:EA = 10:1). ^1^H NMR (400 MHz, CDCl_3_) *δ* 9.98 (s, 1H), 7.82 (d, *J* = 8.4 Hz, 2H), 7.51 (d, *J* = 8.0 Hz, 2H). ^13^C NMR (101 MHz, CDCl_3_) *δ* 190.8, 140.9, 134.7, 130.9, 129.4.

*4-Bromobenzaldehyde* (**2h**) [27]. Following the general procedure with (4-bromophenyl)methanamine (55.8 mg, 0.3 mmol), **2h** was obtained as a white solid (39.0 mg, 71%). This target product was purified by column chromatography on silica gel (PE:EA = 10:1). ^1^H NMR (400 MHz, CDCl_3_) *δ* 9.98 (s, 1H), 7.75 (d, *J* = 8.4 Hz, 2H), 7.69 (d, *J* = 8.4 Hz, 2H). ^13^C NMR (101 MHz, CDCl_3_) *δ* 191.0, 135.1, 132.4, 130.9, 129.8.

*4-Iodobenzaldehyde* (**2i**) [27]. Following the general procedure with (4-iodophenyl)methanamine (69.9 mg, 0.3 mmol), **2i** was obtained as a white solid (44.5 mg, 64%). This target product was purified by column chromatography on silica gel (PE:EA = 10:1). ^1^H NMR (400 MHz, CDCl_3_) *δ* 9.95 (s, 1H), 7.91 (d, *J* = 8.0 Hz, 2H), 7.59 (d, *J* = 8.4 Hz, 2H). ^13^C NMR (101 MHz, CDCl_3_) *δ* 191.4, 138.4, 135.6, 130.8, 102.8.

*4-(Methylsulfonyl)benzaldehyde* (**2j**) [27]. Following the general procedure with (4-(methylsulfonyl)phenyl)methanamine (55.5 mg, 0.3 mmol), **2j** was obtained as a white solid (41.4 mg, 75%). This target product was purified by column chromatography on silica gel (PE:EA = 10:1). ^1^H NMR (400 MHz, CDCl_3_) *δ* 10.13 (s, 1H), 8.12 (d, *J* = 8.4 Hz, 2H), 8.08 (d, *J* = 8.4 Hz, 2H), 3.09 (s, 3H). ^13^C NMR (101 MHz, CDCl_3_) *δ* 190.7, 145.4, 139.7, 130.4, 128.2, 44.3.

*4-Formylbenzonitrile* (**2k**) [27]. Following the general procedure with 4-(aminomethyl)benzonitrile (39.6 mg, 0.3 mmol), 2k was obtained as a white solid (27.5 mg, 70%). This target product was purified by column chromatography on silica gel (PE:EA = 10:1). ^1^H NMR (400 MHz, CDCl_3_) *δ* 10.09 (s, 1H), 7.99 (d, *J* = 8.0 Hz, 2H), 7.84 (d, *J* = 8.0 Hz, 2H). ^13^C NMR (101 MHz, CDCl_3_) *δ* 190.6, 138.7, 132.9, 129.9, 117.7, 117.6.

*4-Phenoxybenzaldehyde* (**2l**) [27]. Following the general procedure with (4-phenoxyphenyl)methanamine (59.7 mg, 0.3 mmol), **2l** was obtained as a white solid (23.8 mg, 40%). This target product was purified by column chromatography on silica gel (PE:EA = 10:1). ^1^H NMR (400 MHz, CDCl_3_) *δ* 9.92 (s, 1H), 7.91–7.79 (m, 2H), 7.48–7.36 (m, 2H), 7.28–7.18 (m, 1H), 7.13–7.02 (m, 4H). ^13^C NMR (101 MHz, CDCl_3_) *δ* 190.7, 163.2, 155.1, 131.9, 131.2, 130.1, 124.9, 120.4, 117.5.

*3-Methylbenzaldehyde* (**2m**) [27]. Following the general procedure with m-tolylmethanamine (36.3 mg, 0.3 mmol), **2m** was obtained as a colorless oil (19.8 mg, 55%). This target product was purified by column chromatography on silica gel (PE:EA = 10:1). ^1^H NMR (400 MHz, CDCl_3_) *δ* 9.98 (s, 1H), 7.71–7.62 (m, 2H), 7.47–7.36 (m, 2H), 2.43 (s, 3H). ^13^C NMR (101 MHz, CDCl_3_) *δ* 192.6, 138.9, 136.4, 135.2, 130.0, 128.8, 127.2, 21.1.

*3-Methoxybenzaldehyde* (**2n**) [27]. Following the general procedure with (3-methoxyphenyl)methanamine (41.1 mg, 0.3 mmol), **2n** was obtained as a colorless oil (20.4 mg, 50%). This target product was purified by column chromatography on silica gel (PE:EA = 10:1). ^1^H NMR (400 MHz, CDCl_3_) *δ* 9.97 (s, 1H), 7.50–7.42 (m, 2H), 7.41–7.36 (m, 1H), 7.21–7.14 (m, 1H), 3.86 (s, 3H). ^13^C NMR (101 MHz, CDCl_3_) *δ* 192.2, 160.2, 137.8, 130.1, 123.6, 121.5, 112.1, 55.5.

*3-Bromobenzaldehyde* (**2o**) [27]. Following the general procedure with (3-bromophenyl)methanamine (55.5 mg, 0.3 mmol), **2o** was obtained as a white solid (36.4 mg, 66%). Following the general procedure with (3–bromophenyl)methanamine 1-(3-bromophenyl)-*N*,*N*-dimethylmethanamine (64.2 mg, 0.3 mmol), **2o** was obtained as a white solid (23.9 mg, 55%). This target product was purified by column chromatography on silica gel (PE:EA = 10:1). ^1^H NMR (400 MHz, CDCl_3_) *δ* 9.95 (s, 1H), 8.00 (s, 1H), 7.80 (d, *J* = 7.6 Hz, 1H), 7.74 (d, *J* = 7.6 Hz, 1H), 7.42 (t, *J* = 8.0 Hz, 1H). ^13^C NMR (101 MHz, CDCl_3_) *δ* 190.7, 138.0, 137.3, 132.3, 130.6, 128.3, 123.3.

*3-Formylbenzonitrile* (**2p**) [53]. Following the general procedure with 3-(aminomethyl)benzonitrile (39.6 mg, 0.3 mmol), **2p** was obtained as a white solid (22.8 mg, 58%). This target product was purified by column chromatography on silica gel (PE:EA = 10:1). ^1^H NMR (400 MHz, CDCl_3_) *δ* 10.02 (s, 1H), 7.93–7.83 (m, 2H), 7.65–7.59 (m, 1H), 7.57–7.44 (m, 2H). ^13^C NMR (101 MHz, CDCl_3_) *δ* 190.1, 137.2, 136.9, 133.4, 133.1, 130.2, 117.6, 113.6.

*2-Methylbenzaldehyde* (**2q**) [27]. Following the general procedure with o-tolylmethanamine (36.3 mg, 0.3 mmol), **2q** was obtained as colorless oil (17.6 mg, 49%). This target product was purified by column chromatography on silica gel (PE:EA = 10:1). ^1^H NMR (400 MHz, CDCl_3_) *δ* 10.27 (d, *J* = 1.0 Hz, 1H), 7.83–7.76 (m, 1H), 7.51–7.44 (m, 1H), 7.36 (t, *J* = 7.6 Hz, 1H), 7.29–7.24 (m, 1H), 2.67 (s, 3H). ^13^C NMR (101 MHz, CDCl_3_) *δ* 192.8, 140.6, 134.1, 133.6, 132.0, 131.7, 126.3, 19.5.

*2-Chlorobenzaldehyde* (**2r**) [27]. Following the general procedure with (2-chlorophenyl)methanamine (42.3 mg, 0.3 mmol), **2r** was obtained as a white solid (24.8 mg, 59%). This target product was purified by column chromatography on silica gel (PE:EA = 10:1). ^1^H NMR (400 MHz, CDCl_3_) *δ* 10.49 (s, 1H), 7.92 (d, *J* = 8.0 Hz, 1H), 7.57–7.49 (m, 1H), 7.45 (d, *J* = 8.0 Hz, 1H), 7.39 (t, *J* = 7.2 Hz, 1H). ^13^C NMR (101 MHz, CDCl_3_) *δ* 189.8, 138.0, 135.1, 132.5, 130.6, 129.4, 127.3.

*2-Bromobenzaldehyde* (**2s**) [54]. Following the general procedure with (2-bromophenyl)methanamine (55.5 mg, 0.3 mmol), **2s** was obtained as a white solid (31.6 mg, 57%). This target product was purified by column chromatography on silica gel (PE:EA = 10:1). ^1^H NMR (400 MHz, CDCl_3_) *δ* 10.35 (s, 1H), 7.98–7.85 (m, 1H), 7.71–7.60 (m, 1H), 7.49–7.34 (m, 2H). ^13^C NMR (101 MHz, CDCl_3_) *δ* 191.8, 135.3, 133.9, 133.5, 129.8, 127.9, 127.1.

*3,4-Difluorobenzaldehyde* (**2t**) [27]. Following the general procedure with (3,4-difluorophenyl)methanamine (42.9 mg, 0.3 mmol), **2t** was obtained as a colorless oil (30.7 mg, 72%). This target product was purified by column chromatography on silica gel (PE:EA = 10:1). ^1^H NMR (400 MHz, CDCl_3_) *δ* 9.93 (s, 1H), 7.75–7.64 (m, 2H), 7.34 (q, *J* = 8.4 Hz, 1H). ^13^C NMR (101 MHz, CDCl_3_) *δ* 189.4 (d, *J* = 2.1 Hz), 154.5 (dd, *J* = 259.1, 13.0 Hz), 151.0 (dd, *J* = 252.7, 13.3 Hz), 133.5 (dd, *J* = 4.0, 3.7 Hz), 127.29 (dd, *J* = 7.8, 3.6 Hz), 118.15 (d, *J* = 18.2 Hz), 117.64 (dd, *J* = 17.6, 2.1 Hz). ^19^F NMR (376 MHz, CDCl_3_) *δ* –126.89 (d, *J* = 20.4 Hz), –135.21 (d, *J* = 20.4 Hz).

*3,4-Dimethylbenzaldehyde* (**2u**) [27]. Following the general procedure with (3,4-dimethylphenyl)methanamine (40.5 mg, 0.3 mmol), **2u** was obtained as a colorless oil (17.7 mg, 44%). This target product was purified by column chromatography on silica gel (PE:EA = 10:1). ^1^H NMR (400 MHz, CDCl_3_) *δ* 9.93 (s, 1H), 7.64 (s, 1H), 7.61 (d, *J* = 8.0 Hz, 1H), 7.28 (d, *J* = 8.0 Hz, 1H), 2.33 (d, *J* = 2.0 Hz, 6H). ^13^C NMR (101 MHz, CDCl_3_) *δ* 192.2, 144.3, 137.5, 134.6, 130.5, 130.2, 127.7, 20.2, 19.6.

*Acetophenone* (**2v**) [27]. Following the general procedure with 1-phenylethan-1-amine (36.3 mg, 0.3 mmol), **2v** was obtained as a colorless oil (23.8 mg, 66%). This target product was purified by column chromatography on silica gel (PE:EA = 10:1). ^1^H NMR (400 MHz, CDCl_3_) *δ* 7.96 (d, *J* = 8.0 Hz, 2H), 7.56 (t, *J* = 7.2 Hz, 1H), 7.46 (t, *J* = 7.2 Hz, 2H), 2.61 (s, 3H). ^13^C NMR (101 MHz, CDCl_3_) *δ* 198.1, 137.1, 133.1, 128.5, 128.3, 26.6.

*Benzophenone* (**2w**) [27]. Following the general procedure with diphenylmethanamine (54.9 mg, 0.3 mmol), **2w** was obtained as a white solid (27.8 mg, 51%). This target product was purified by column chromatography on silica gel (PE:EA = 10:1). ^1^H NMR (400 MHz, CDCl_3_) *δ* 7.81 (d, *J* = 8.0 Hz, 4H), 7.59 (t, *J* = 7.6 Hz, 2H), 7.48 (t, *J* = 7.6 Hz, 4H). ^13^C NMR (101 MHz, CDCl_3_) *δ* 196.7, 137.6, 132.4, 130.0, 128.2.

*3-Nitrobenzaldehyde* (**2x**) [55]. Following the general procedure with *N*-methyl-1-(3-nitrophenyl)methanamine (49.8 mg, 0.3 mmol), **2x** was obtained as a white solid (18.9 mg, 38%). This target product was purified by column chromatography on silica gel (PE:EA = 10:1). ^1^H NMR (400 MHz, CDCl_3_) *δ* 10.13 (s, 1H), 8.75–8.70 (m, 1H), 8.50 (d, *J* = 7.6 Hz, 1H), 8.24 (d, *J* = 7.6 Hz, 1H), 7.81–7.74 (m, 1H). ^13^C NMR (101 MHz, CDCl_3_) *δ* 189.7, 137.4, 134.6, 130.4, 128.6, 124.5.

## 4. Conclusions

In conclusion, we have developed an efficient electrochemical method for the oxidative cleavage of C–N bonds under mild conditions using water as the oxygen source. Unlike traditional transition-metal-catalyzed methods for breaking C–N bonds, this approach offers gentle reaction conditions without requiring catalysts or oxidants. It presents a novel approach for the cleavage and conversion of C–N bonds, with the potential to pave the way for the design and improvement of synthetic pathways.

## Figures and Tables

**Table 1:** Optimization of the reaction conditions *^a^.*

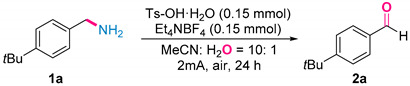		
Entry	Variations from the Standard Conditions	Yield
1	None	79%
2	without current	N.R.
3	without TsOH·H_2_O	35%
4	TFA instead of TsOH·H_2_O	43%
5	AcOH instead of TsOH·H_2_O	40%
6	PA instead of TsOH·H_2_O	39%
7	C/C instead of Pt/Pt	44%
8	4 mA instead of 2 mA	77%

*^a^* Standard conditions: **1a** (0.3 mmol, 1.0 equiv.), TsOH·H_2_O (0.15 mmol, 0.5 equiv.), Et_4_NBF_4_ (0.15 mmol, 0.5 equiv.), and MeCN/H_2_O = (2.0 mL/0.2 mL); constant current = 2.0 mA in air for 24 h. Isolated yields. N.R. = no reaction. TFA: trifluoroacetic acid. AcOH: acetic acid. PA: pivalic acid.

## Data Availability

The data presented in this study are available in the article and Supplementary Material.

## Supplementary Materials

The following supporting information can be downloaded at https://www.mdpi.com/article/10.3390/molecules29122851/s1. Copies of ^1^H-NMR, ^13^C-NMR, and ^19^F-NMR spectra of the products are included in the Supporting Information.
