# Supplementary material for: Selective Oxidative Cleavage of Benzyl C–N Bond under Metal-Free Electrochemical Conditions
Source: Molecules. 2024 Jun 15;29(12):2851. doi: 10.3390/molecules29122851 (PMC11206264; doi:10.3390/molecules29122851)

Supporting Information

Selective oxidative cleavage of benzyl C–N bond under metal-free electrochemical conditions

Jiawei Huang [1,‡], Xiaoman Li [1,‡], Ping Liu [1], Yu Wei [1], Shuai Liu [2,*], Xiaowei Ma [1,*]

[1] School of Chemistry and Chemical Engineering/State Key Laboratory Incubation Base for Green Processing of Chemical Engineering, Shihezi University, Shihezi, China.

[2] Bingtuan Energy Development Institute, Shihezi University, Shihezi, 832003, China.

Contents

Copies of NMR spectra..3

Copies of NMR spectra

^{1}H NMR spectra of compound **2a** (400 MHz, $CDCl_3$)

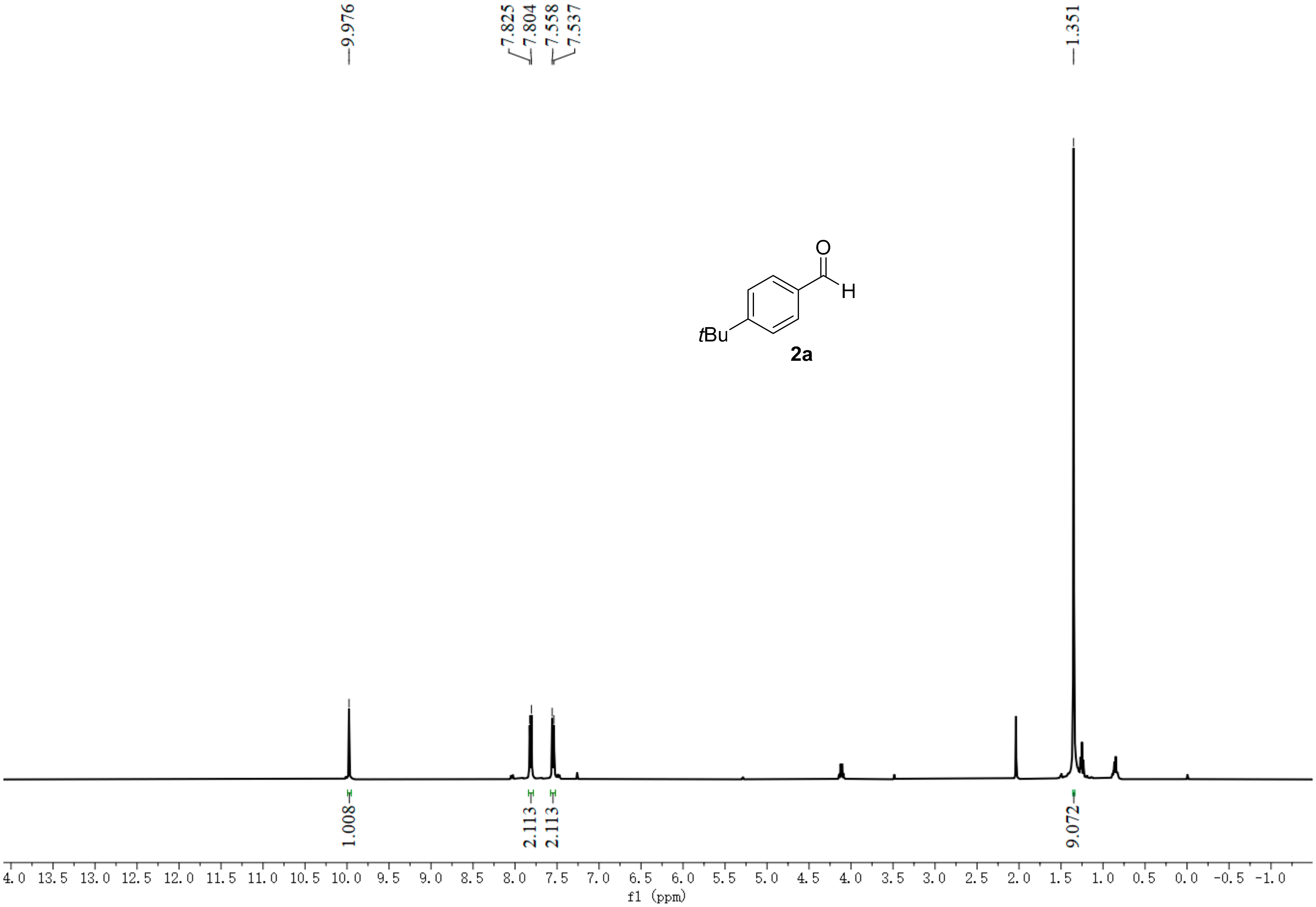

^{13}C NMR spectra of compound **2a** (101 MHz, $CDCl_3$)

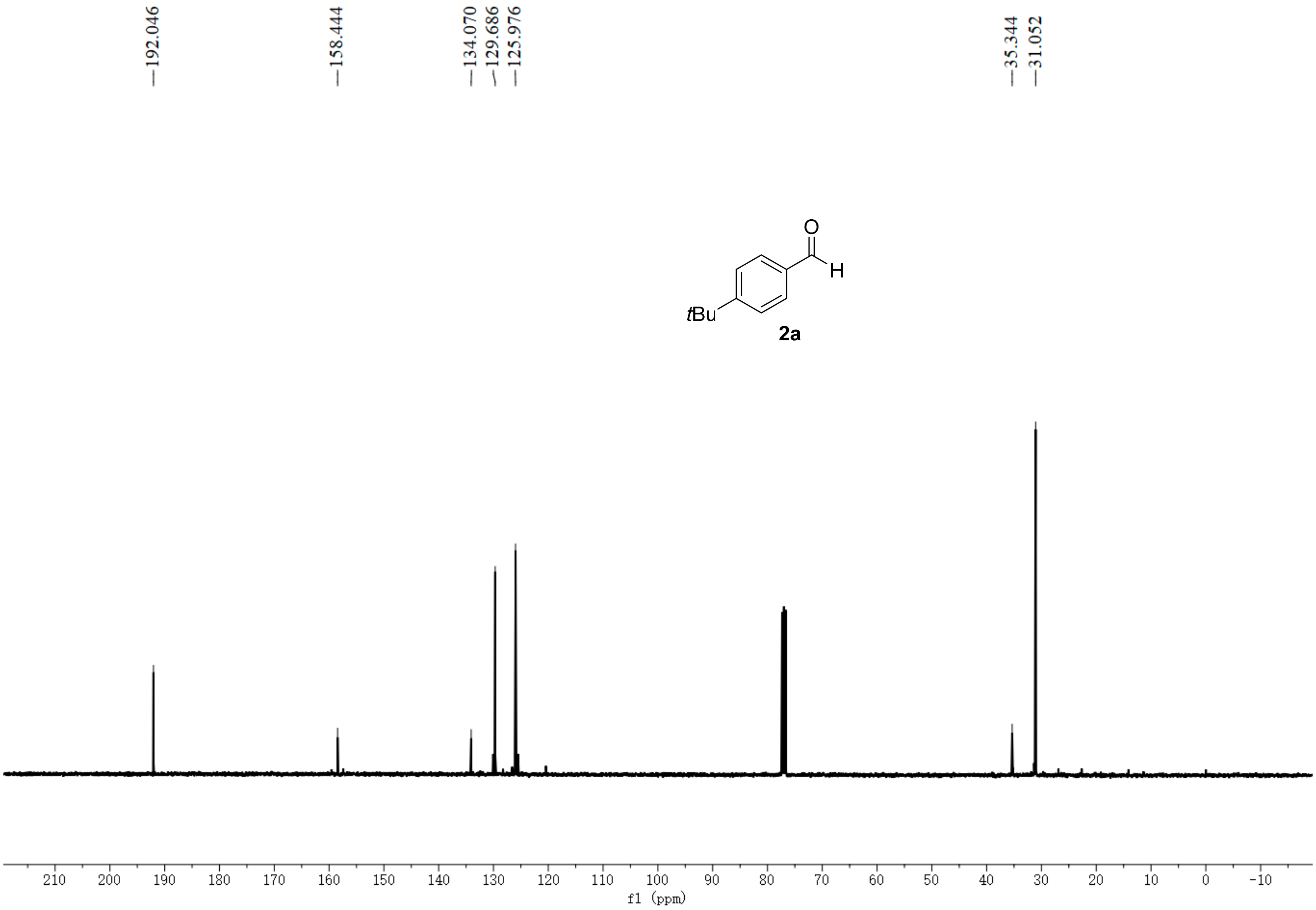

^{1}H NMR spectra of compound **2b** (400 MHz, $CDCl_3$)

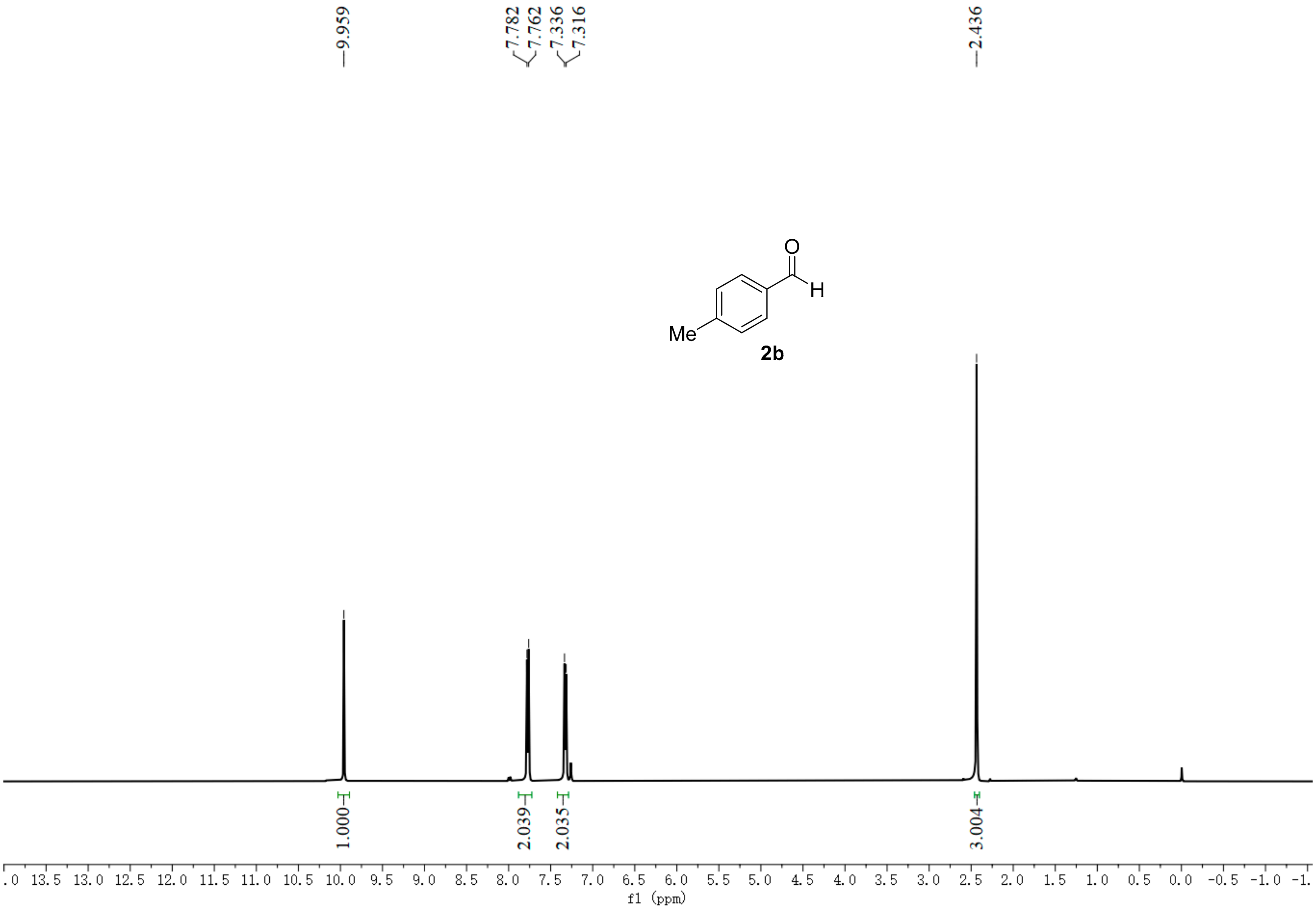

^{13}C NMR spectra of compound **2b** (101 MHz, $CDCl_3$)

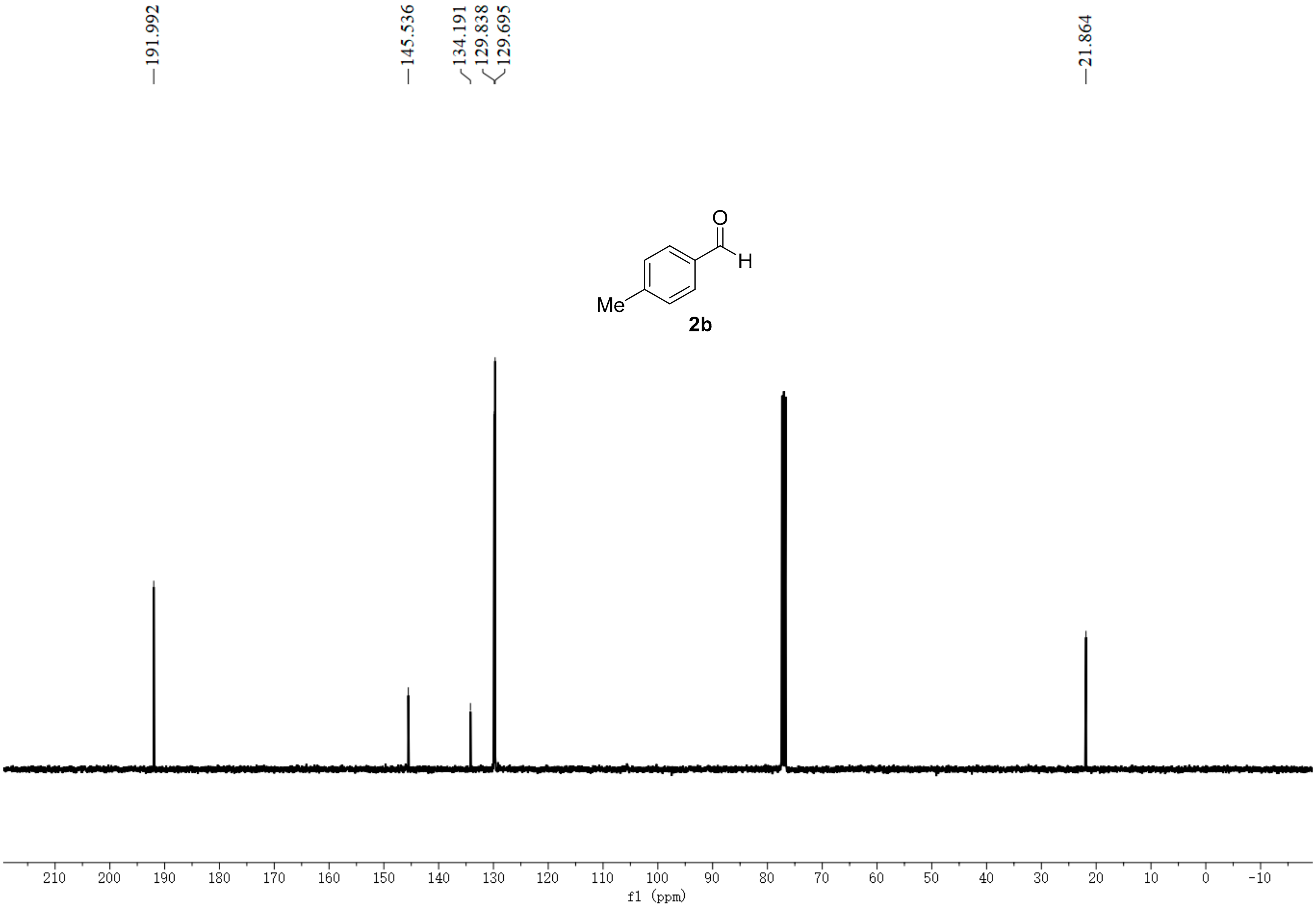

^{1}H NMR spectra of compound **2c** (400 MHz, $CDCl_3$)

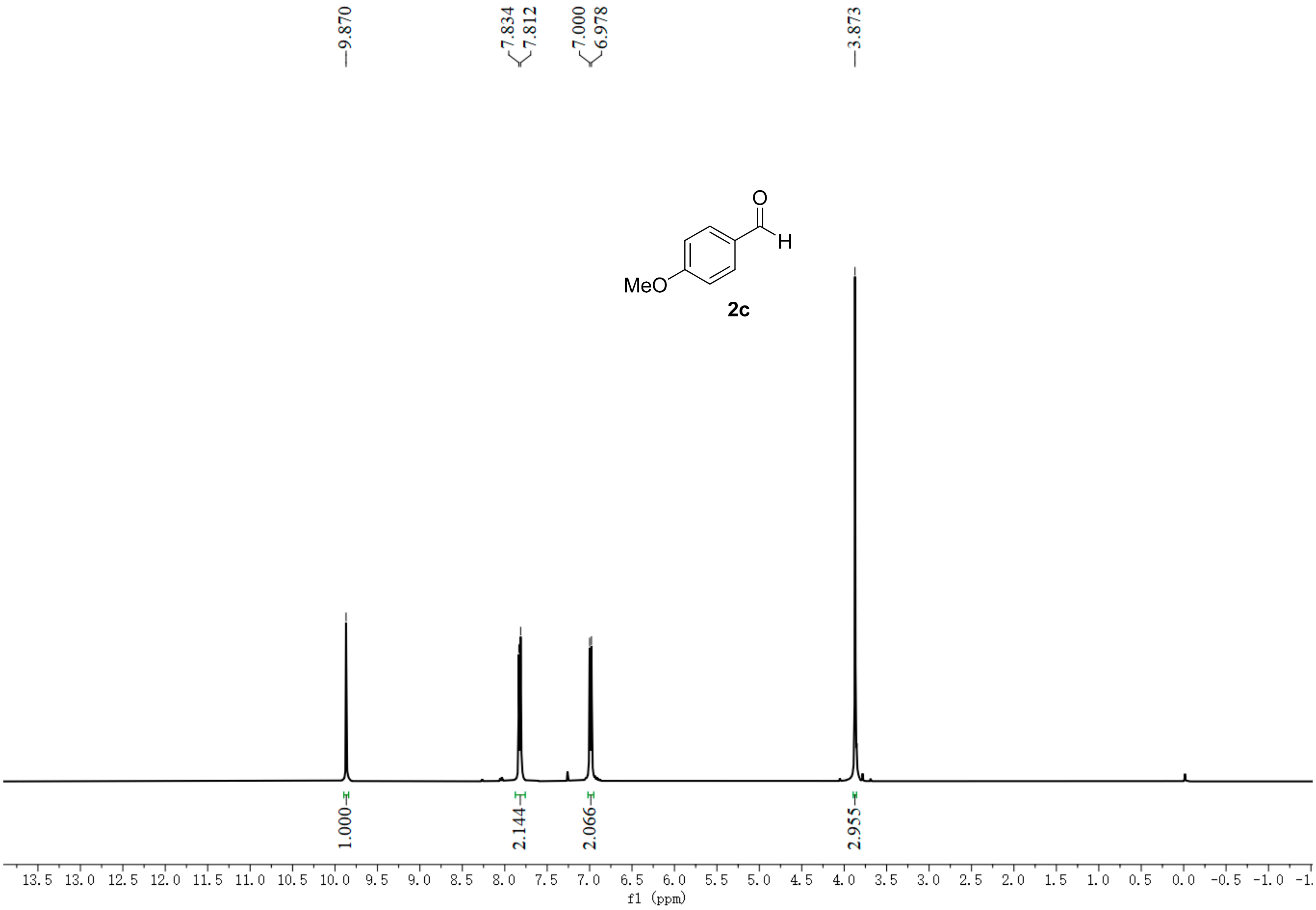

^{13}C NMR spectra of compound **2c** (101 MHz, $CDCl_3$)

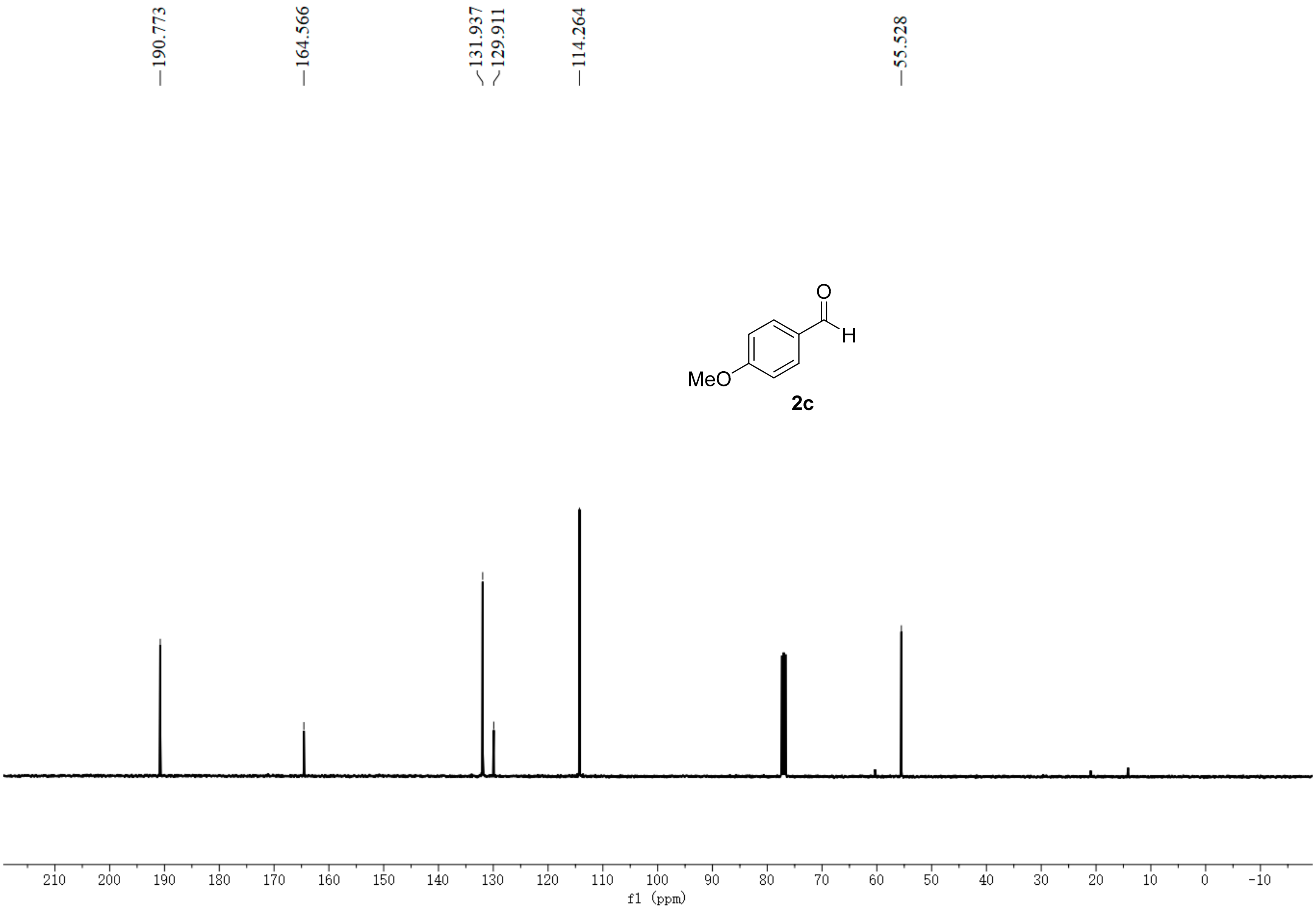

^{1}H NMR spectra of compound **2d** (400 MHz, $CDCl_3$)

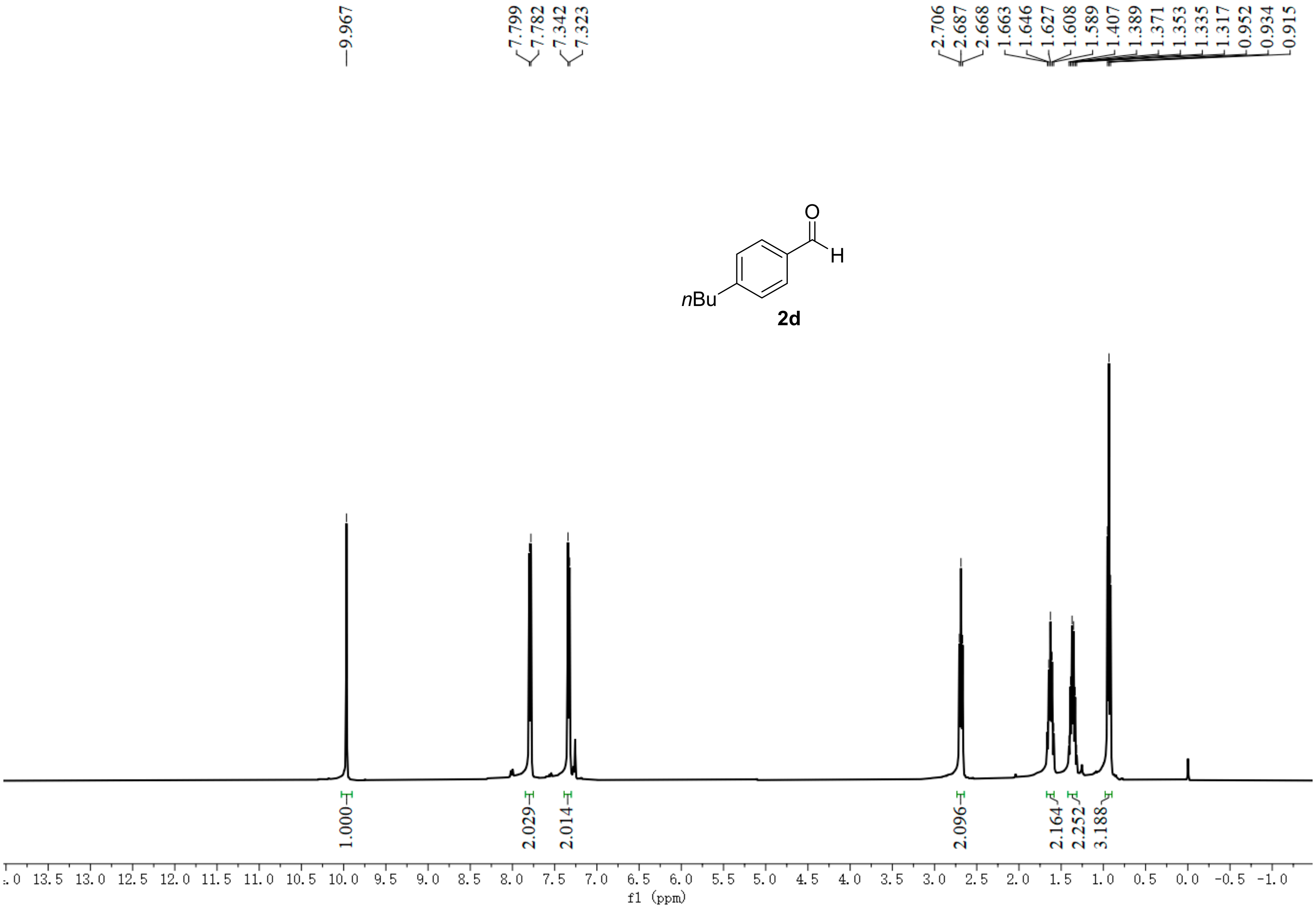

^{13}C NMR spectra of compound **2d** (101 MHz, $CDCl_3$)

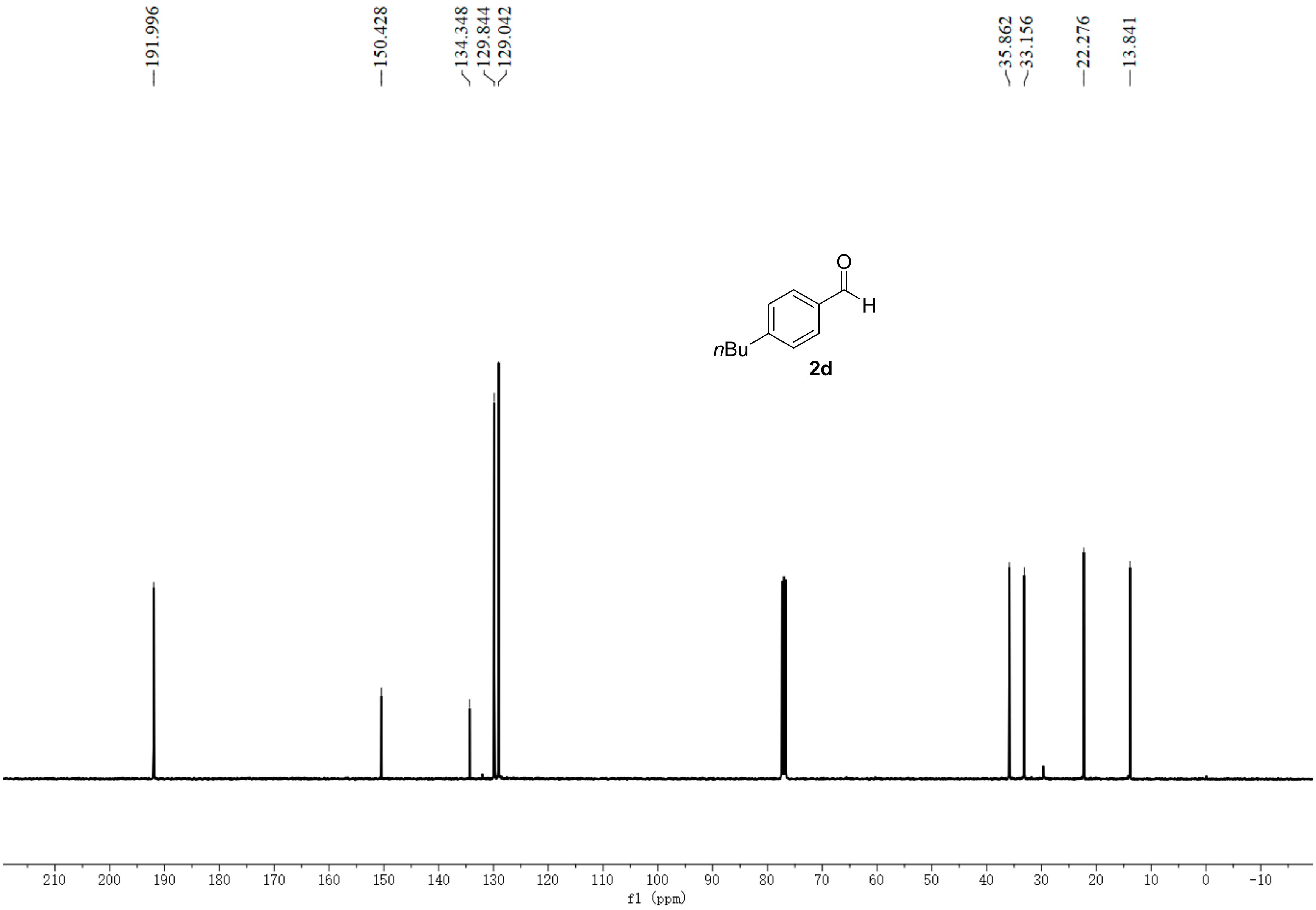

^1H NMR spectra of compound **2e** (400 MHz, $CDCl_3$)

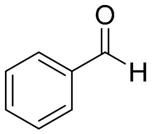

2e

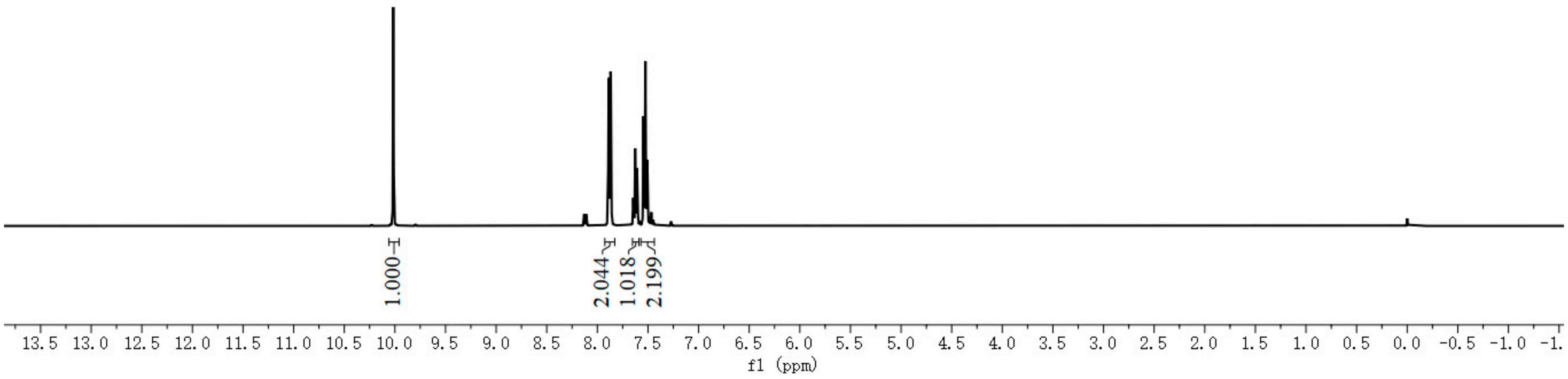

^{13}C NMR spectra of compound **2e** (101 MHz, $CDCl_3$)

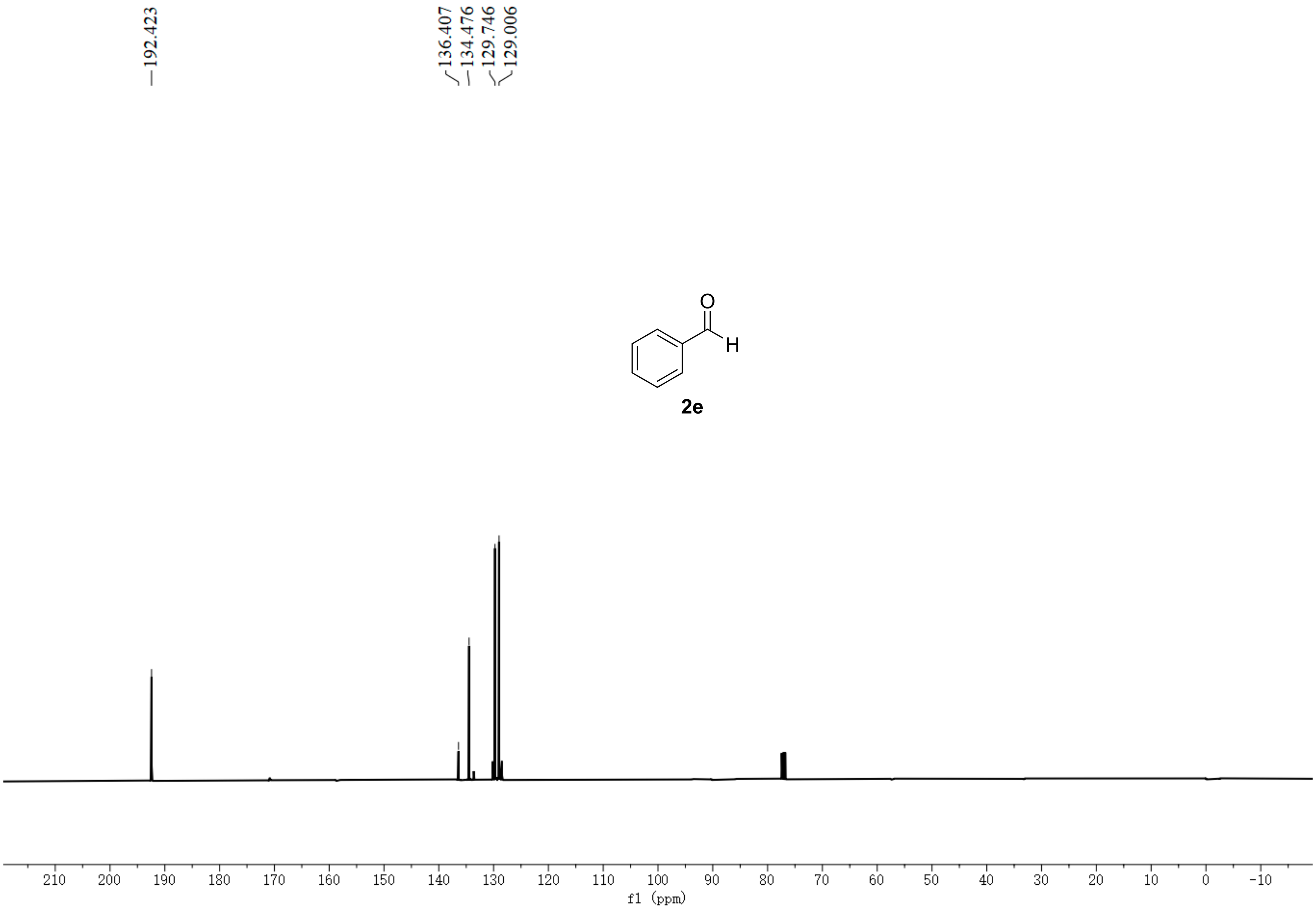

^{1}H NMR spectra of compound **2f** (400 MHz, $CDCl_3$)

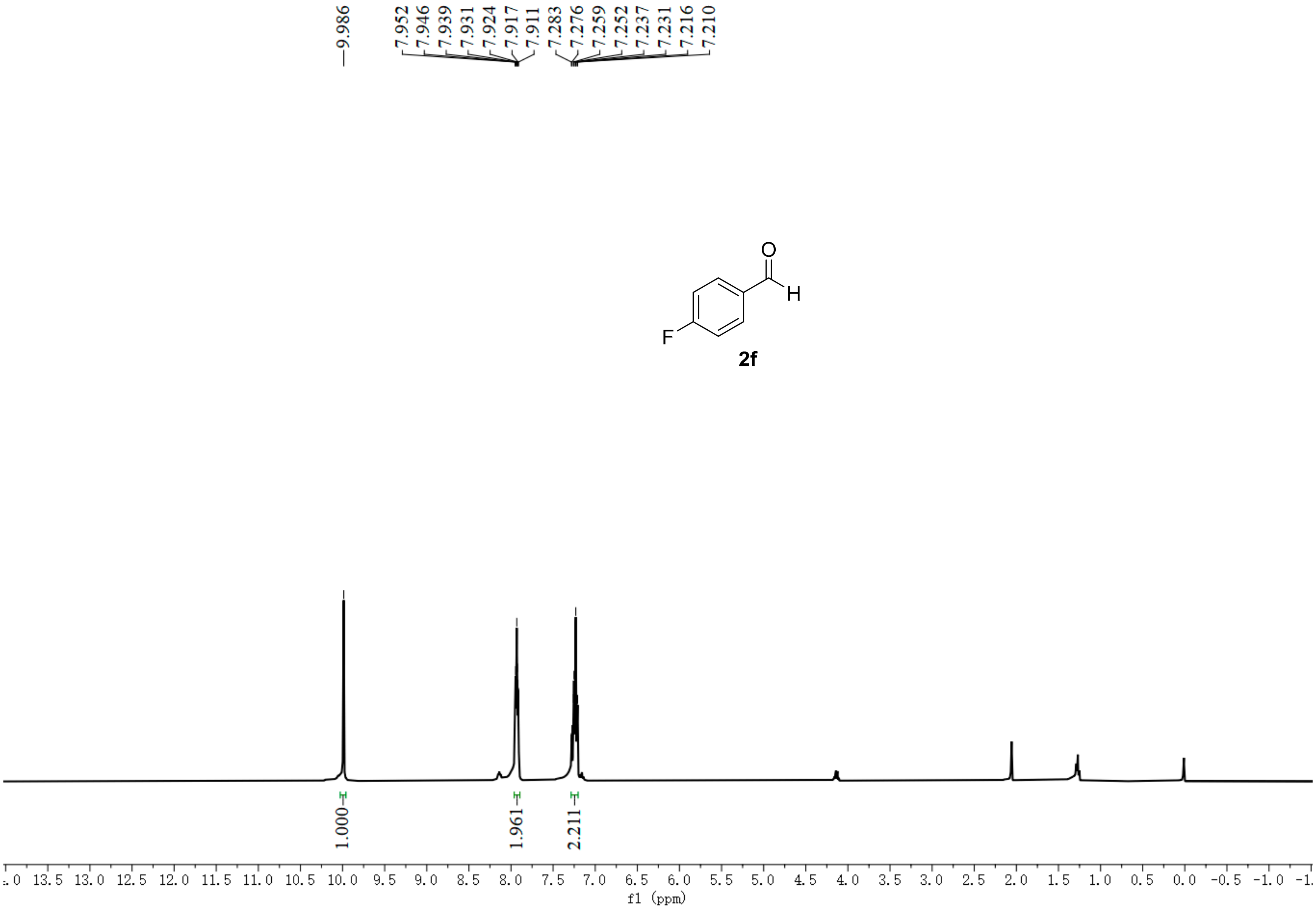

^{13}C NMR spectra of compound **2f** (101 MHz, $CDCl_3$)

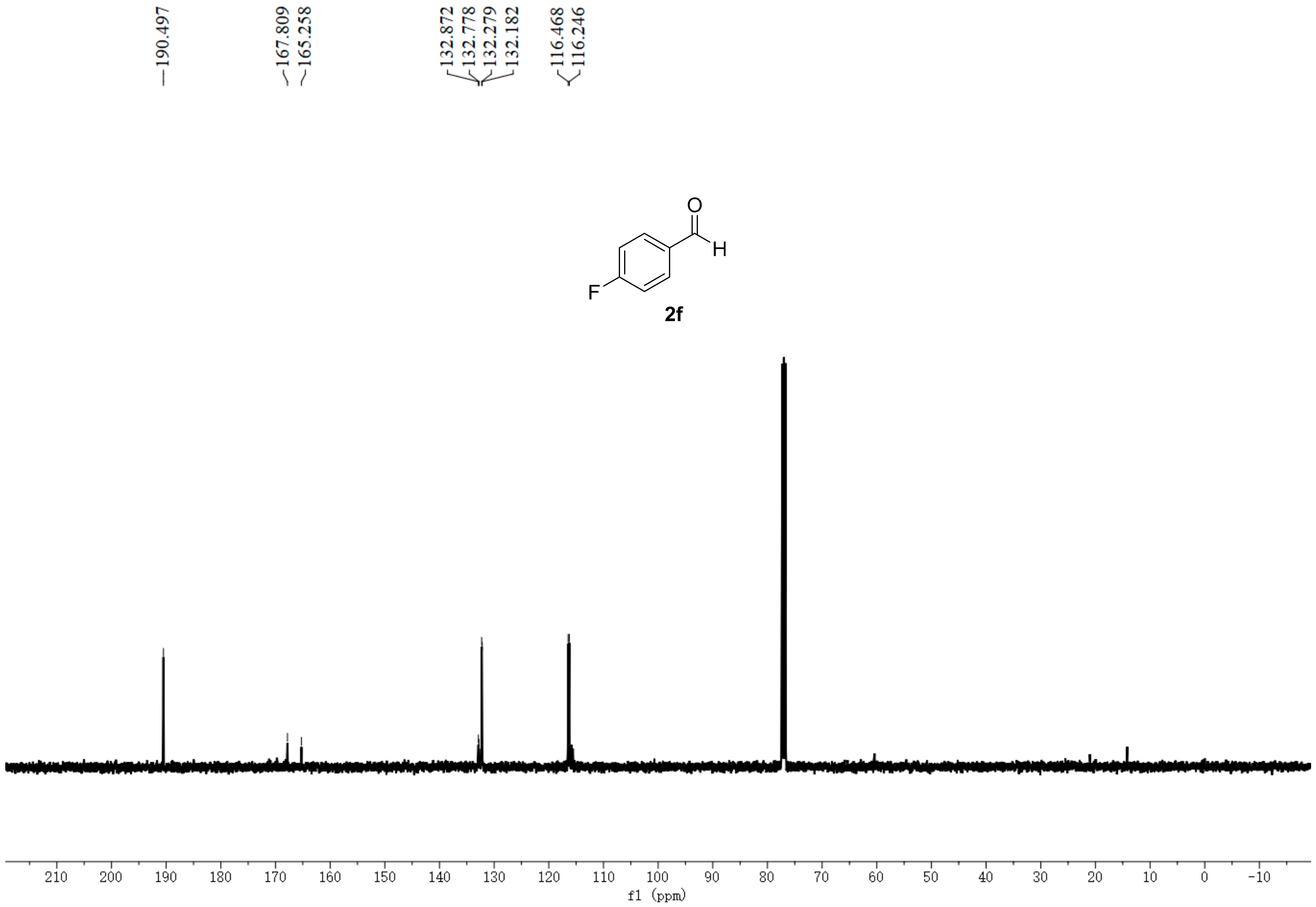

^{19}F NMR spectra of compound **2f** (376 MHz, $CDCl_3$)

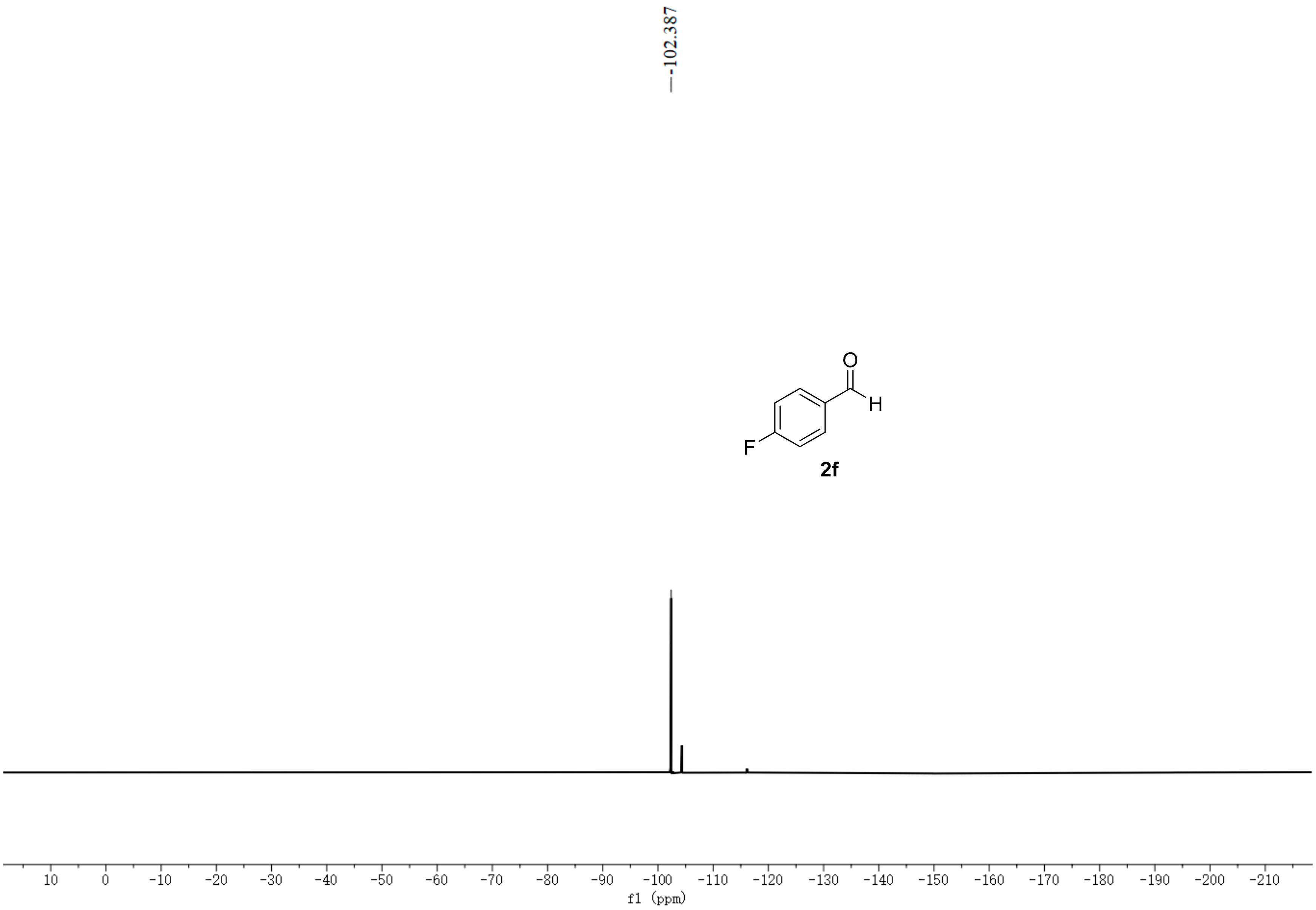

^{1}H NMR spectra of compound **2g** (400 MHz, $CDCl_3$)

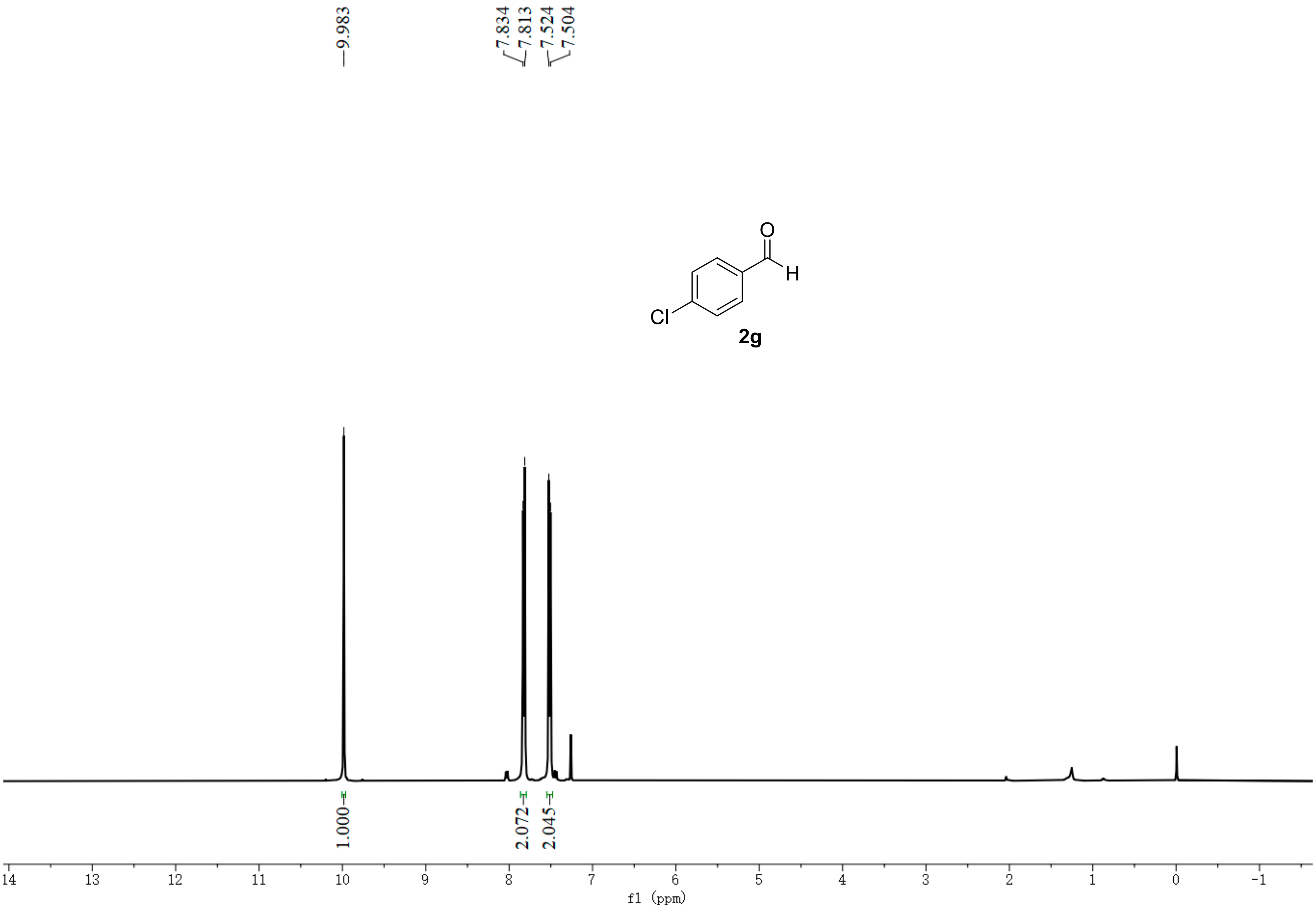

2g

^{13}C NMR spectra of compound **2g** (101 MHz, $CDCl_3$)

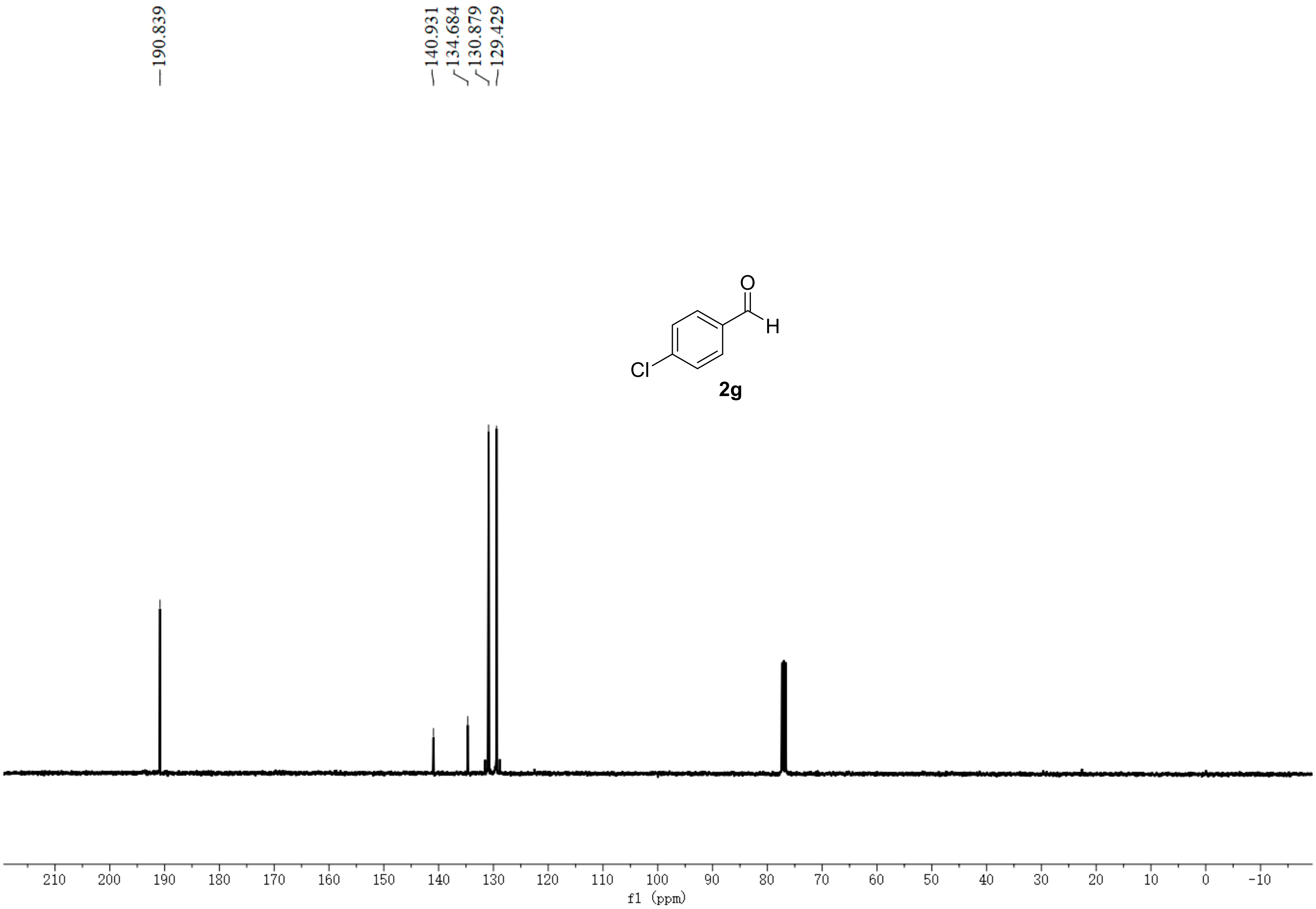

^{1}H NMR spectra of compound **2h** (400 MHz, $CDCl_3$)

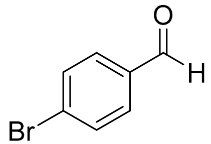

2h

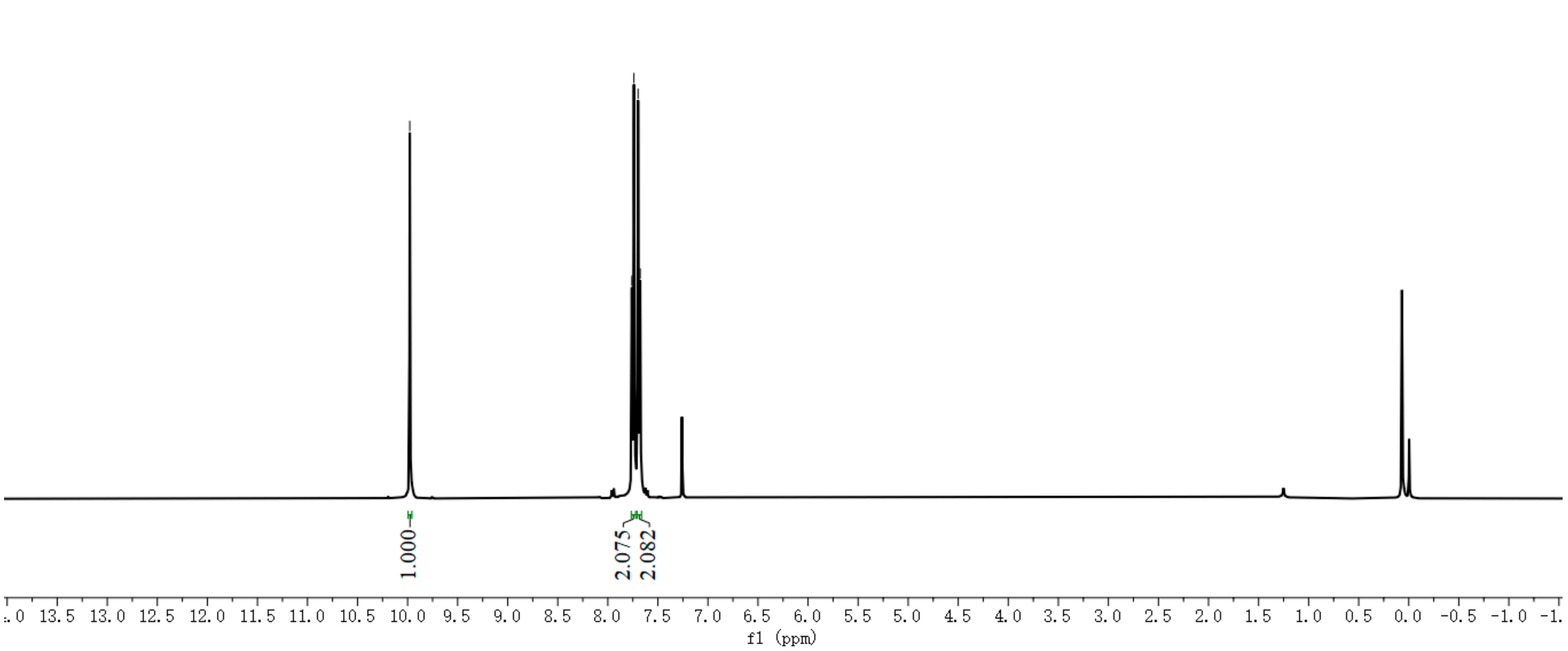

^{13}C NMR spectra of compound **2h** (101 MHz, $CDCl_3$)

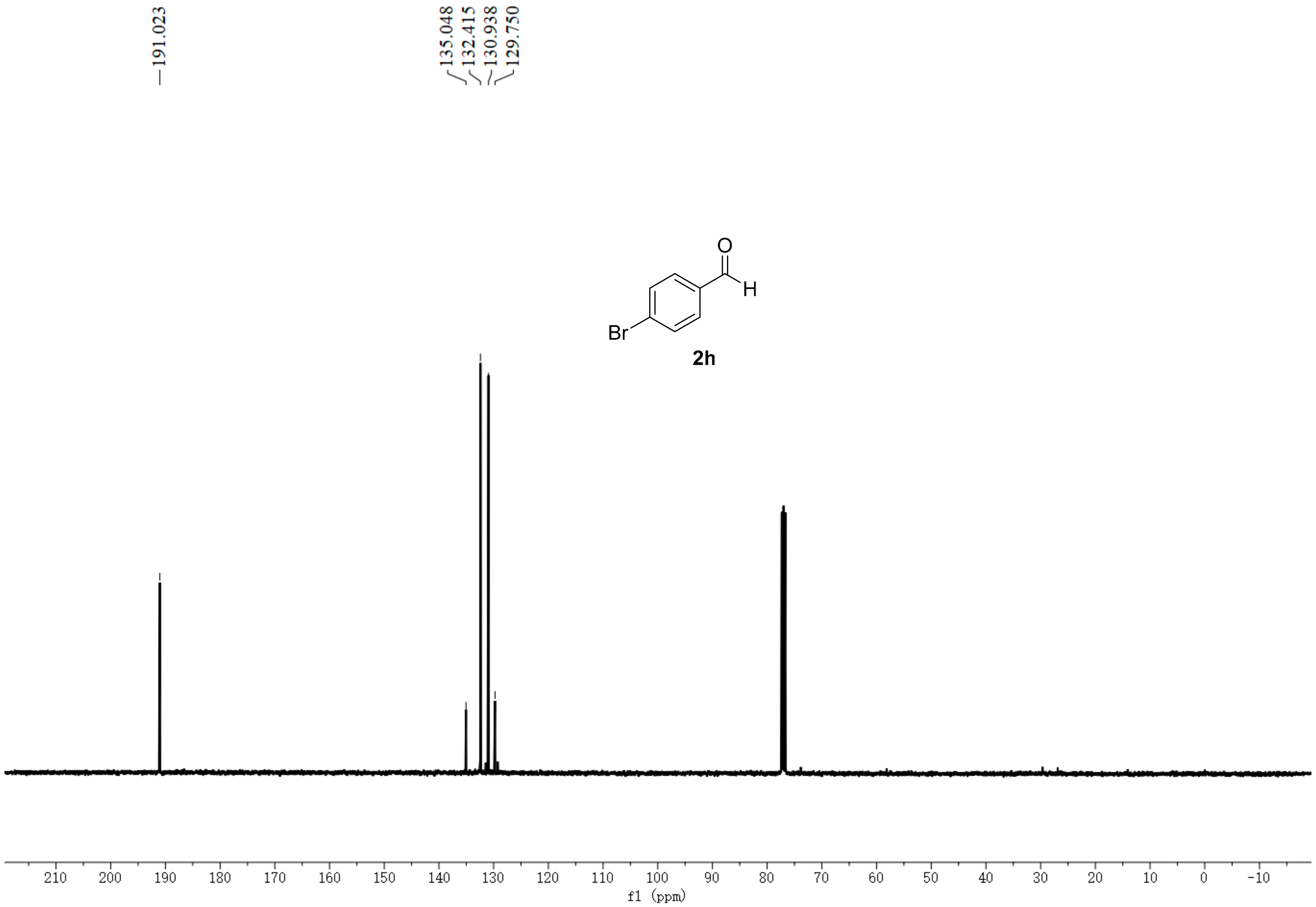

^{1}H NMR spectra of compound **2i** (400 MHz, $CDCl_3$)

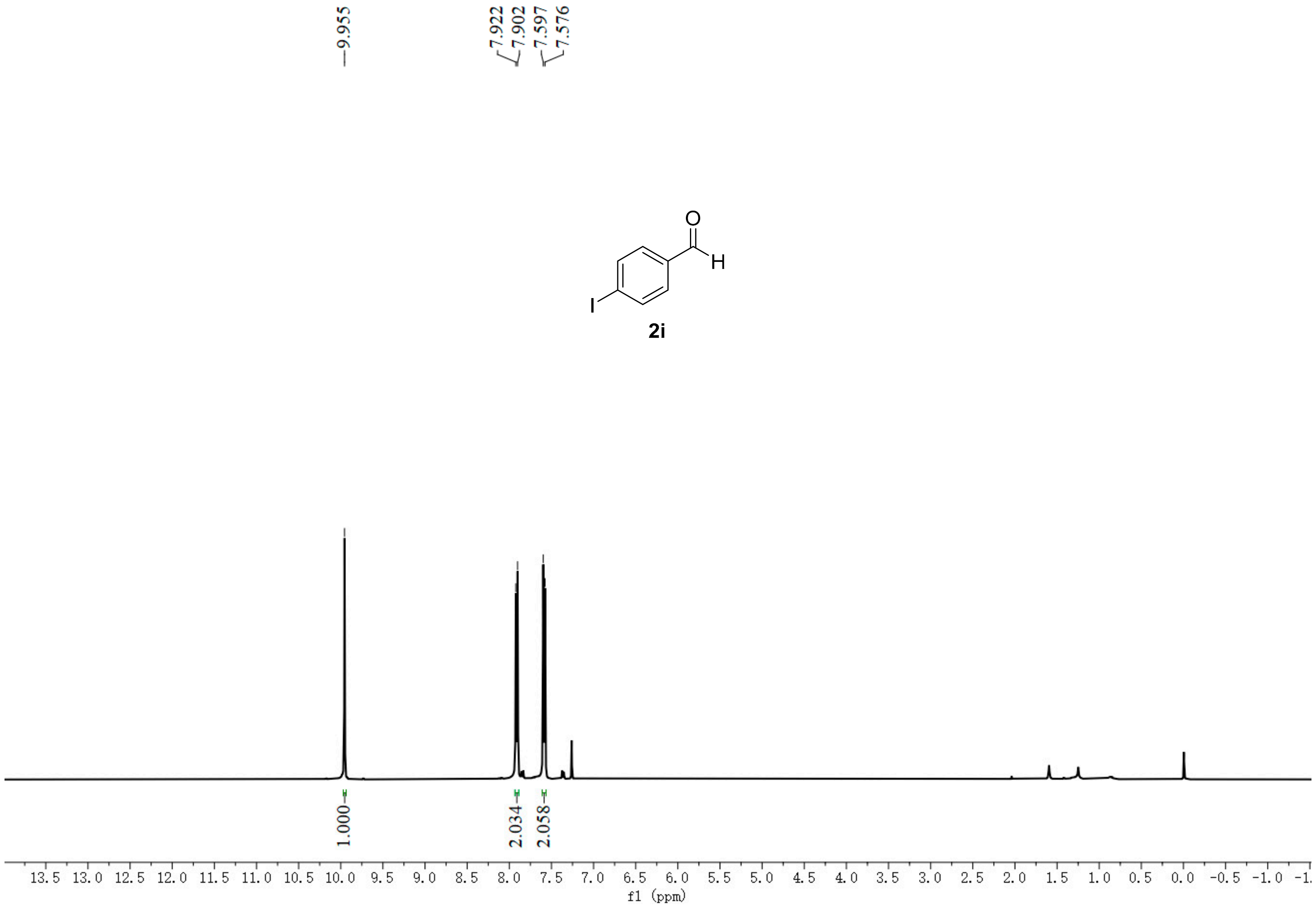

^{13}C NMR spectra of compound **2i** (101 MHz, $CDCl_3$)

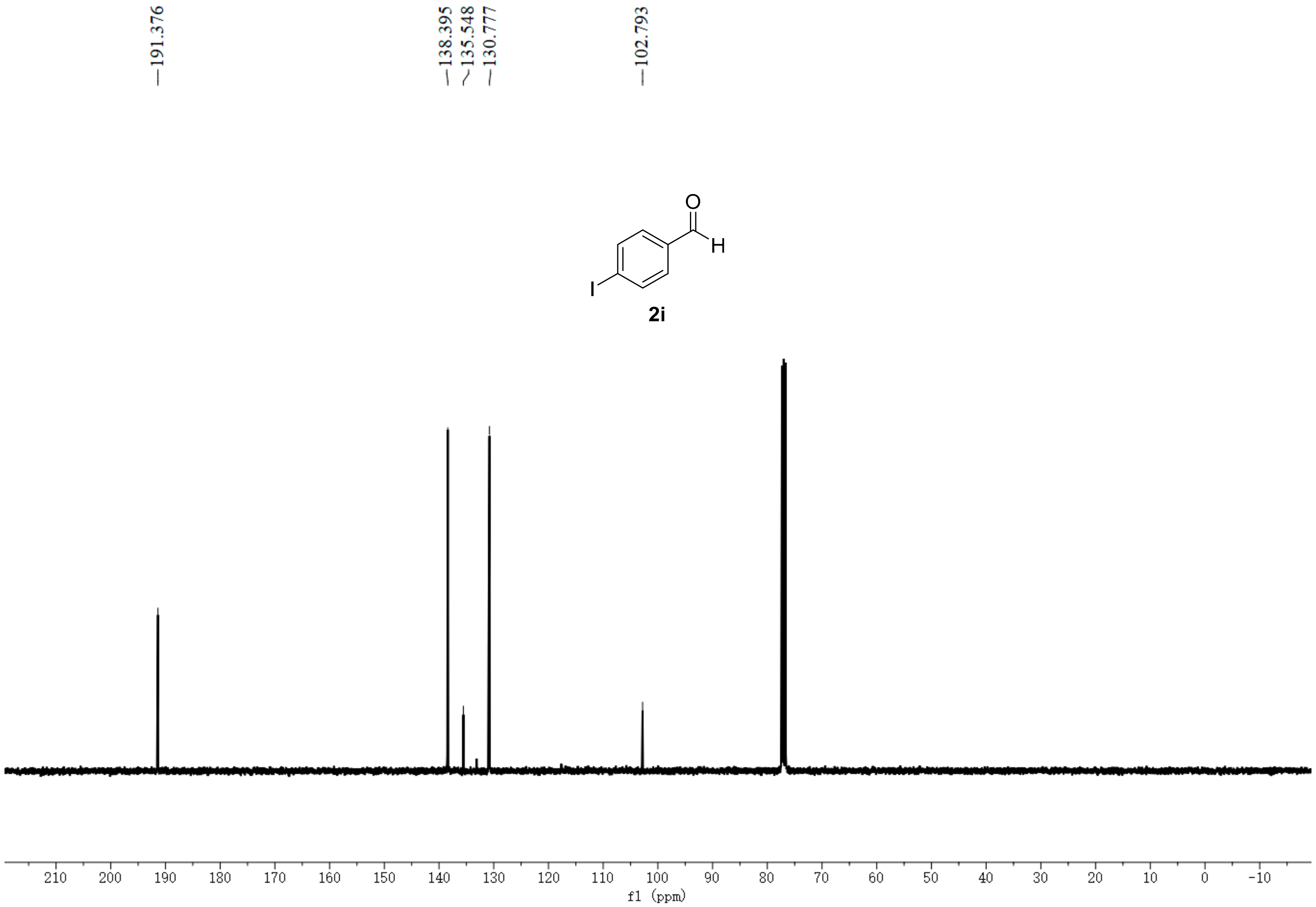

^{1}H NMR spectra of compound **2j** (400 MHz, $CDCl_3$)

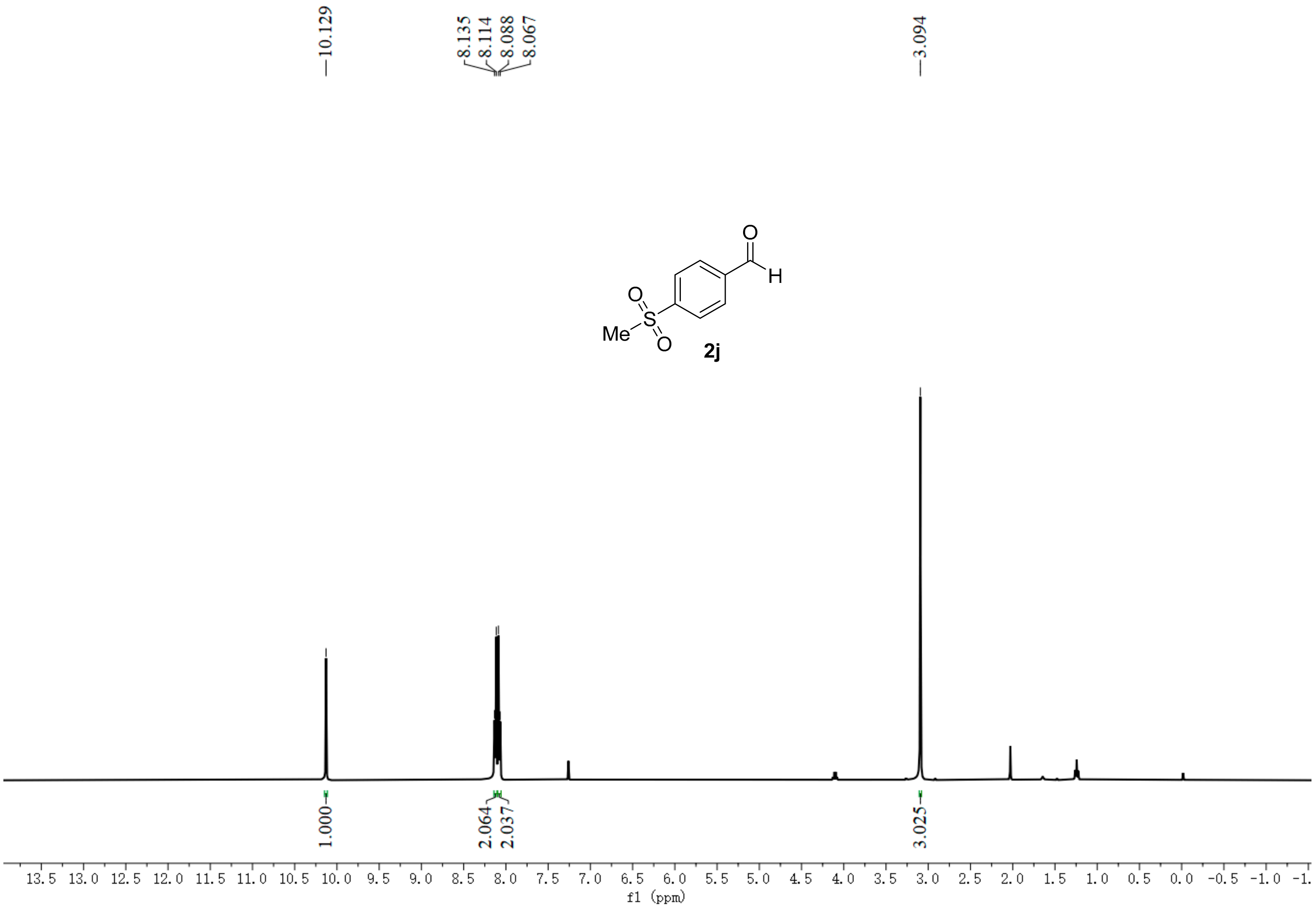

^{13}C NMR spectra of compound **2j** (101 MHz, $CDCl_3$)

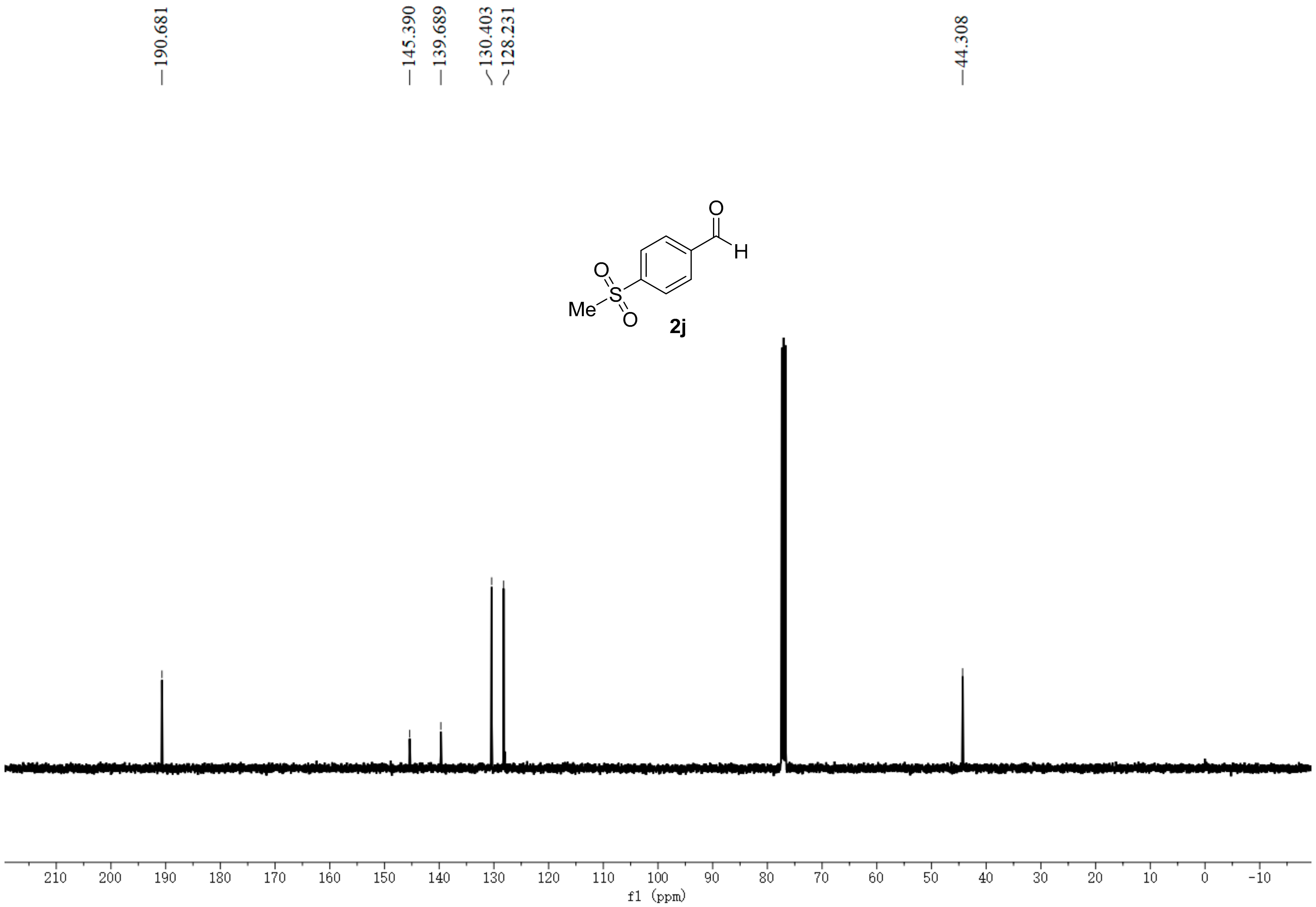

^{1}H NMR spectra of compound **2k** (400 MHz, $CDCl_3$)

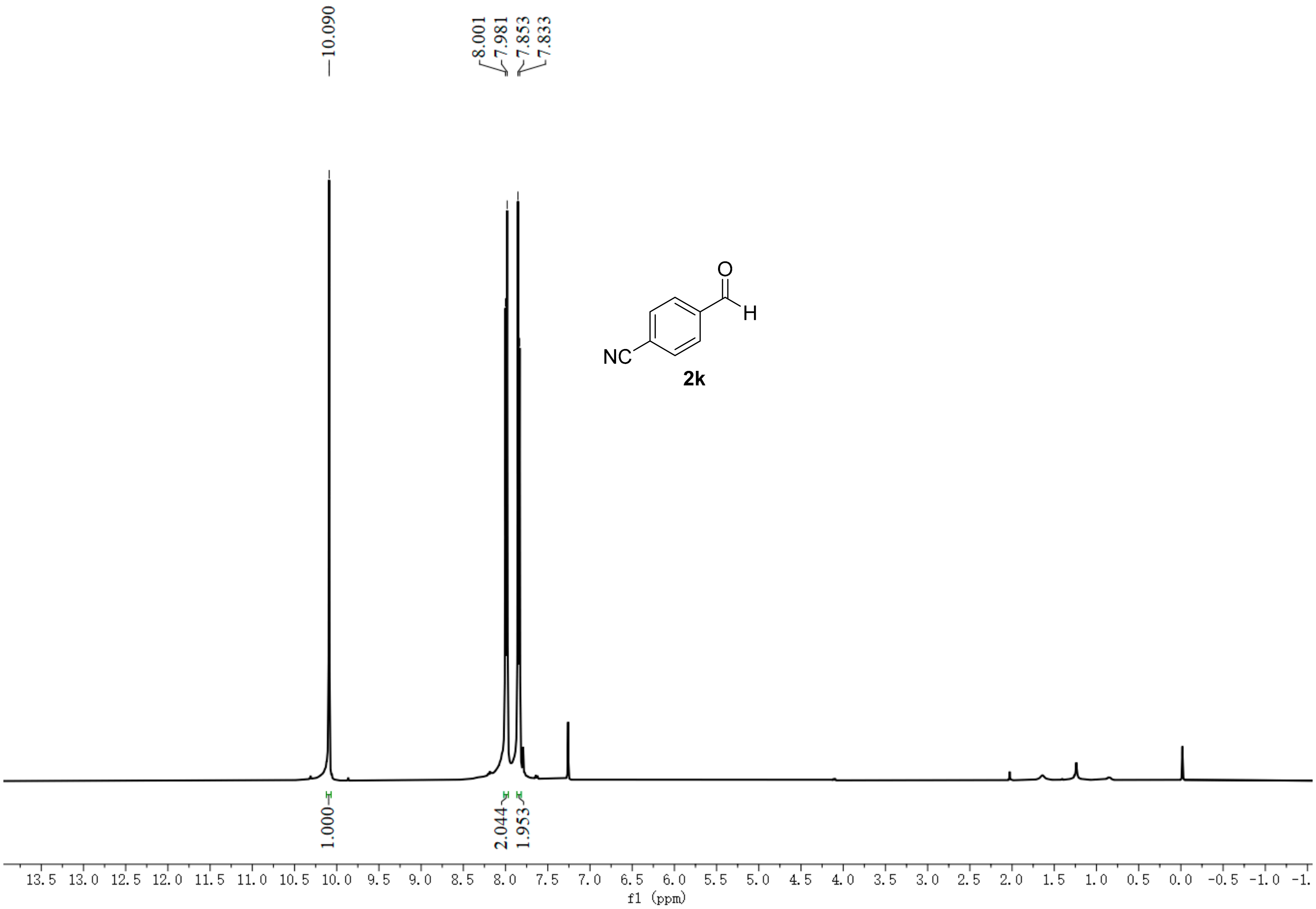

^{13}C NMR spectra of compound **2k** (101 MHz, $CDCl_3$)

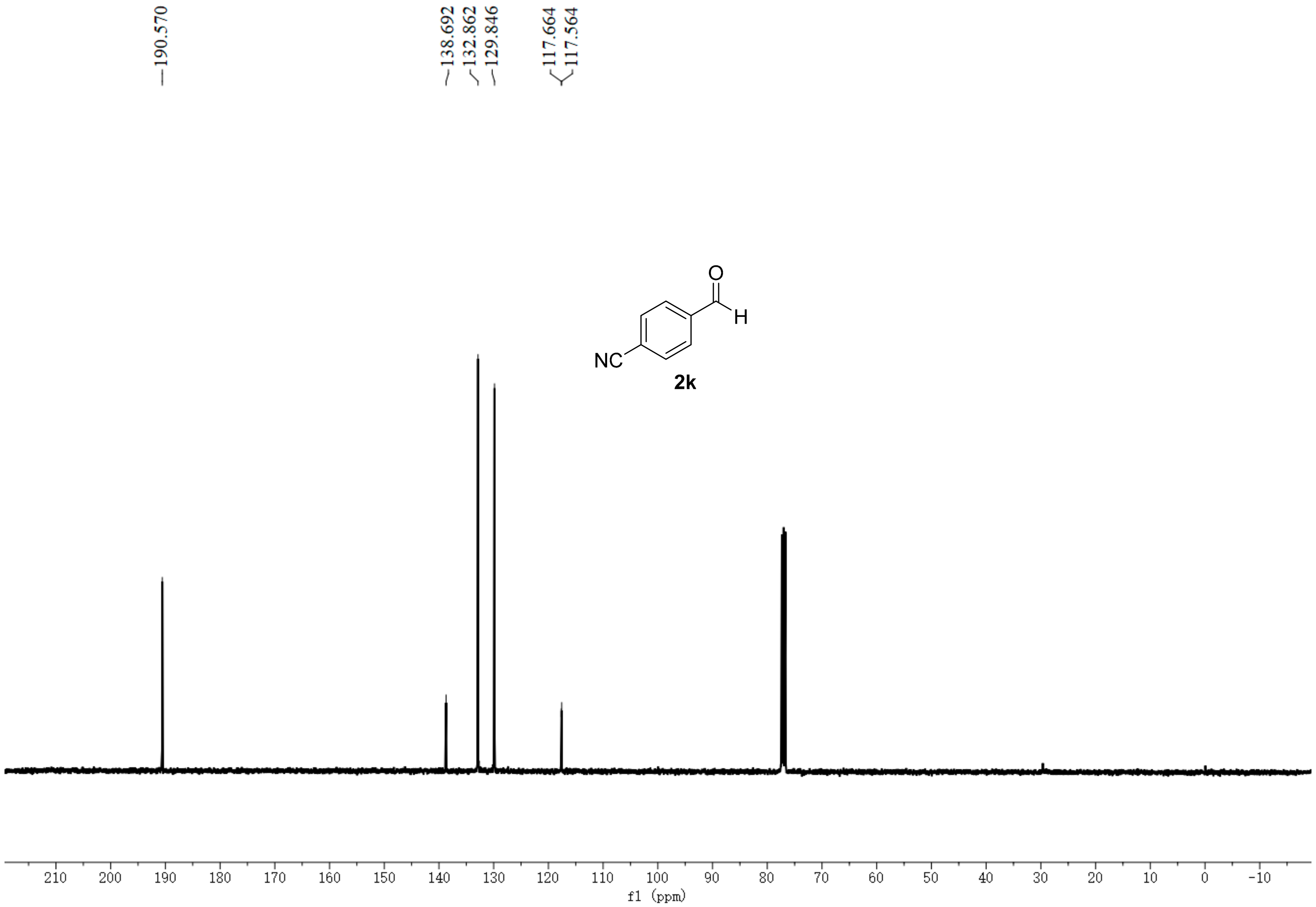

^1H NMR spectra of compound **2l** (400 MHz, $CDCl_3$)

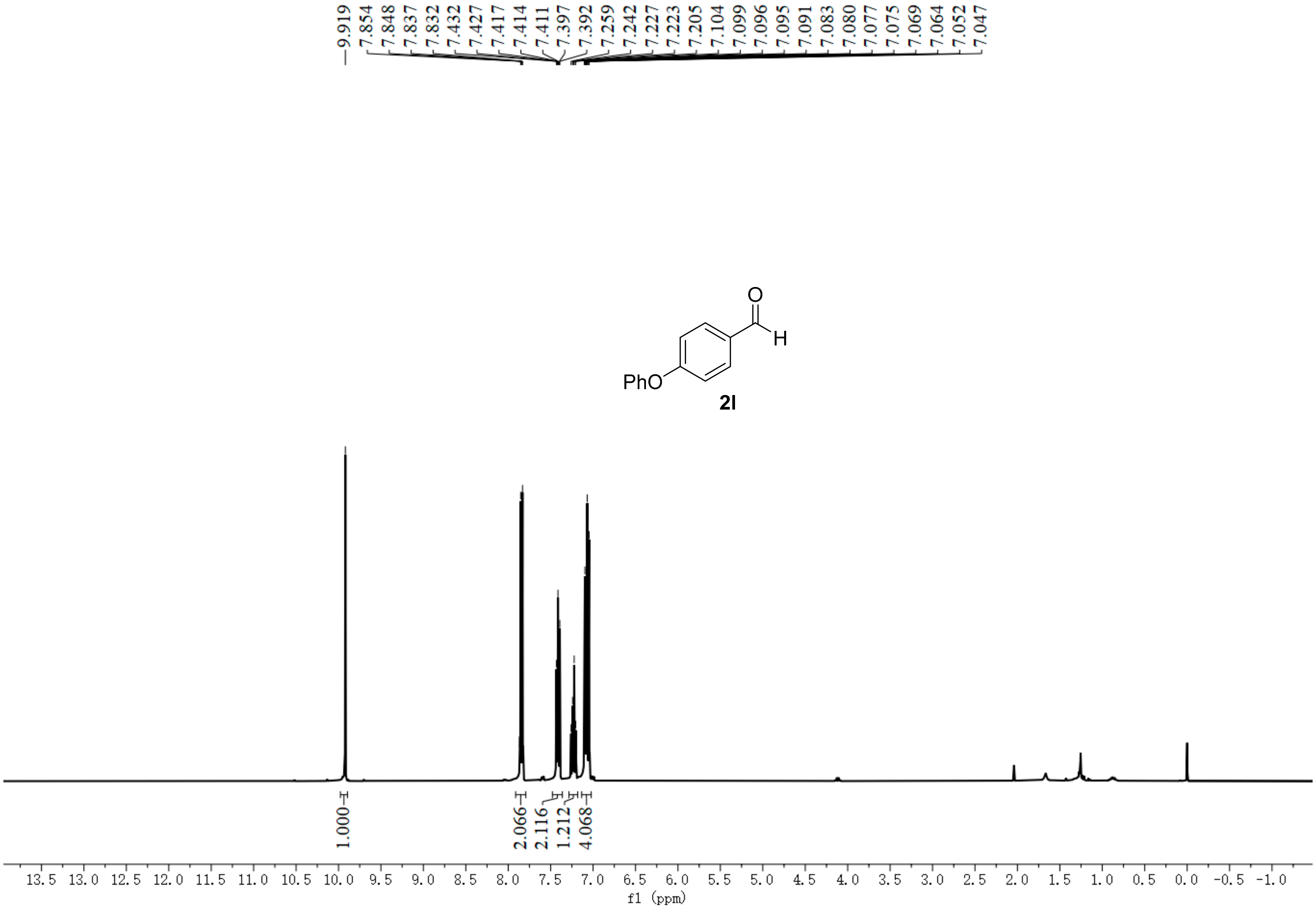

^{13}C NMR spectra of compound **2l** (101 MHz, $CDCl_3$)

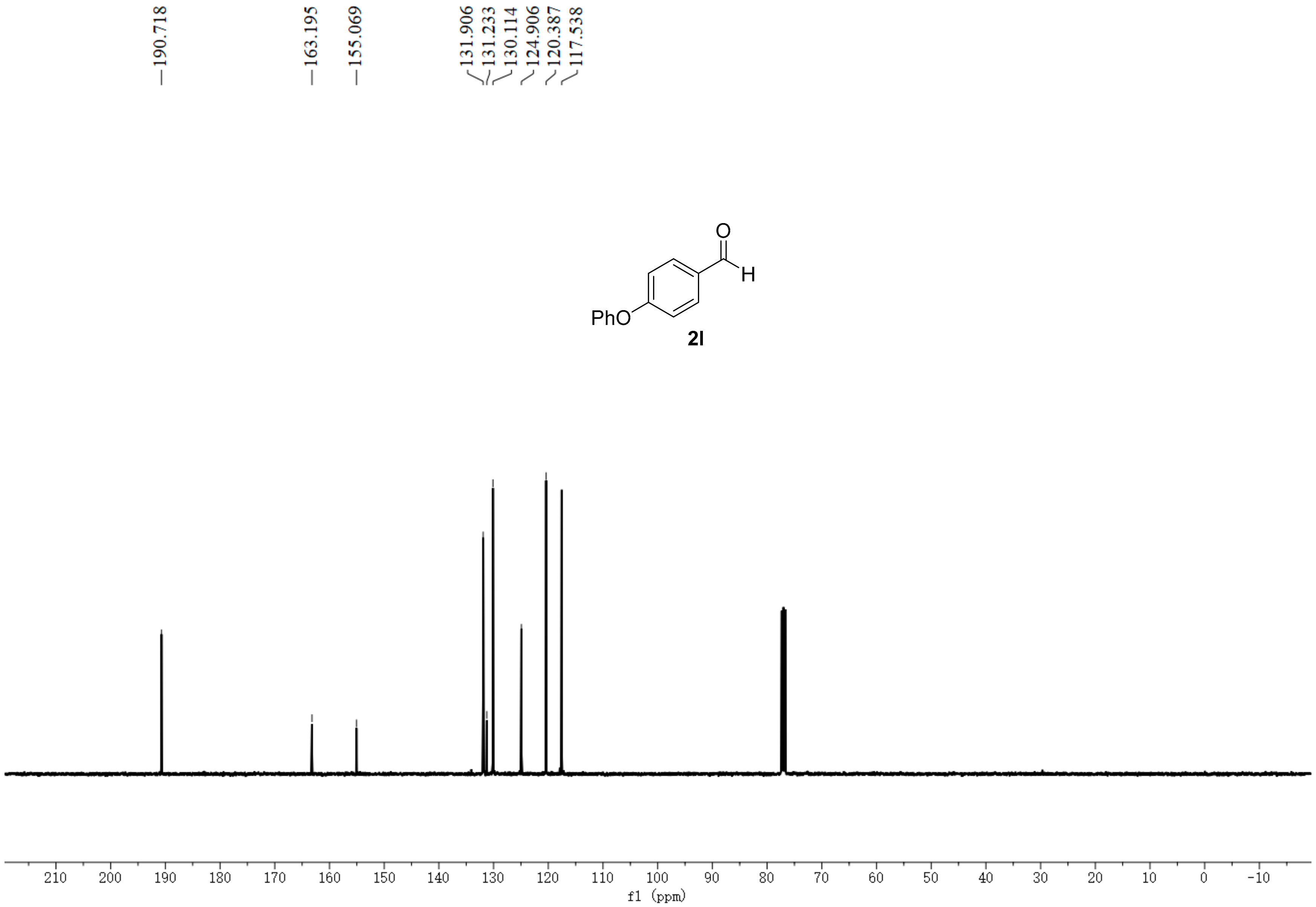

^{1}H NMR spectra of compound **2m** (400 MHz, $CDCl_3$)

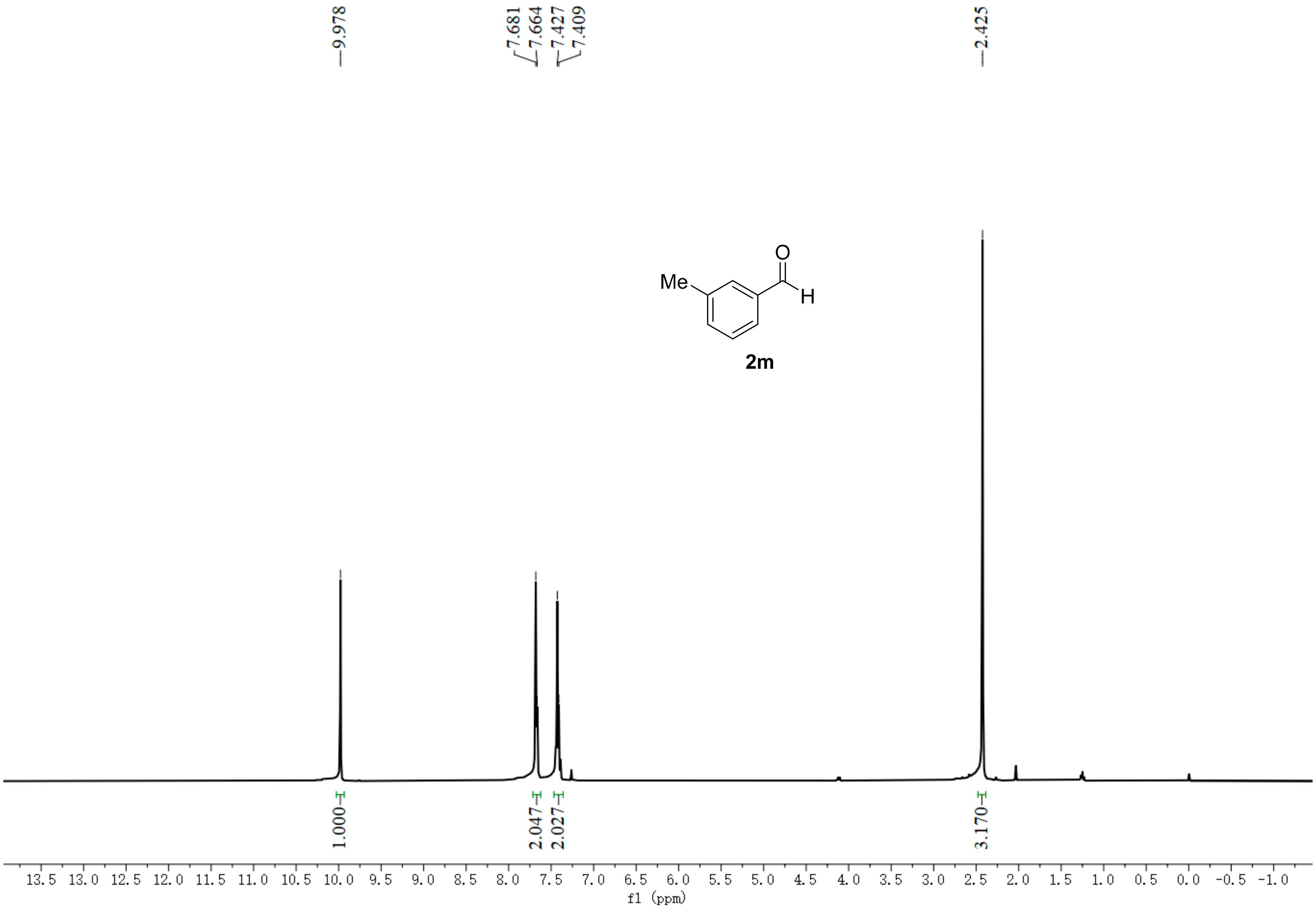

^{13}C NMR spectra of compound **2m** (101 MHz, $CDCl_3$)

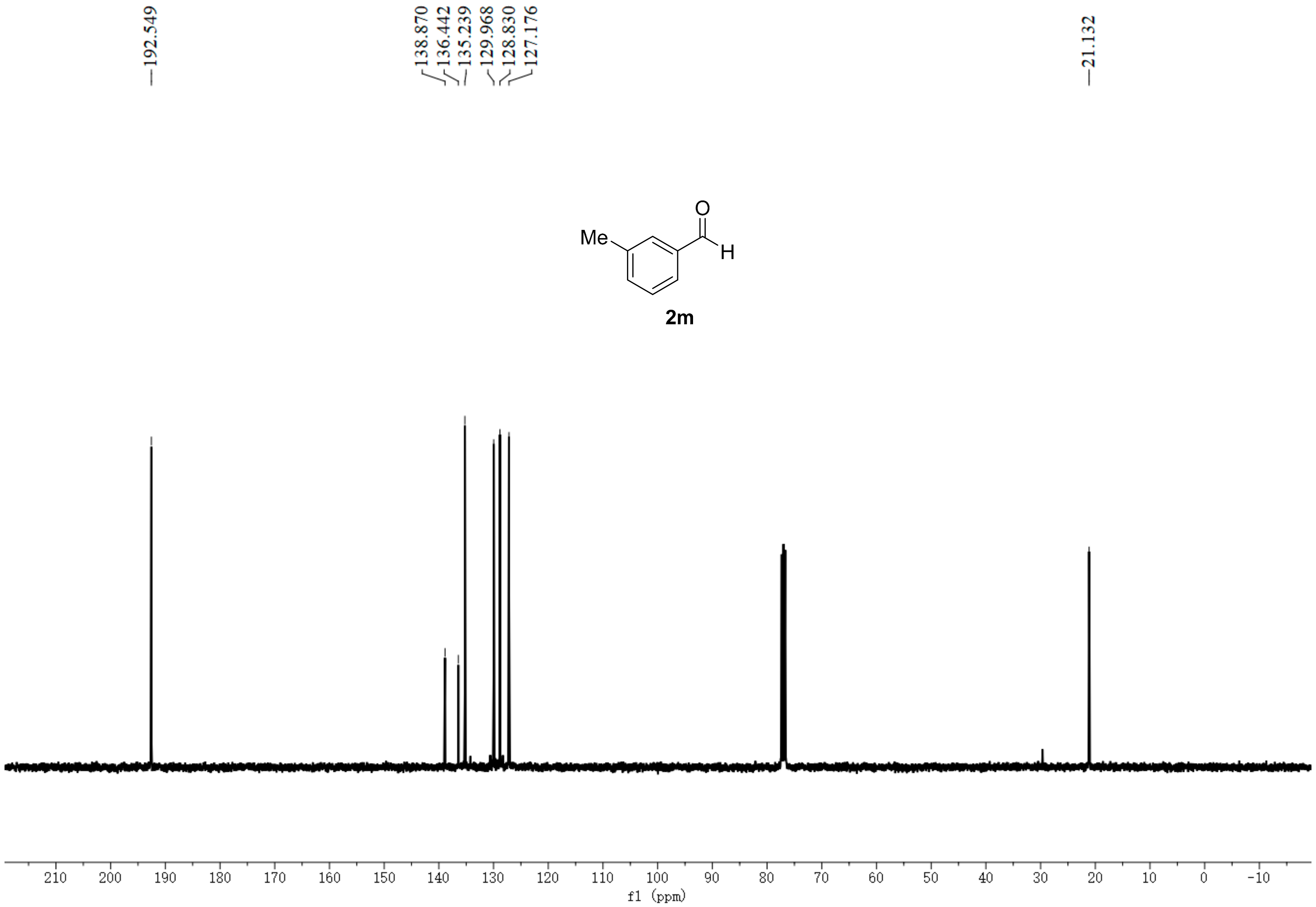

^{1}H NMR spectra of compound **2n** (400 MHz, $CDCl_3$)

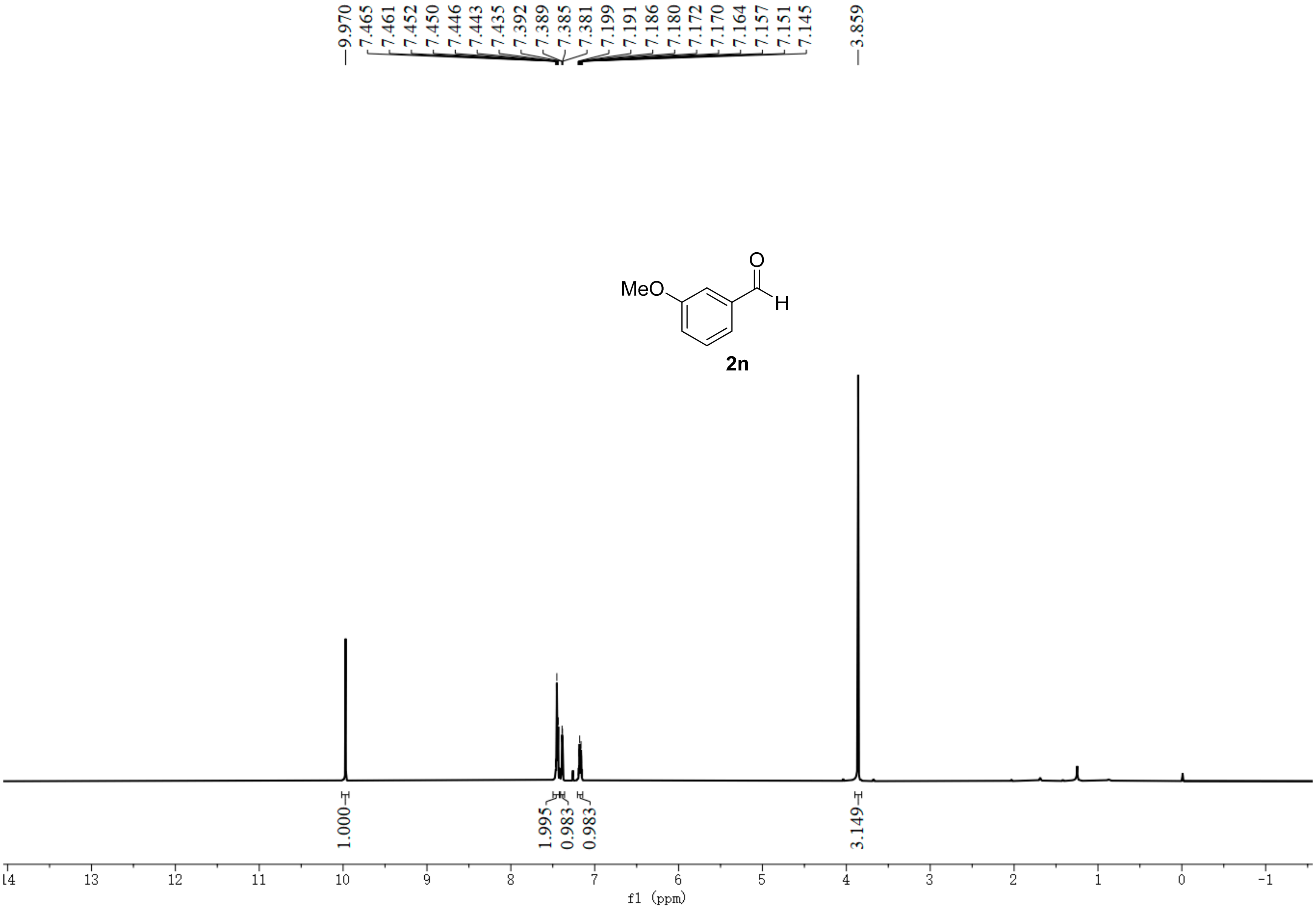

^{13}C NMR spectra of compound **2n** (101 MHz, $CDCl_3$)

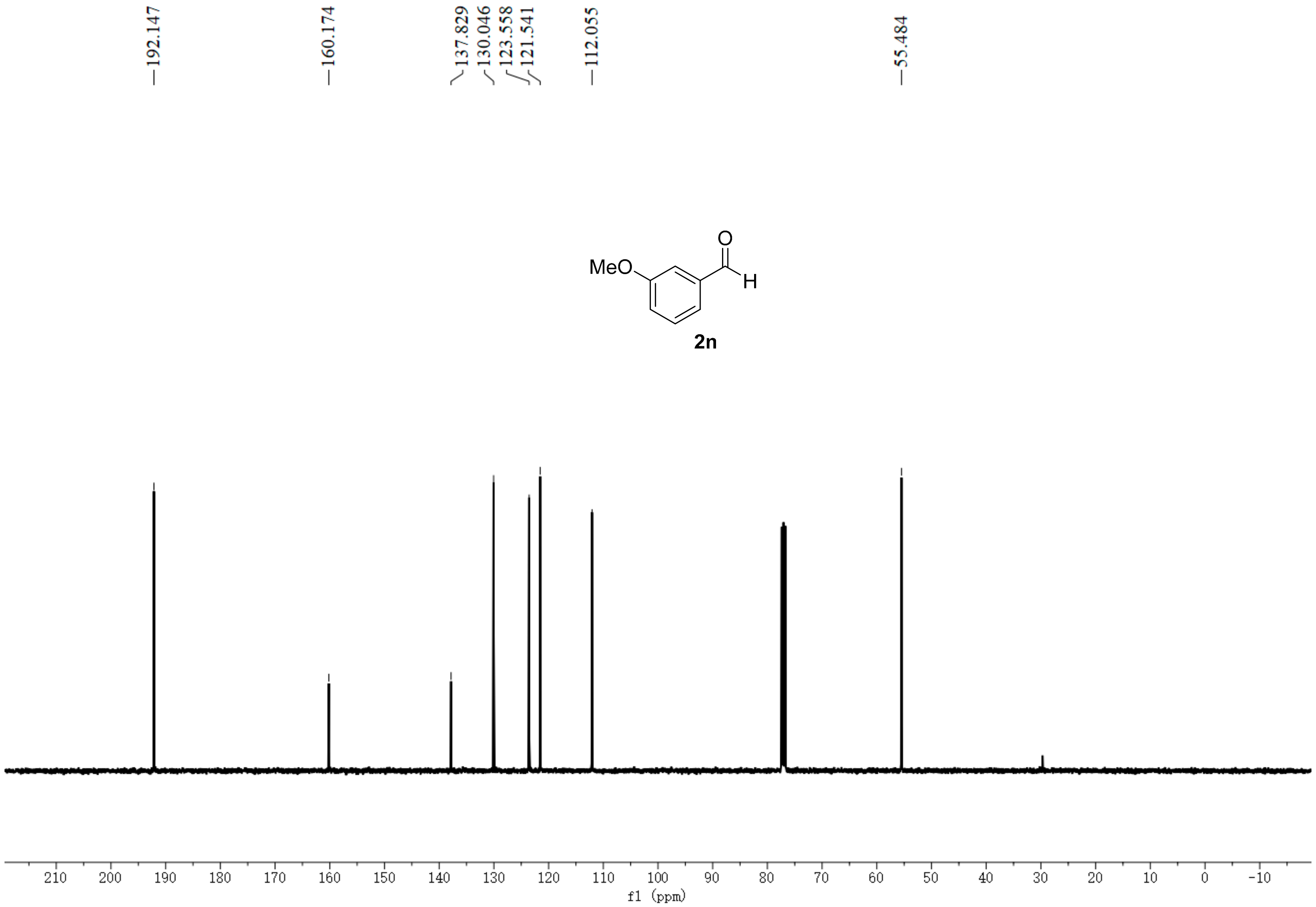

^{1}H NMR spectra of compound **2o** (400 MHz, $CDCl_3$)

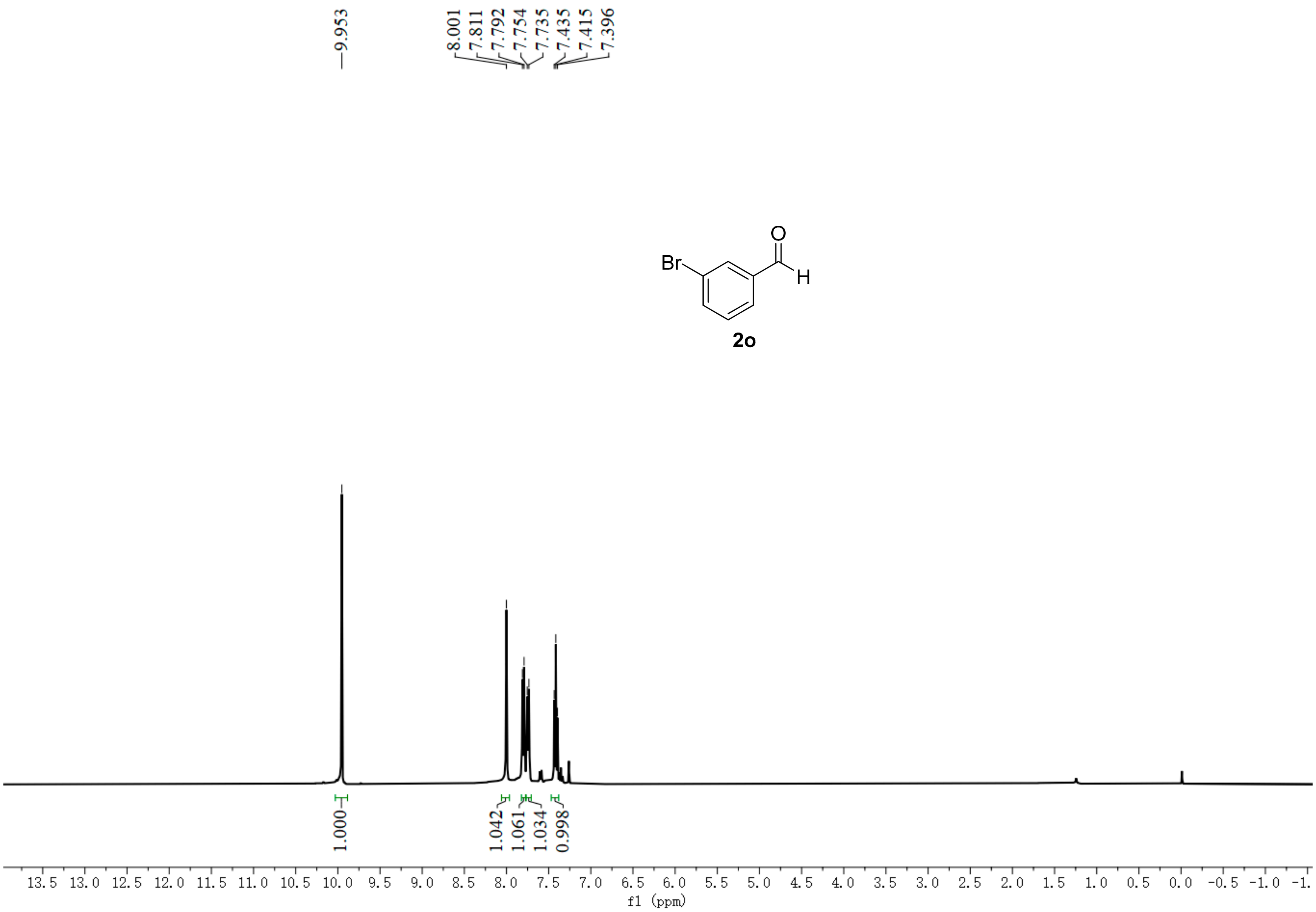

^{13}C NMR spectra of compound **2o** (101 MHz, CDCl$_3$)

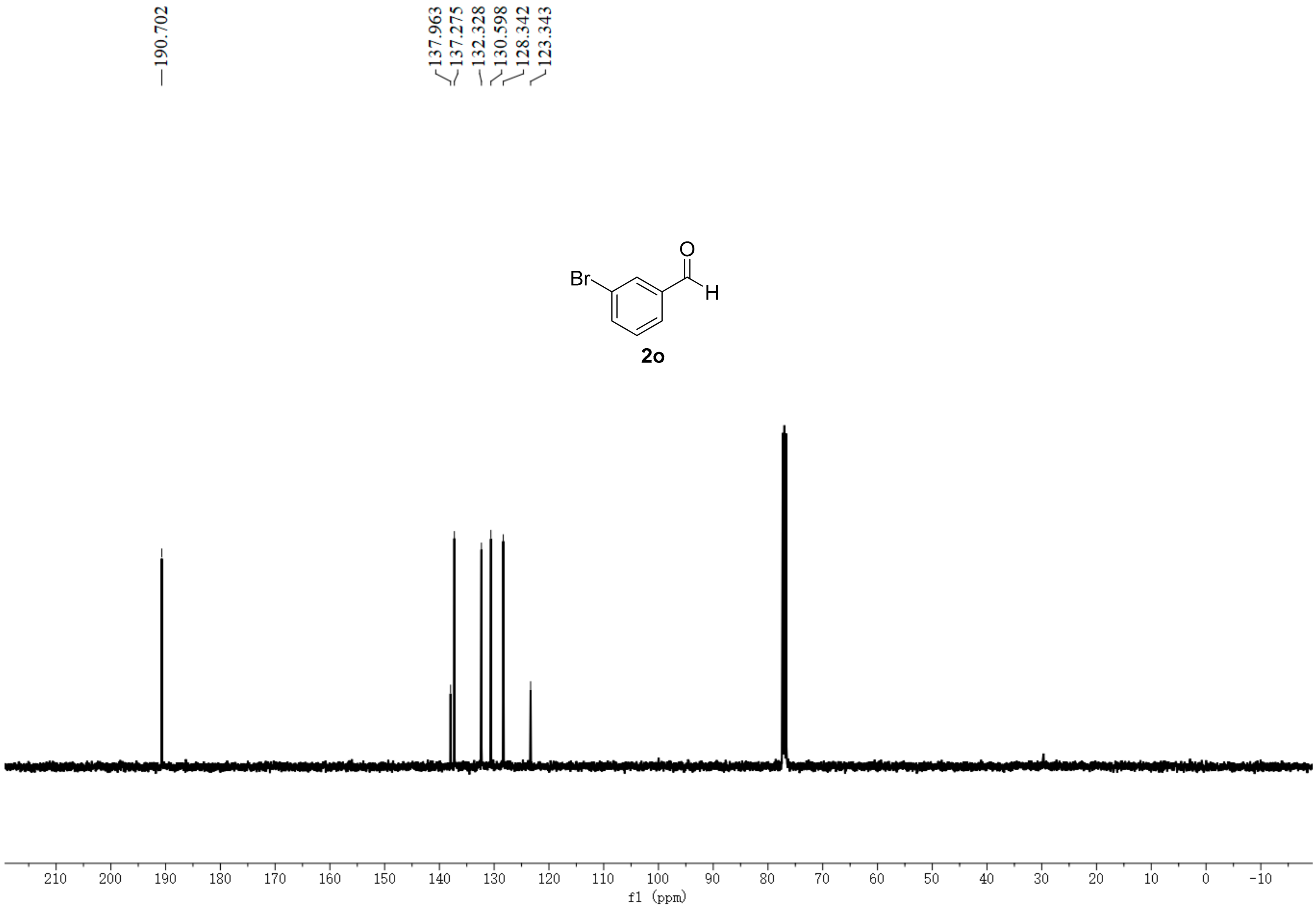

^{1}H NMR spectra of compound **2p** (400 MHz, $CDCl_3$)

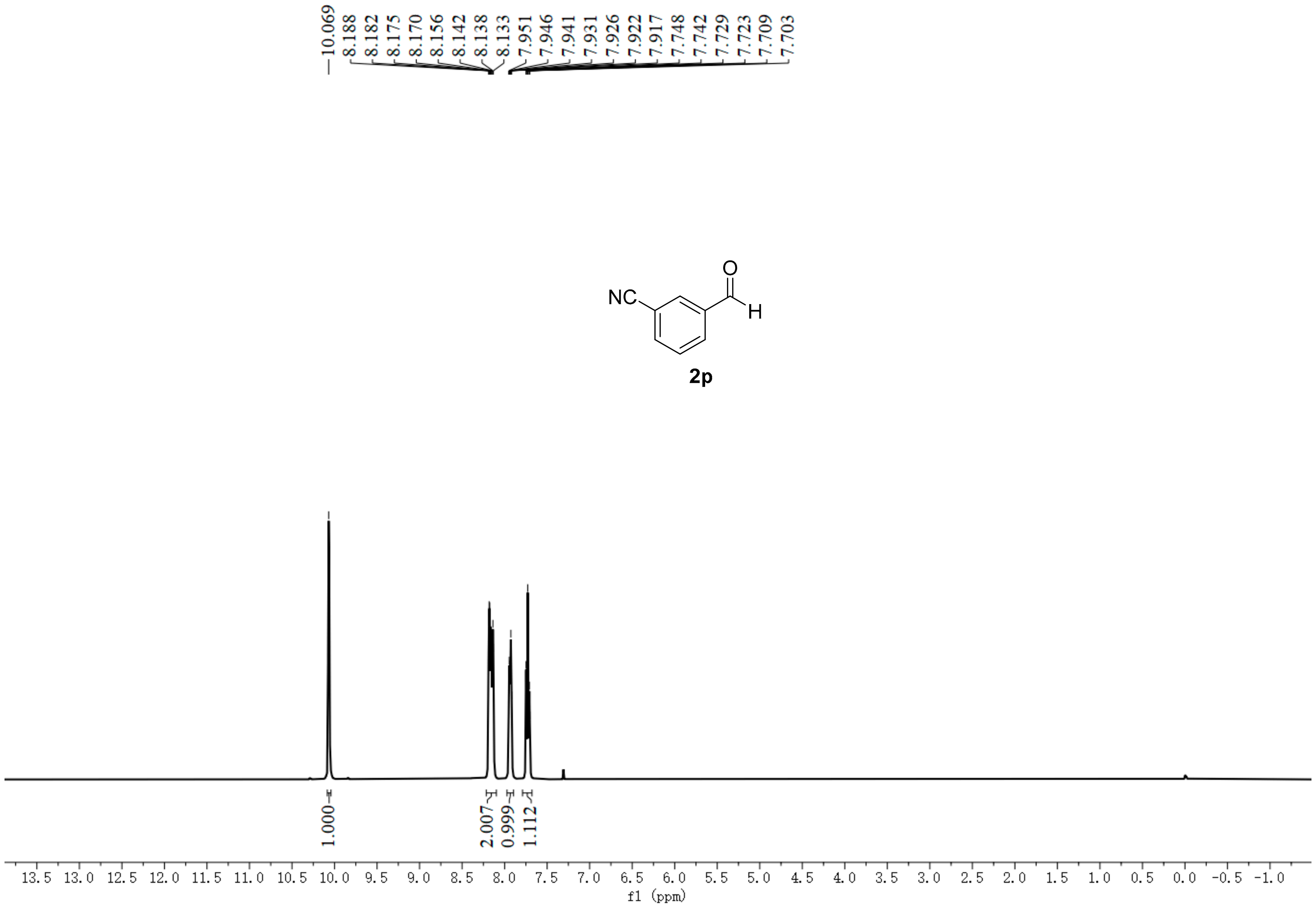

^{13}C NMR spectra of compound **2p** (101 MHz, $CDCl_3$)

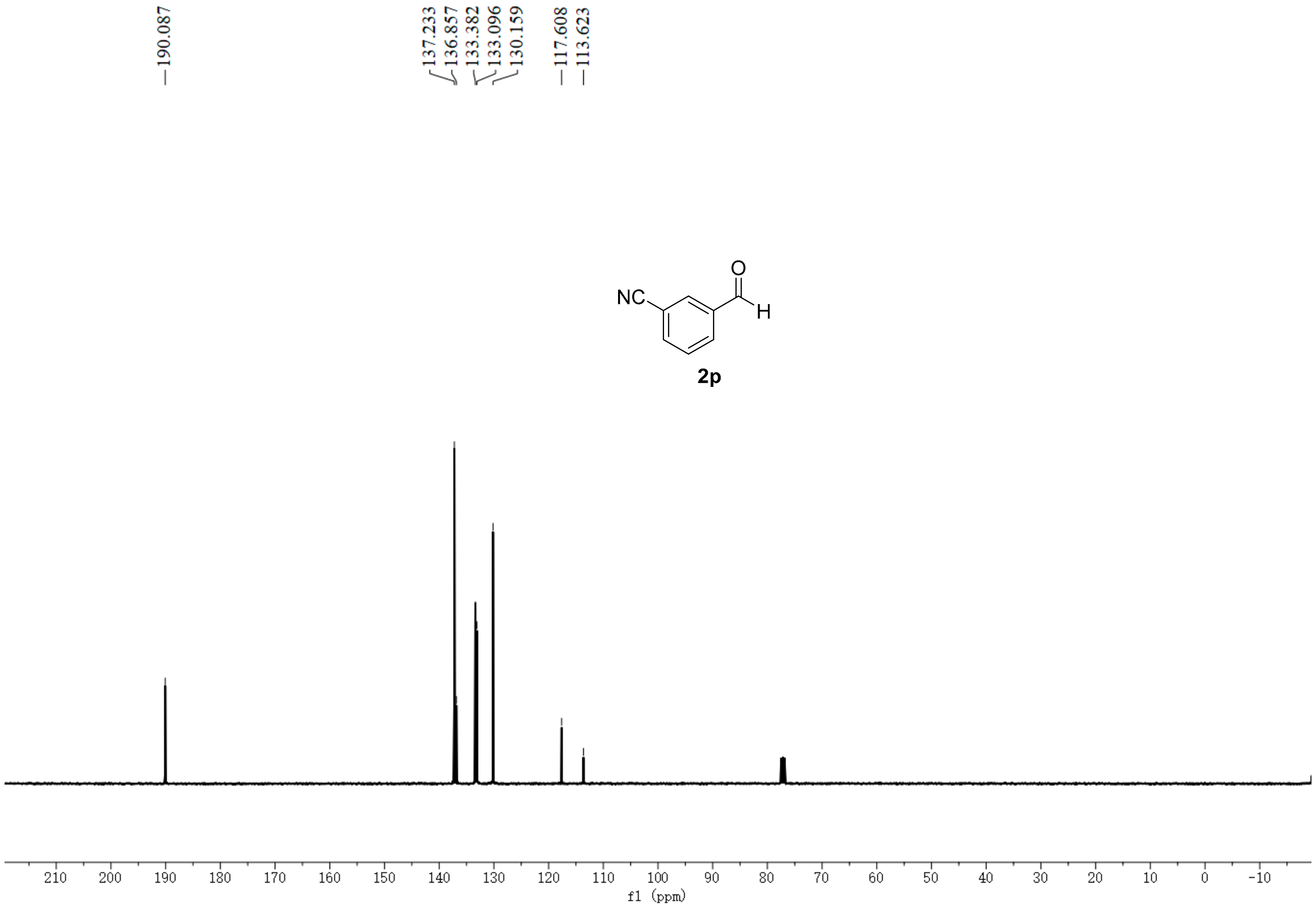

^{1}H NMR spectra of compound **2q** (400 MHz, $CDCl_3$)

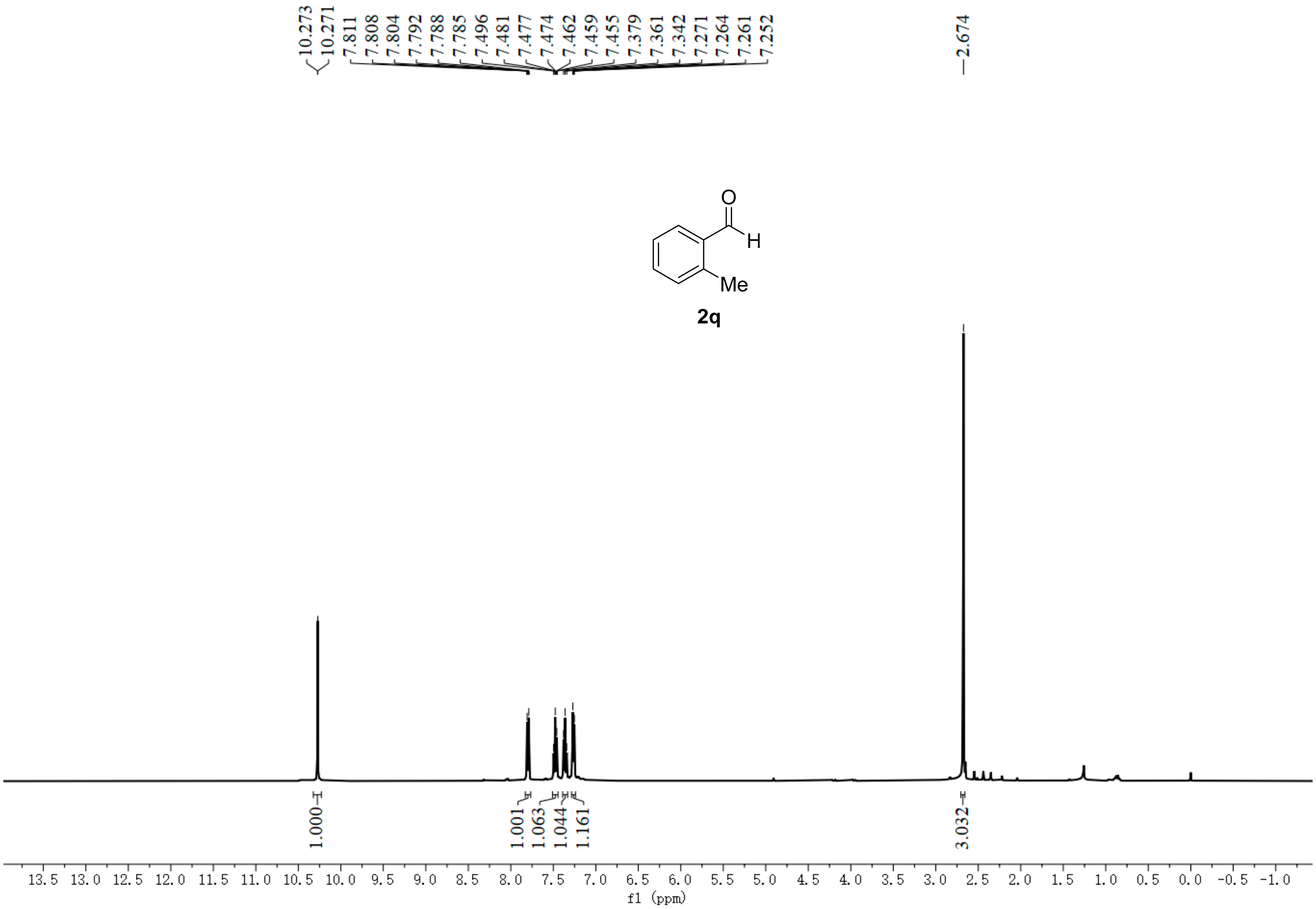

^{13}C NMR spectra of compound **2q** (101 MHz, $CDCl_3$)

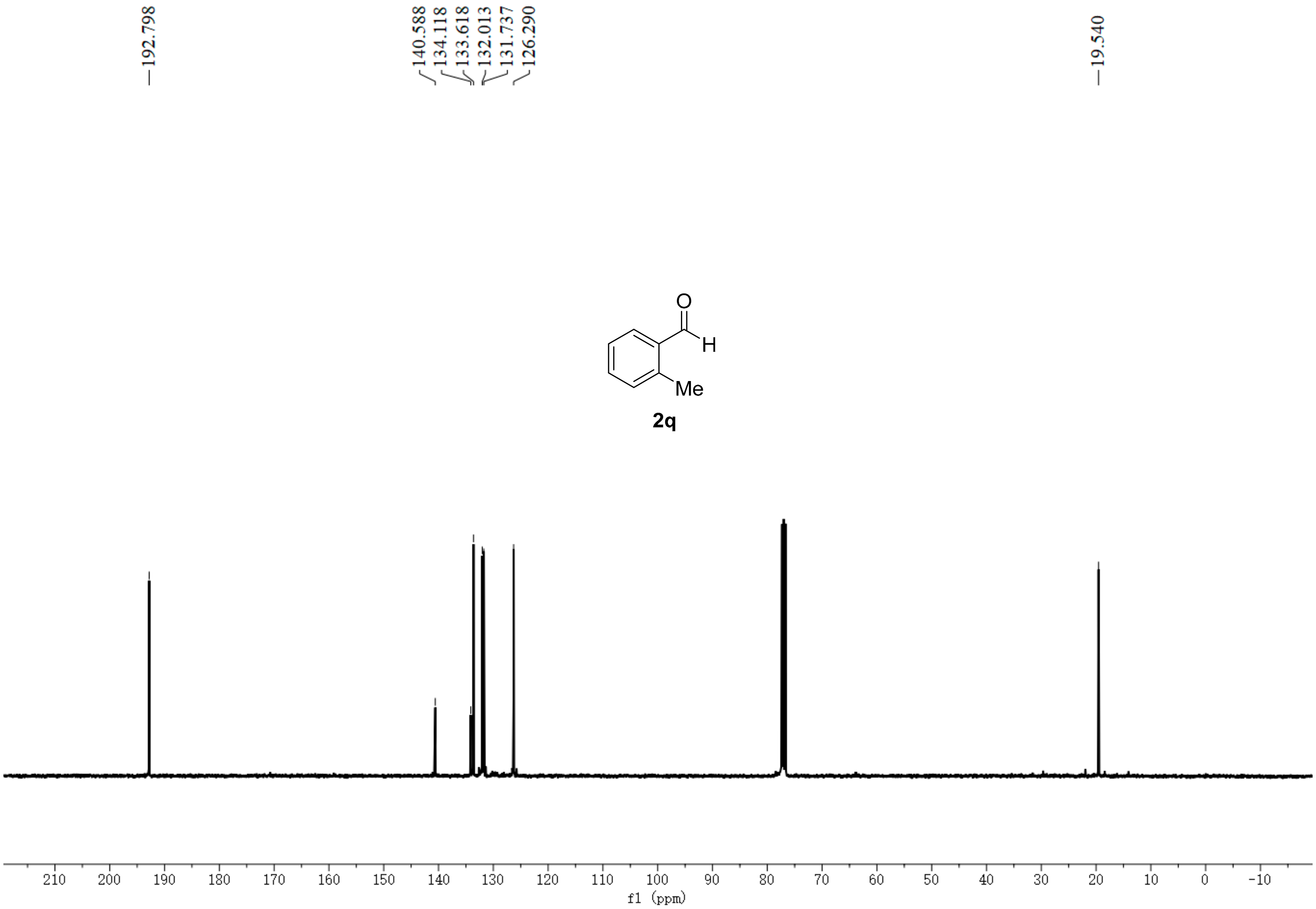

^{1}H NMR spectra of compound **2r** (400 MHz, $CDCl_3$)

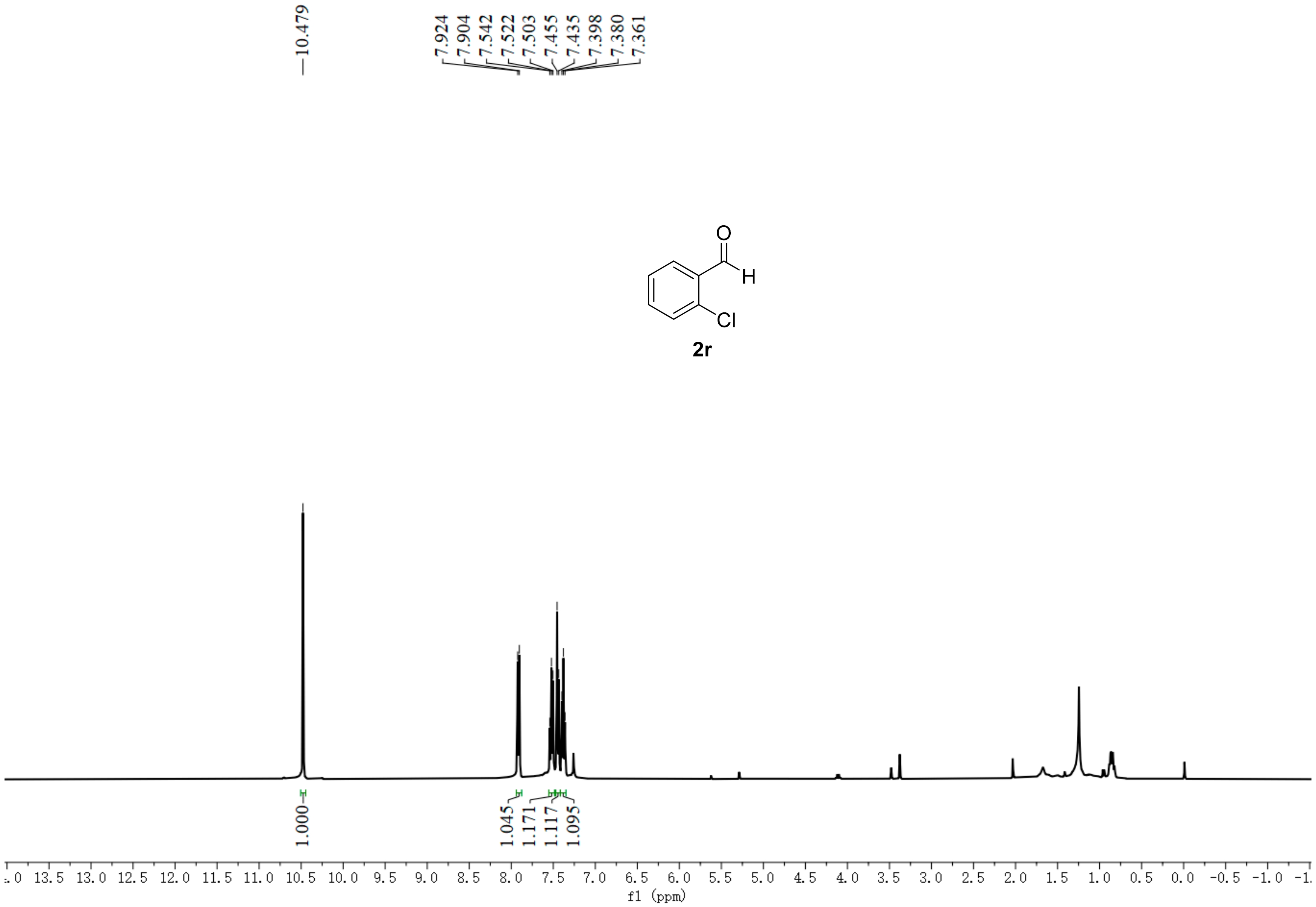

^{13}C NMR spectra of compound **2r** (101 MHz, $CDCl_3$)

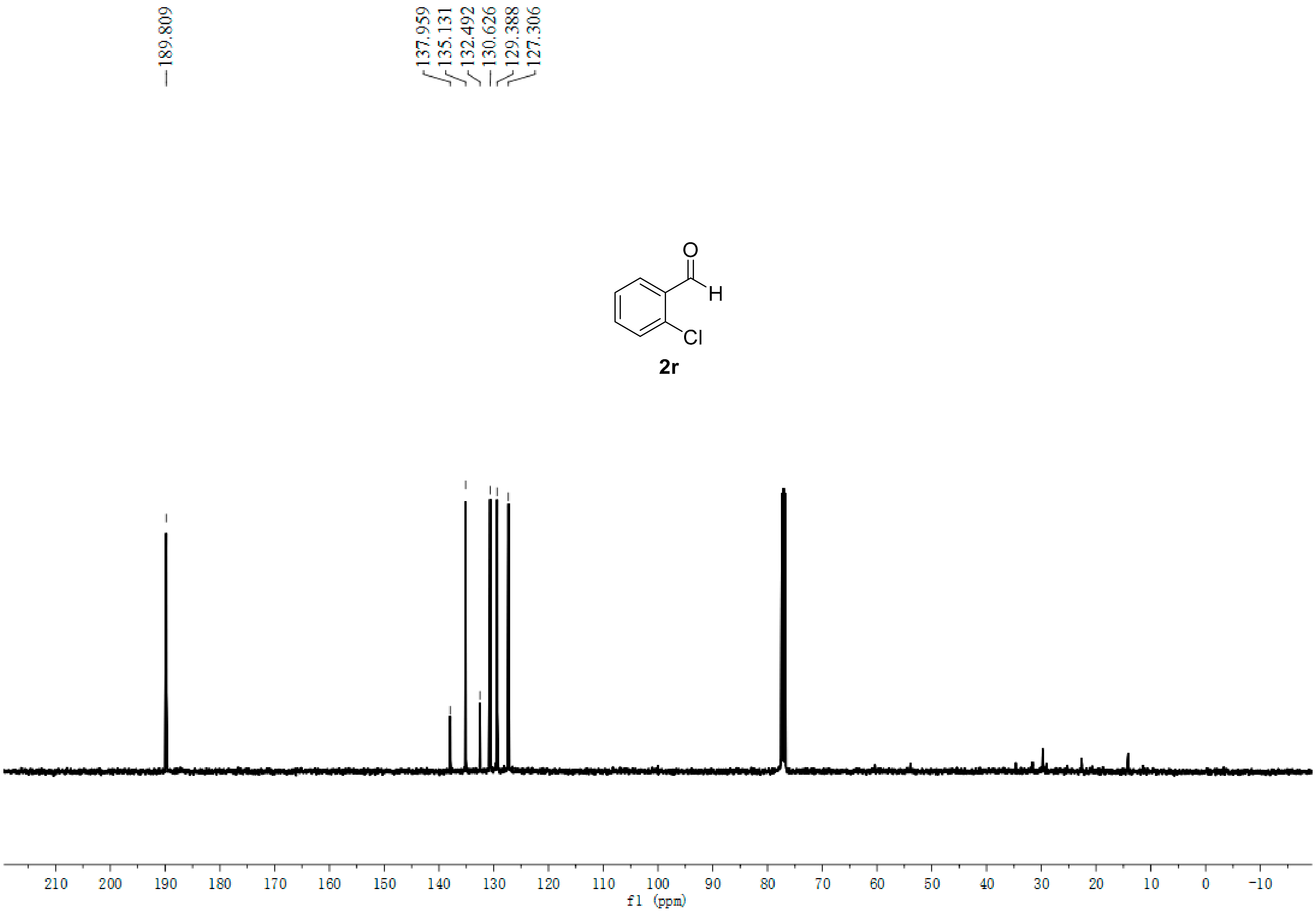

^{1}H NMR spectra of compound **2s** (400 MHz, $CDCl_3$)

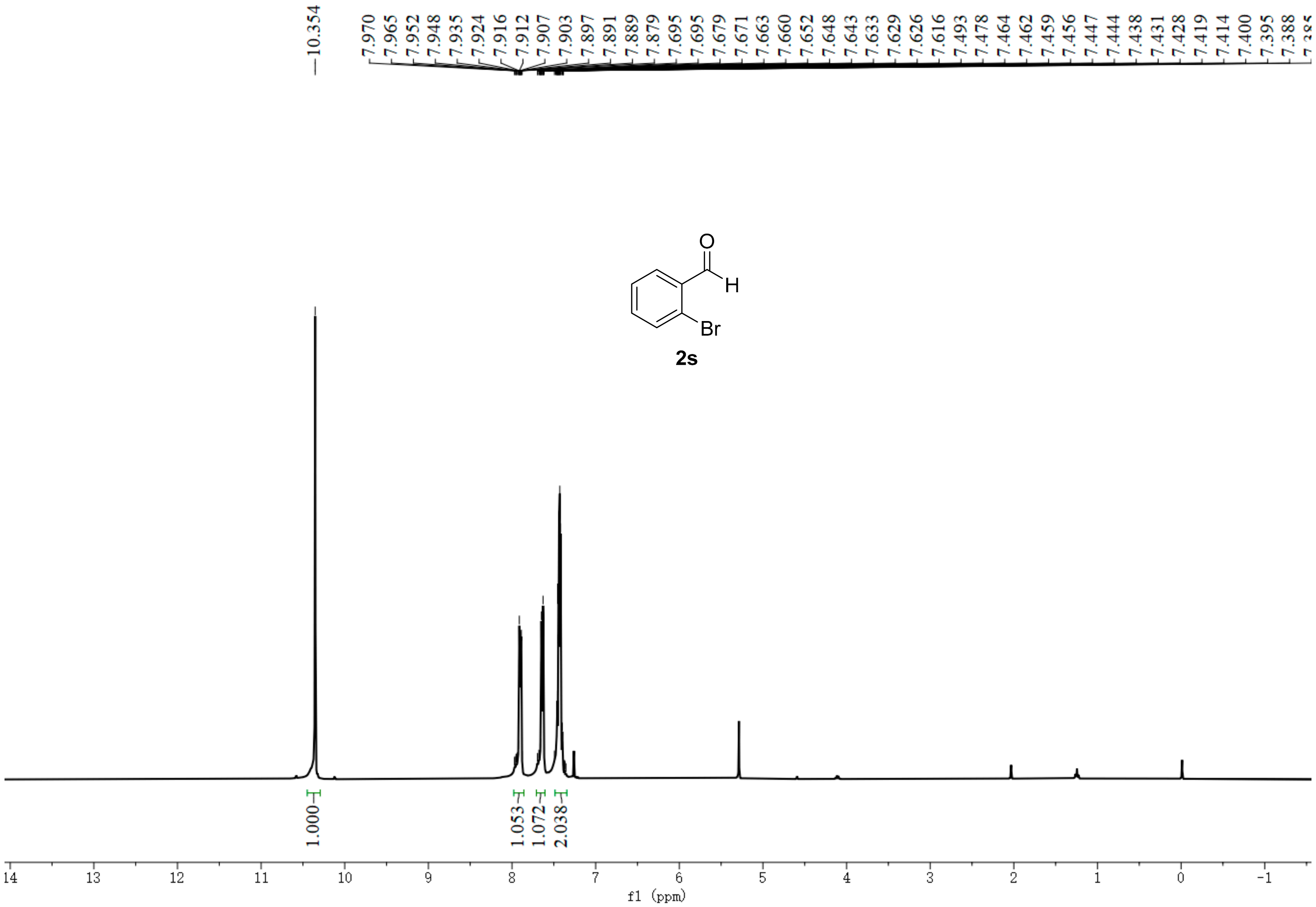

^{13}C NMR spectra of compound **2s** (101 MHz, $CDCl_3$)

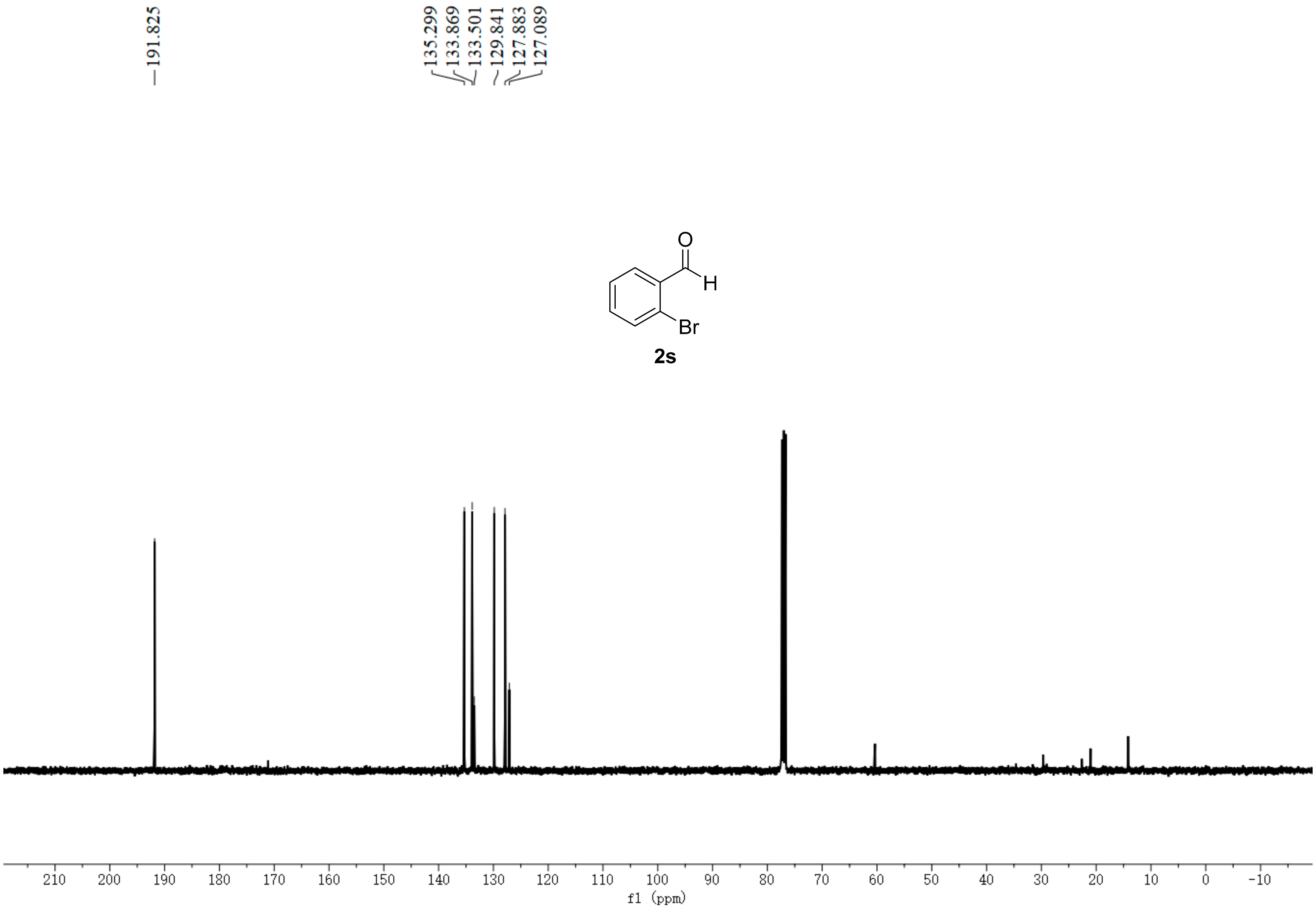

^{1}H NMR spectra of compound **2t** (400 MHz, $CDCl_3$)

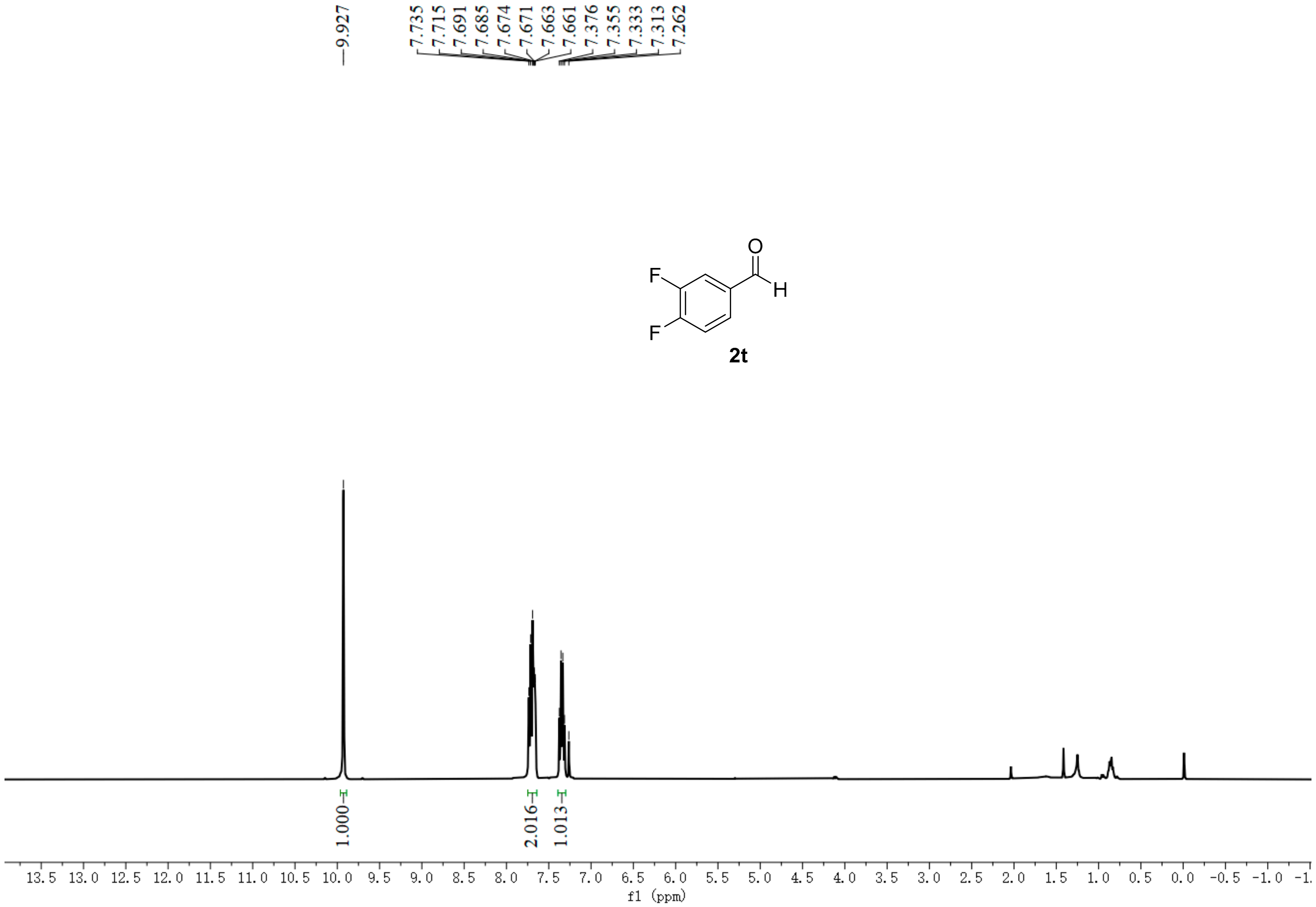

^{13}C NMR spectra of compound **2t** (101 MHz, $CDCl_3$)

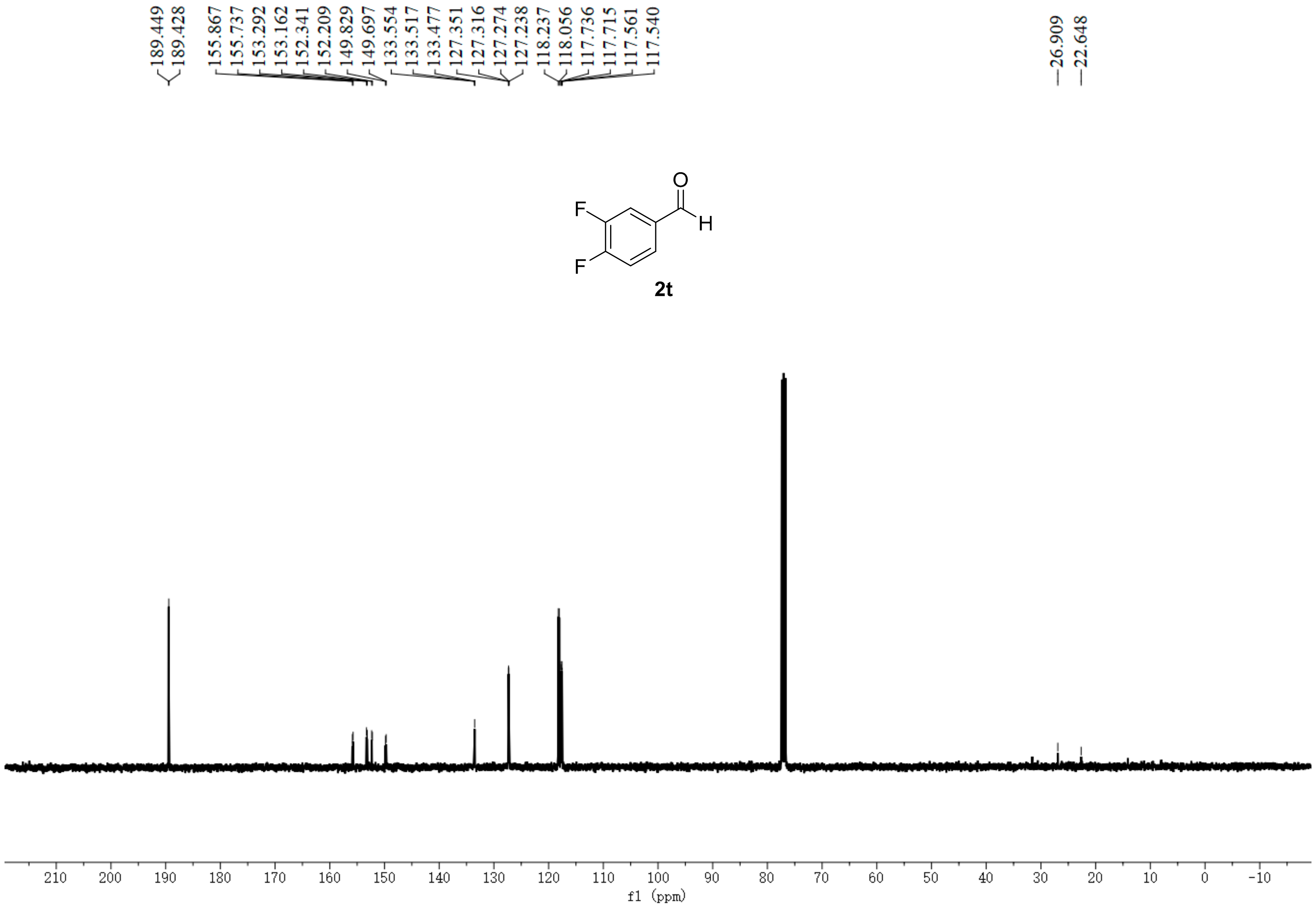

^{19}F NMR spectra of compound **2t** (376 MHz, $CDCl_3$)

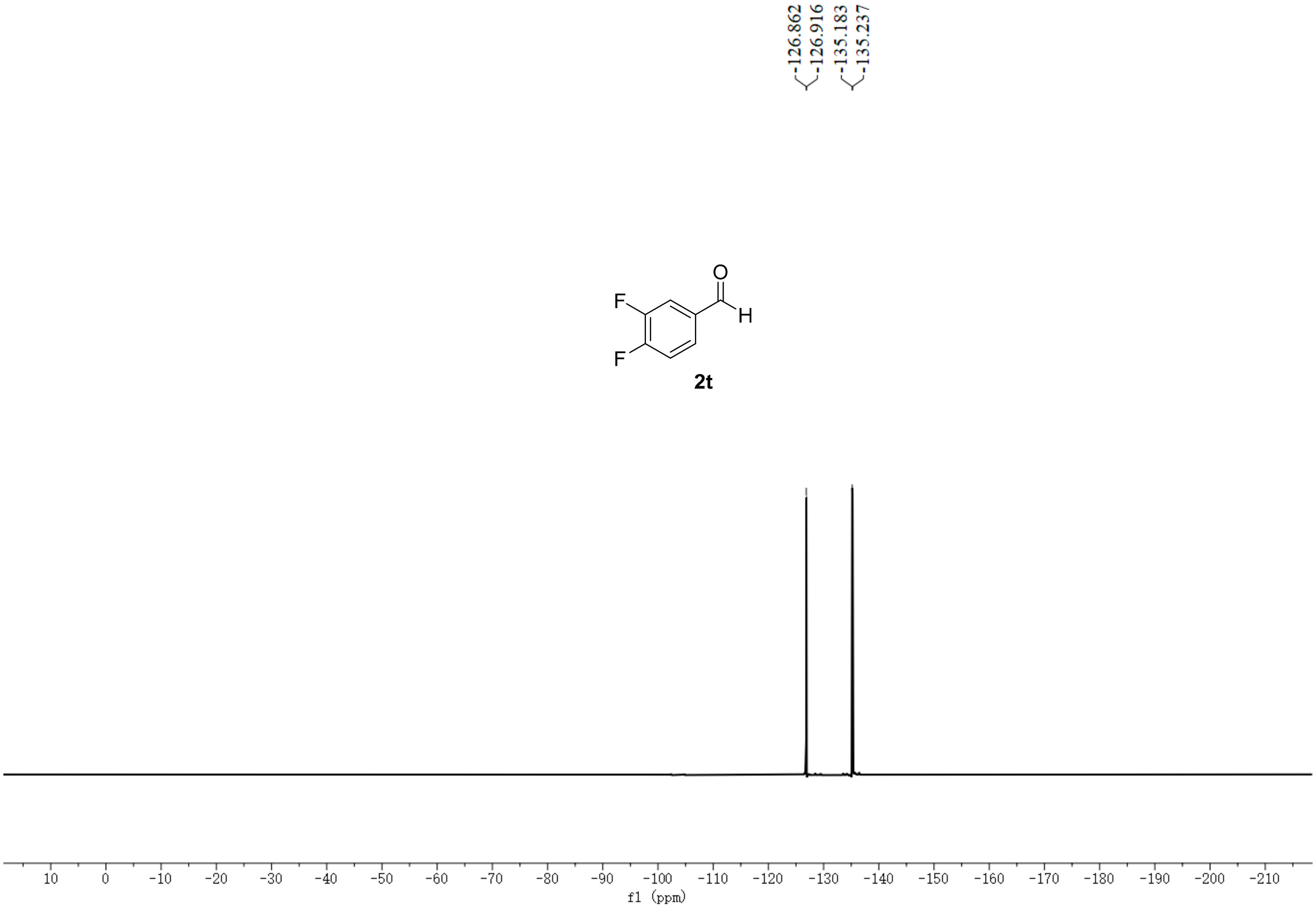

2t

^{1}H NMR spectra of compound **2u** (400 MHz, $CDCl_3$)

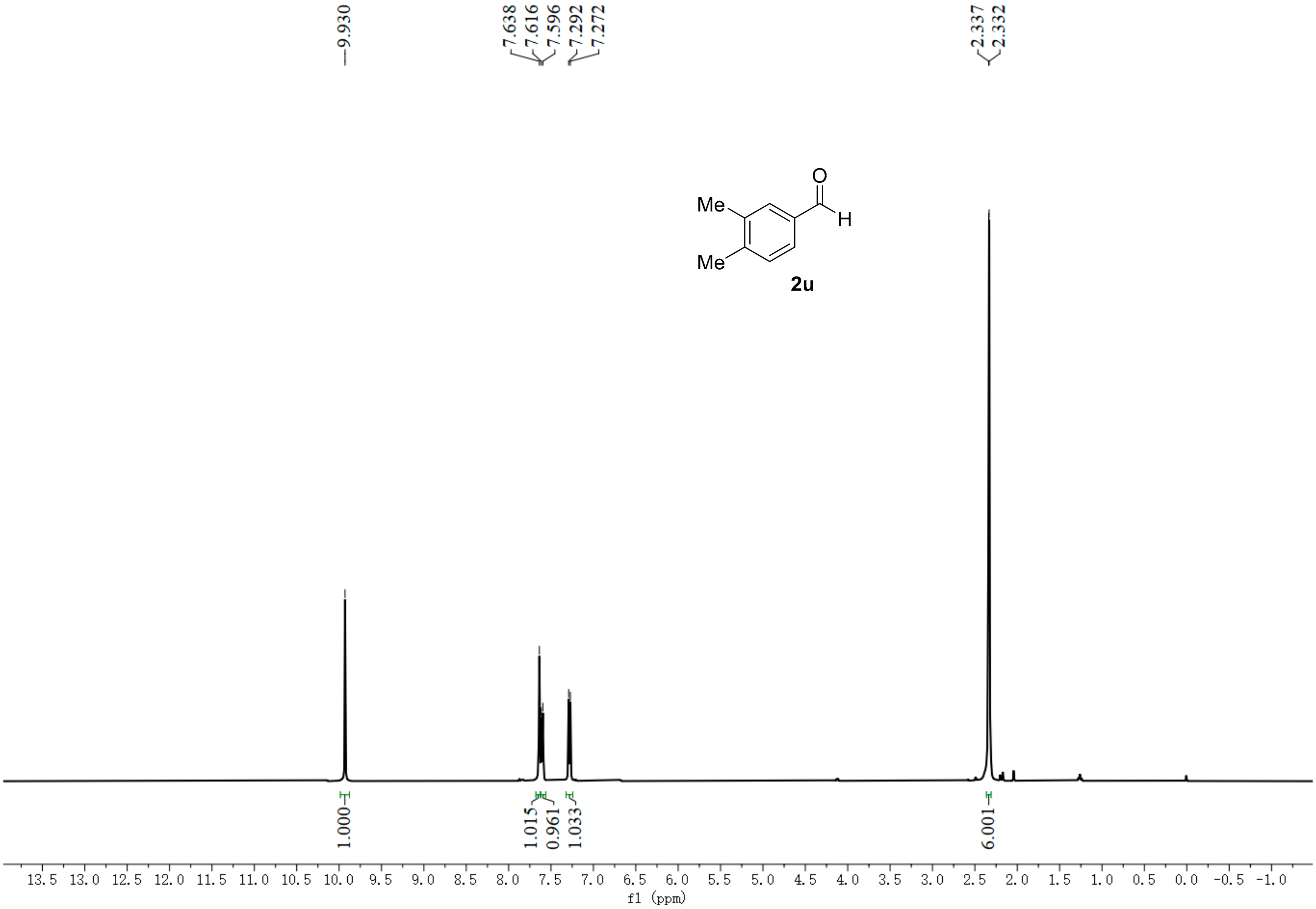

^{13}C NMR spectra of compound **2u** (101 MHz, $CDCl_3$)

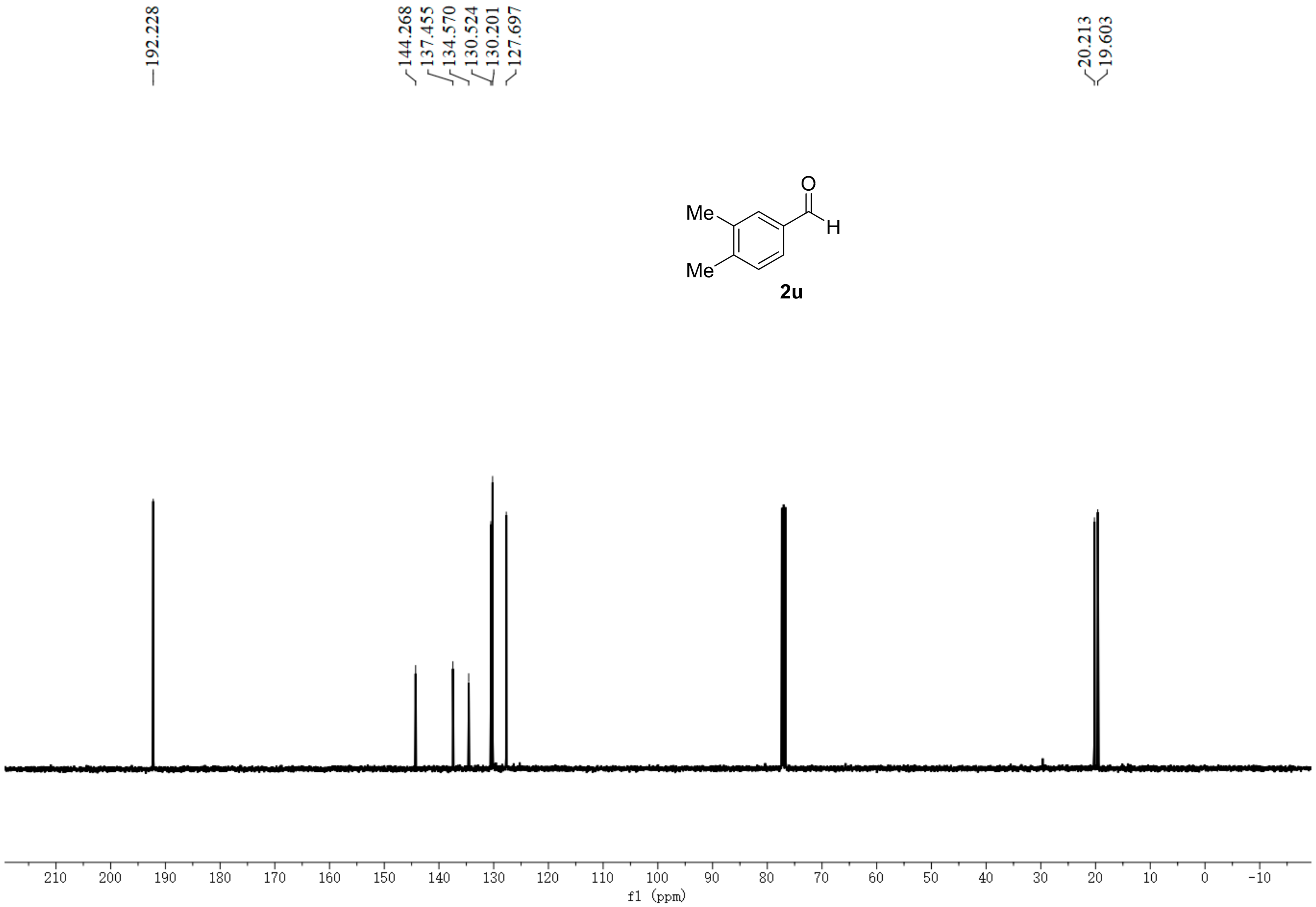

^{1}H NMR spectra of compound **2v** (400 MHz, $CDCl_3$)

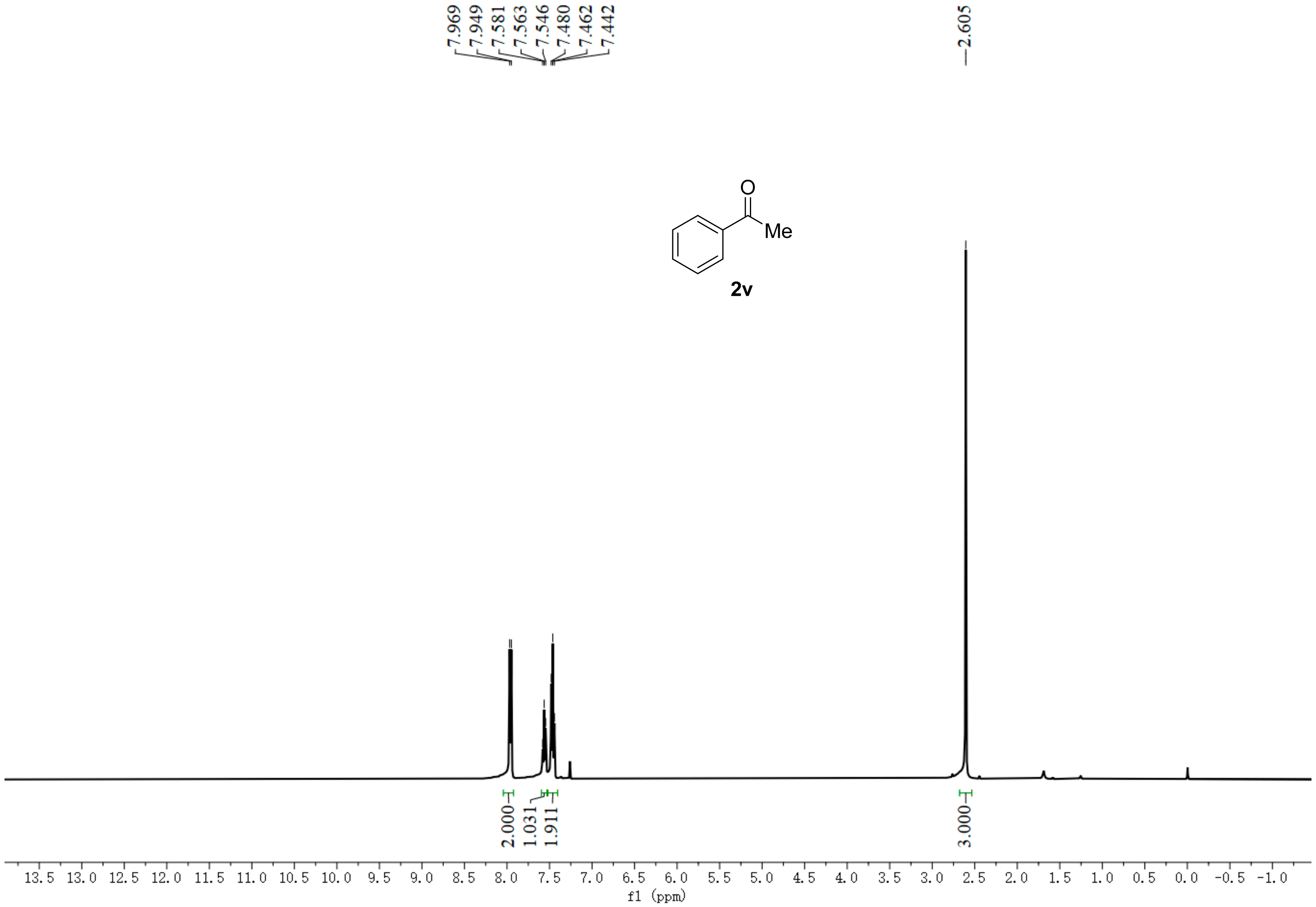

^{13}C NMR spectra of compound **2v** (101 MHz, $CDCl_3$)

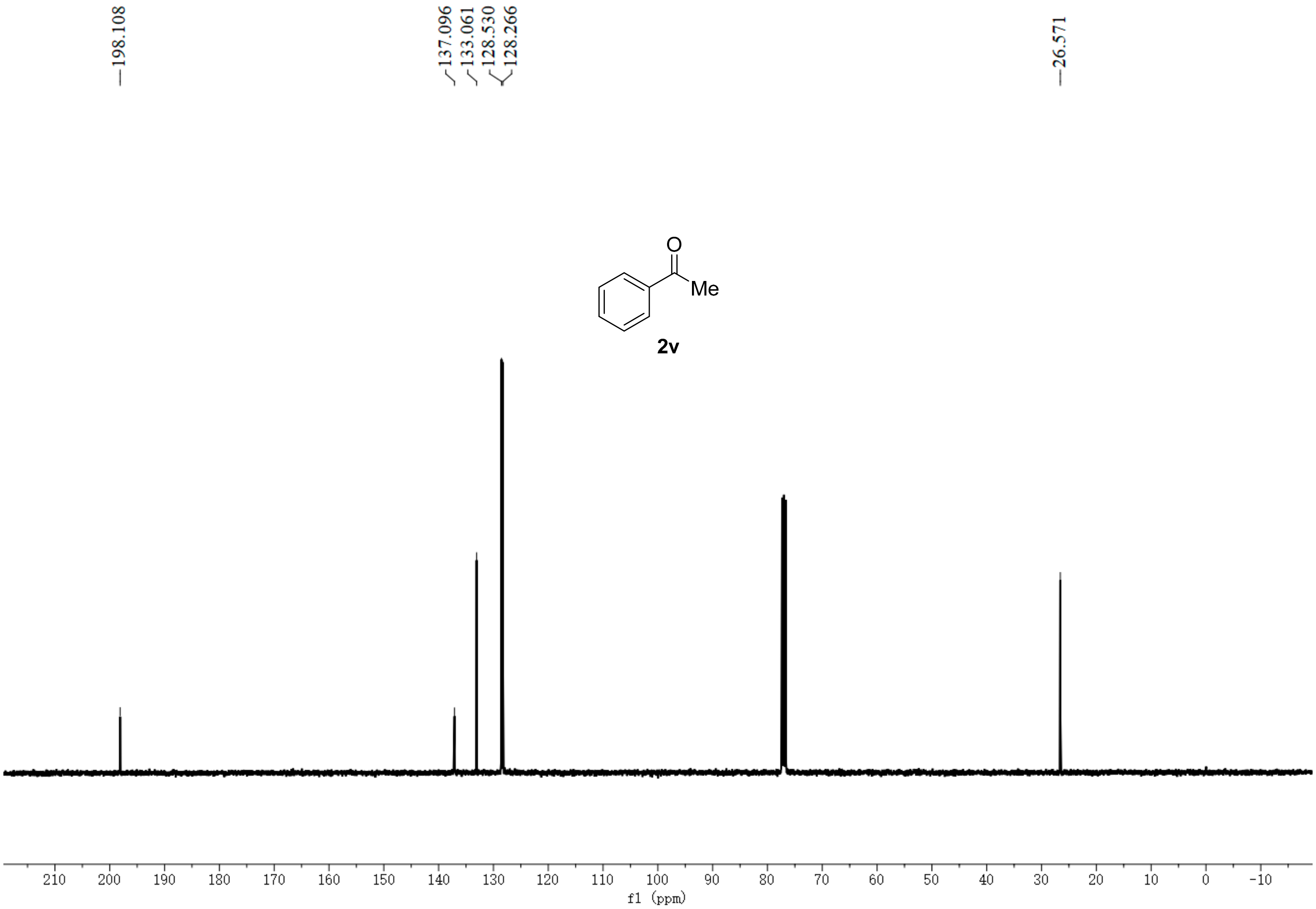

^{1}H NMR spectra of compound **2w** (400 MHz, $CDCl_3$)

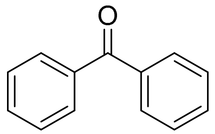

2w

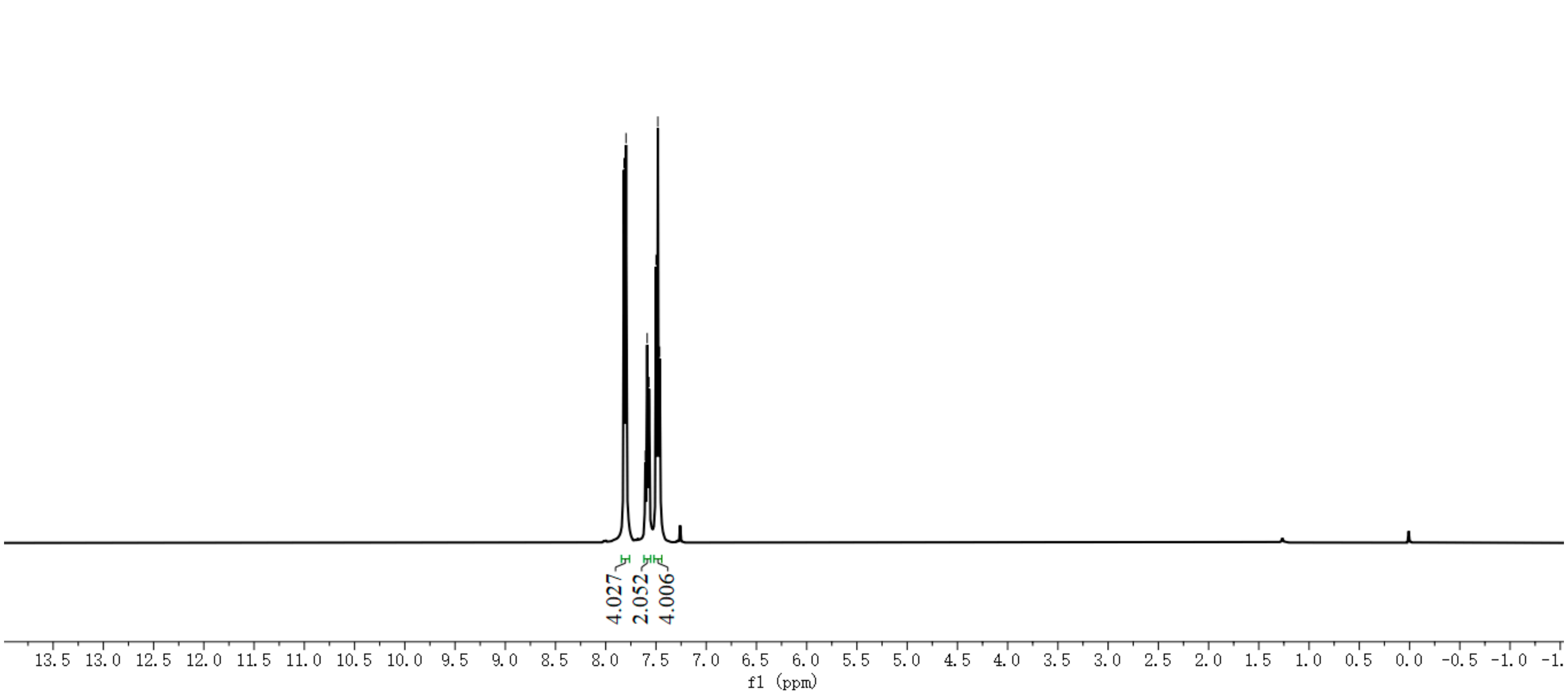

^{13}C NMR spectra of compound **2w** (101 MHz, $CDCl_3$)

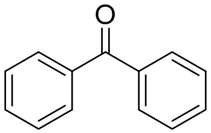

2w

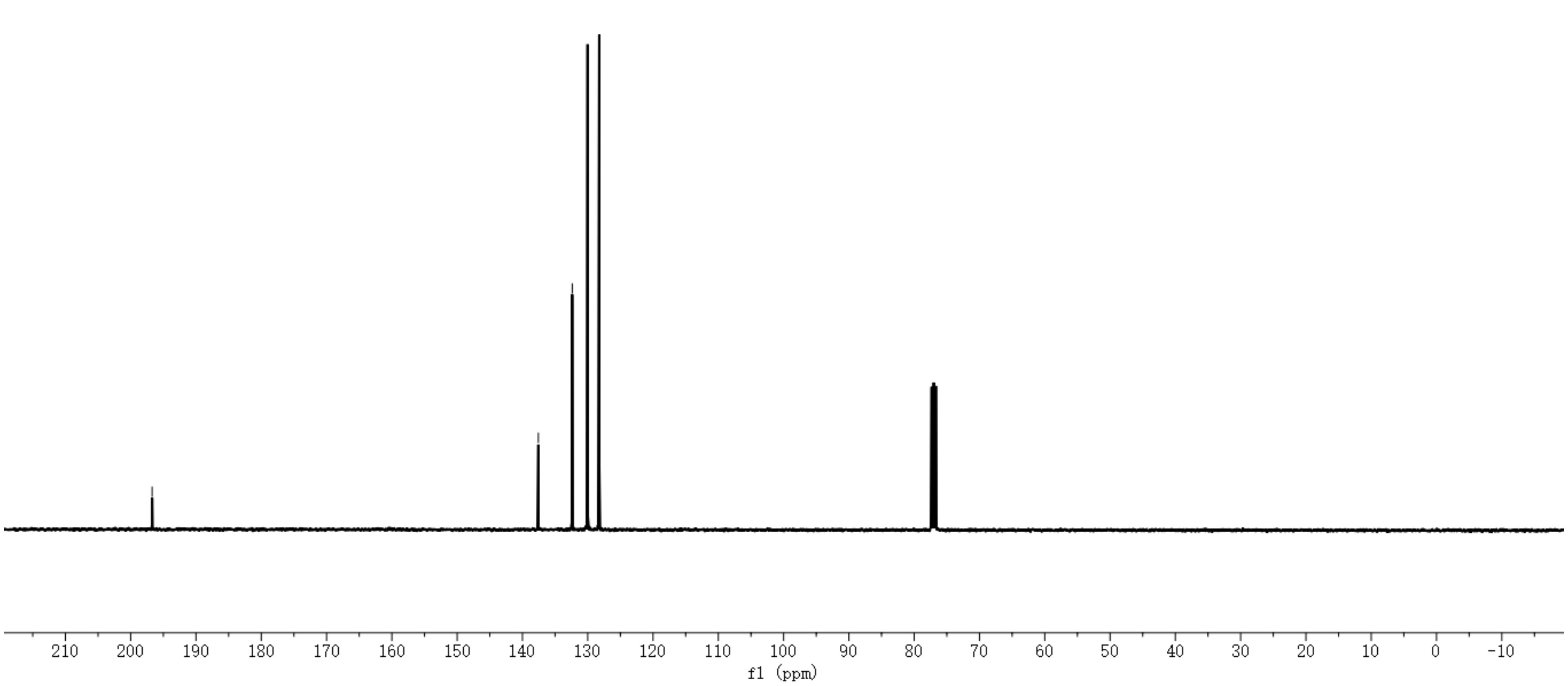

^1H NMR spectra of compound **2x** (400 MHz, $CDCl_3$)

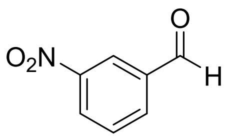

2x

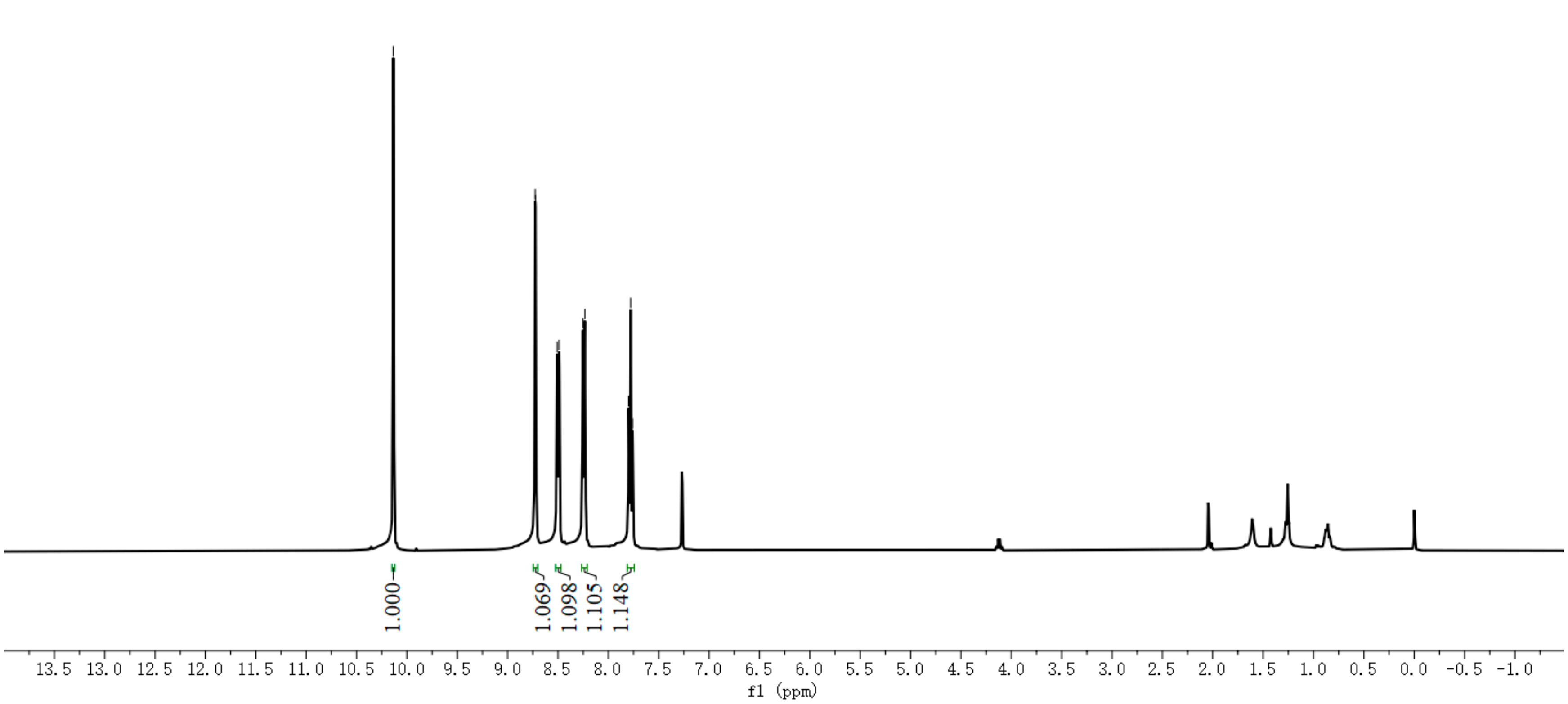

^{13}C NMR spectra of compound **2x** (101 MHz, $CDCl_3$)

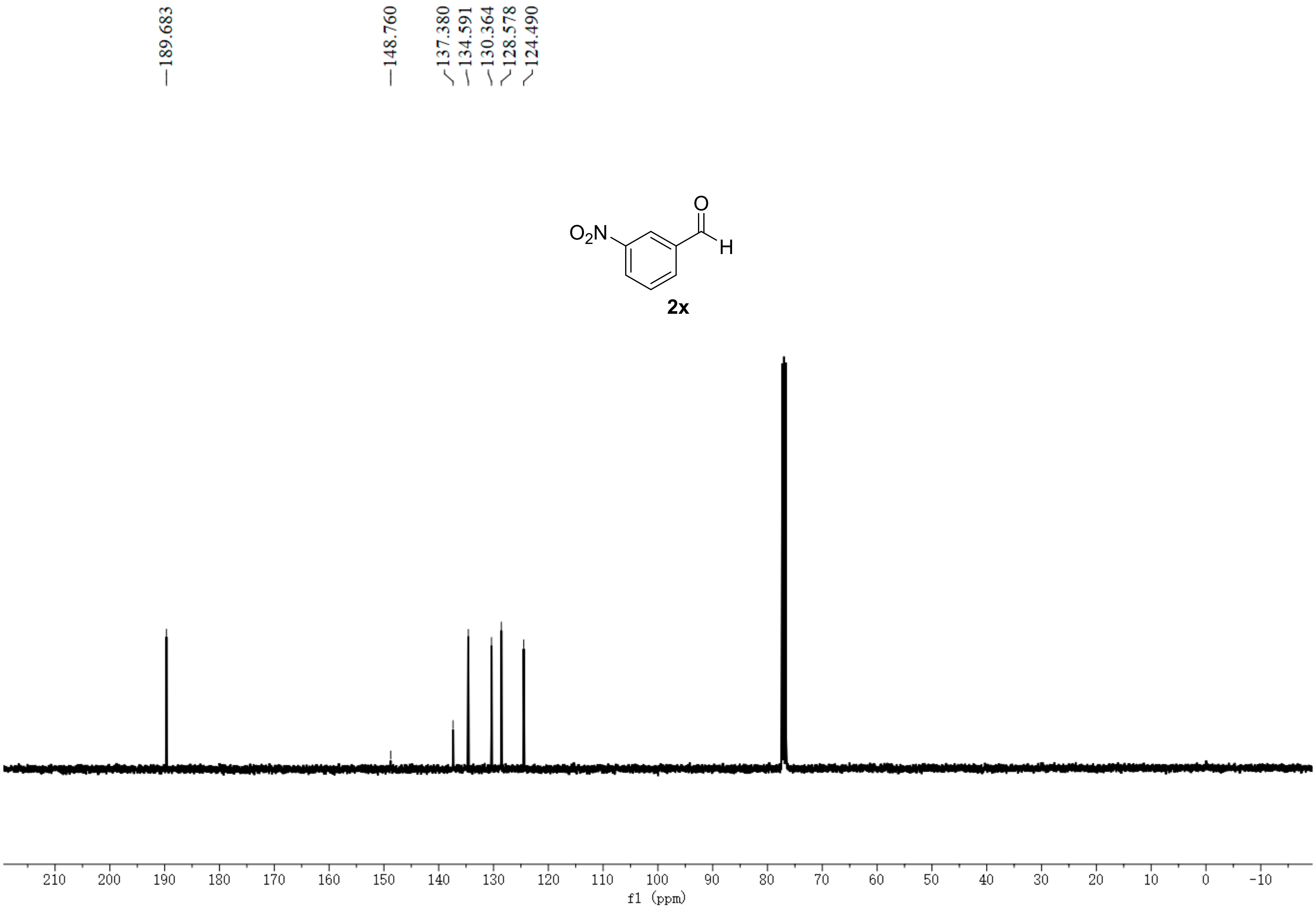

Supplement: Supplementary file 1 [file molecules-29-02851-s001.zip › molecules-3053421-supplementary.pdf]
